# Supplementary material for: Myosin II Reactivation and Cytoskeletal Remodeling as a Hallmark and a Vulnerability in Melanoma Therapy Resistance
Source: Cancer Cell. 2020 Jan 13;37(1):85–103.e9. doi: 10.1016/j.ccell.2019.12.003 (PMC6958528; doi:10.1016/j.ccell.2019.12.003)
Supplement: Document S1. Figures S1–S8 and Tables S5–S7 [file mmc1.pdf]

Cancer Cell, Volume *37*

Supplemental Information

Myosin II Reactivation and Cytoskeletal Remodeling as a Hallmark and a Vulnerability in Melanoma Therapy Resistance

Jose L. Orgaz, Eva Crosas-Molist, Amine Sadok, Anna Perdrix-Rosell, Oscar Maiques, Irene Rodriguez-Hernandez, Jo Monger, Silvia Mele, Mirella Georgouli, Victoria Bridgeman, Panagiotis Karagiannis, Rebecca Lee, Pahini Pandya, Lena Boehme, Fredrik Wallberg, Chris Tape, Sophia N. Karagiannis, Ilaria Malanchi, and Victoria Sanz-Moreno

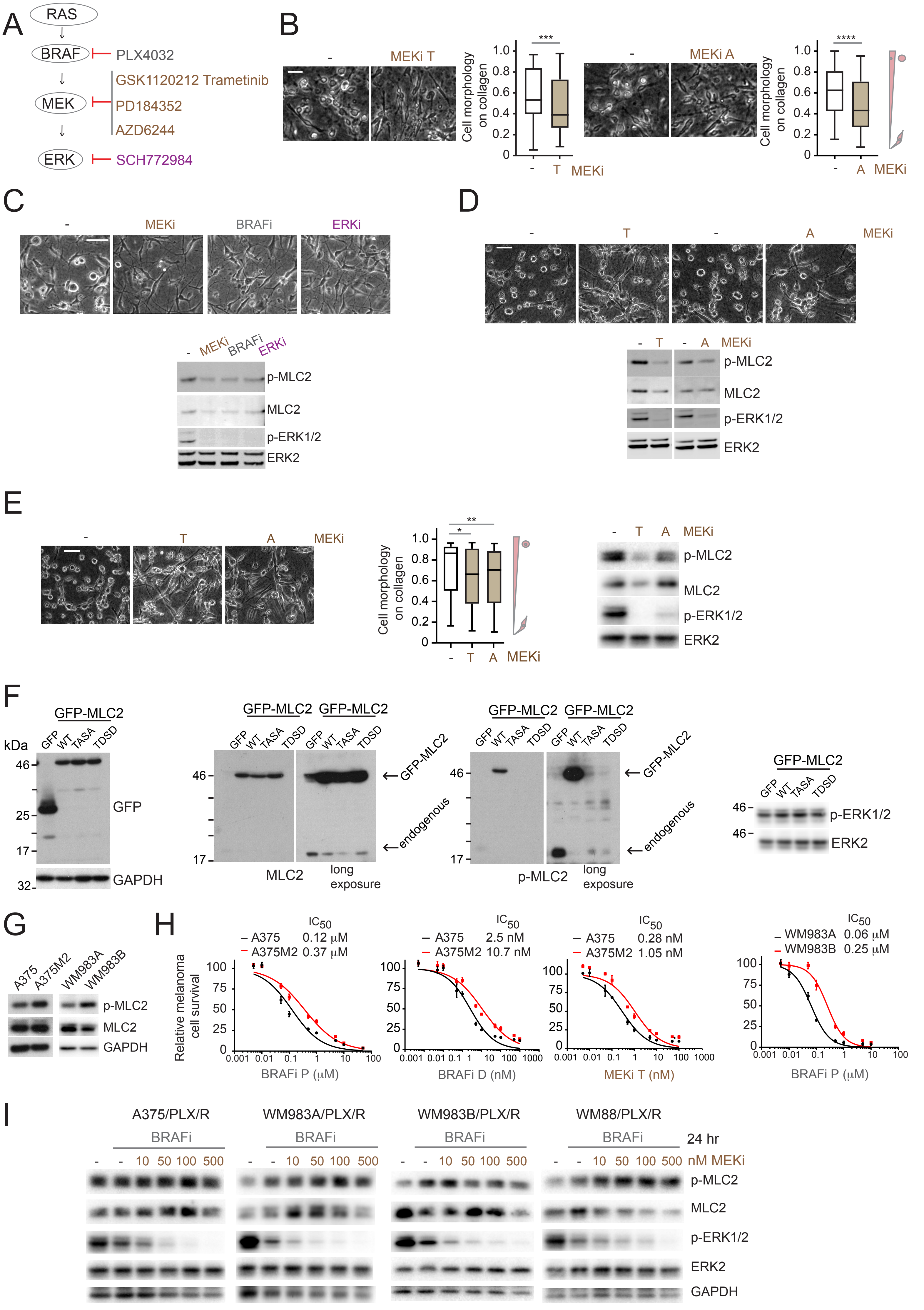
A
RAS
BRAF
PLX4032
GSK1120212 Trametinib
MEK
PD184352
AZD6244
ERK
SCH772984
B
MEKi T
MEKi A
Cell morphology on collagen
MEKi
C
MEKi
BRAFi
ERKi
p-MLC2
MLC2
p-ERK1/2
ERK2
D
MEKi
E
F
GFP-MLC2
GFP WT TASA TDSD
kDa
GFP
GAPDH
MLC2
long exposure
endogenous
G
A375 A375M2 WM983A WM983B
H
IC50
A375 0.12 µM
A375M2 0.37 µM
A375 2.5 nM
A375M2 10.7 nM
A375 0.28 nM
A375M2 1.05 nM
WM983A 0.06 µM
WM983B 0.25 µM
Relative melanoma cell survival
BRAFi P (µM)
BRAFi D (nM)
MEKi T (nM)
I
A375/PLX/R
WM983A/PLX/R
WM983B/PLX/R
WM88/PLX/R
BRAFi
24 hr
10 50 100 500 nM MEKi

Figure S1, related to Figure 1. MAPK regulates Myosin II activity in melanoma. (**A**) Summary of inhibitors used. (**B**) Morphology of 4599 cells on collagen I after 24 hr treatment (MEKi Trametinib, AZD6244) (n>100 cells). (**C-D**) Images of (**C**) $BRAF^{V600E}$/Pten null 690cl2 cells and (**D**) $NRAS^{Q61L}$ D04 on collagen I after 24 hr treatments (690 cl2 MEKi PD184352, BRAFi PLX4032, ERKi SCH772984; D04, MEKi Trametinib, AZD6244). p-MLC2 and p-ERK1/2 immunoblots are shown. (**E**) Images and cell morphology quantification of $NRAS^{Q61R}$ MM485 cells on collagen I after 24 hr treatment (n=150 cells). (**F**) Immunoblots of A375 cells overexpressing WT or mutant MLC2. (**G**) p-MLC2 and MLC2 immunoblots. (**H**) Dose-response curves and IC_{50} values to indicated inhibitors of cells treated for 3 days (BRAFi PLX4720, BRAFi Dabrafenib, MEKi Trametinib) (n=3). (**I**) Immunoblots of BRAFi-resistant cell lines after 24 hr treatment (BRAFi PLX4720, MEKi Trametinib).

Scale bars (B-E), 50 µm. Box plots (B, E) show median (center line); interquartile range (box); min-max values (whiskers). p values by Kruskal Wallis with Dunn's correction. *p<0.05, **p<0.01, ***p<0.001, ****p<0.0001.

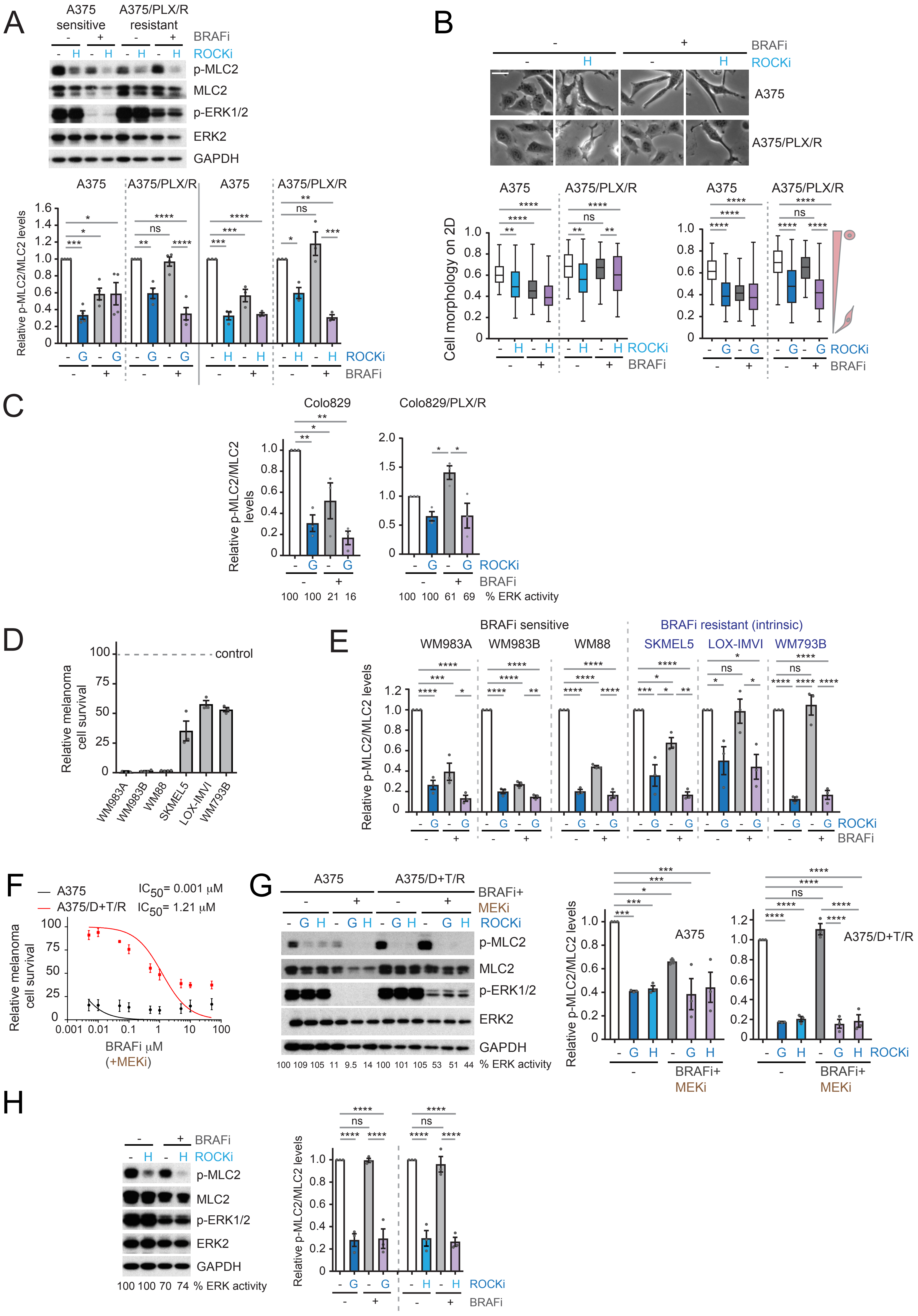
A
A375 sensitive
A375/PLX/R resistant
BRAFi
ROCKi
p-MLC2
MLC2
p-ERK1/2
ERK2
GAPDH
A375
A375/PLX/R
Relative p-MLC2/MLC2 levels
ROCKi
BRAFi
B
BRAFi
ROCKi
A375
A375/PLX/R
Cell morphology on 2D
ROCKi
BRAFi
C
Colo829
Colo829/PLX/R
Relative p-MLC2/MLC2 levels
ROCKi
BRAFi
100 100 21 16
100 100 61 69 % ERK activity
D
control
Relative melanoma cell survival
WM983A
WM983B
WM88
SKMEL5
LOX-IMVI
WM793B
E
BRAFi sensitive
BRAFi resistant (intrinsic)
WM983A
WM983B
WM88
SKMEL5
LOX-IMVI
WM793B
Relative p-MLC2/MLC2 levels
ROCKi
BRAFi
F
A375
A375/D+T/R
IC50= 0.001 μM
IC50= 1.21 μM
Relative melanoma cell survival
BRAFi μM
(+MEKi)
G
A375
A375/D+T/R
BRAFi+
MEKi
ROCKi
p-MLC2
MLC2
p-ERK1/2
ERK2
GAPDH
100 109 105 11 9.5 14 100 101 105 53 51 44 % ERK activity
Relative p-MLC2/MLC2 levels
A375
A375/D+T/R
ROCKi
BRAFi+
MEKi
H
BRAFi
ROCKi
p-MLC2
MLC2
p-ERK1/2
ERK2
GAPDH
100 100 70 74 % ERK activity
Relative p-MLC2/MLC2 levels
ROCKi
BRAFi

Figure S2, related to Figure 3. Therapy-resistant melanomas maintain high ROCK-driven Myosin II levels. (**A**) p-MLC2 immunoblots and quantification after 24 hr treatment with BRAFi PLX4720 and ROCKi GSK269962A (n=4) and H1152 (n=3). (**B**) Images and cell morphology quantification of A375 and A375/PLX/R cells after 24 hr treatment (BRAFi PLX4720; ROCKi GSK269962A, H1152) (n=100 cells pooled from 3 experiments). Scale bar, 50 µm. (**C**) p-MLC2 levels from immunoblotting after 24 hr treatment (BRAFi PLX4720, ROCKi GSK269962A) (n=3). (**D**) Survival after 7-14 day 5 µM PLX4720 treatment. (**E**) p-MLC2 levels after 24 hr treatment (BRAFi PLX4720, ROCKi GSK269962A) (n=3). (**F**) Survival of A375 and A375/D+T/R cells after 6 day treatment (BRAFi Dabrafenib and 10 nM MEKi Trametinib) (n=3). (**G**) p-MLC2 and p-ERK1/2 immunoblots and quantification after 24 hr treatment (BRAFi Dabrafenib, MEKi Trametinib; ROCKi GSK269962A and H1152) (n=3). (**H**) p-MLC2 and p-ERK1/2 immunoblots of Patient #35 cells after 24 hr treatment (BRAFi PLX4720; ROCKi GSK269962A, H1152) (n=3).

Graphs show mean ± SEM and individual data points (circle) except box plot in B (median (center line); interquartile range (box); min-max values (whiskers)). p values by ANOVA with Tukey's correction (A, C, E, G, H), Kruskal-Wallis with Dunn's correction (B). *$p<0.05$, **$p<0.01$, ***$p<0.001$, ****$p<0.0001$, ns not significant.

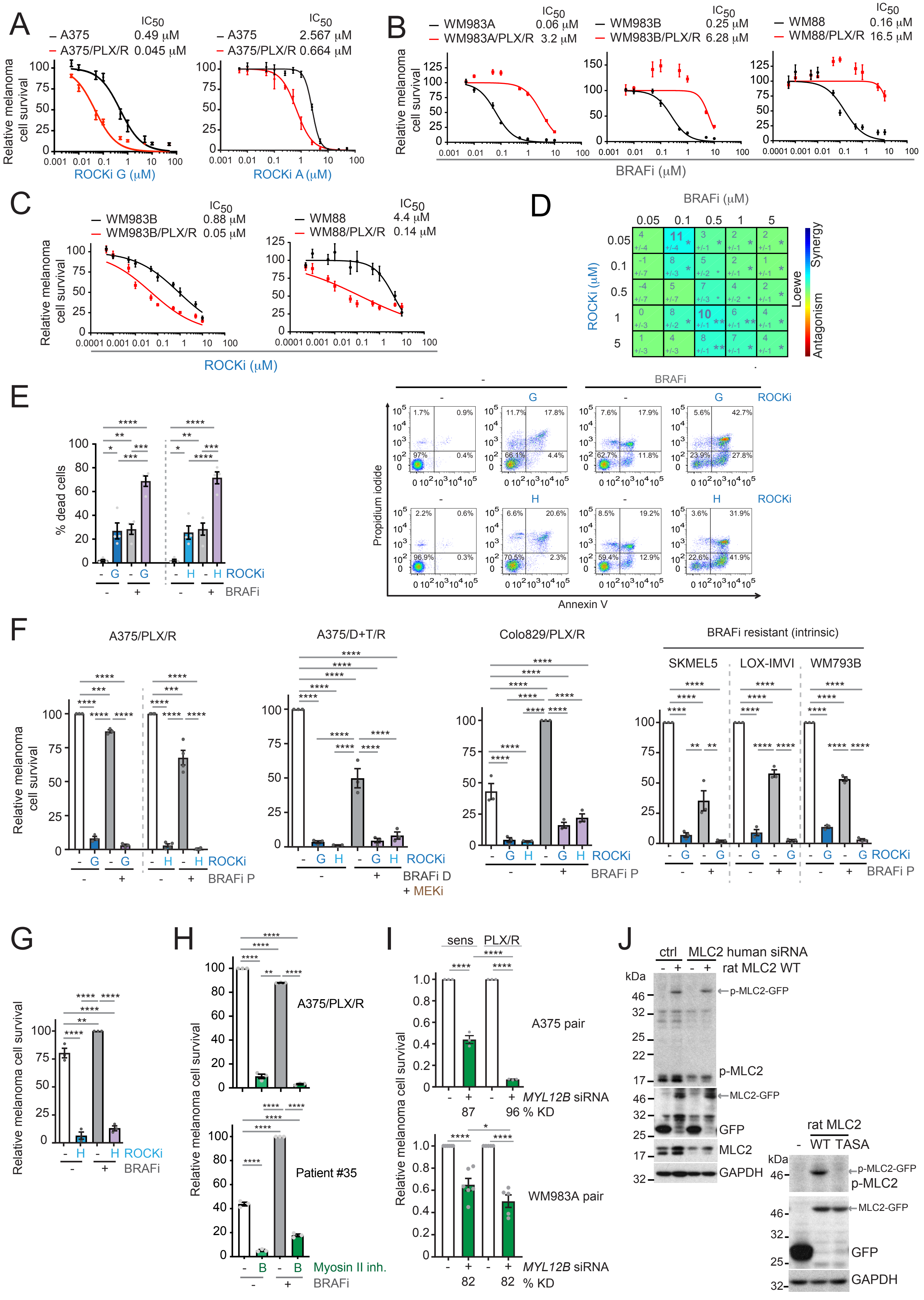

A
B
C
D
E
F
G
H
I
J
A375
A375/PLX/R
IC50
0.49 μM
0.045 μM
2.567 μM
0.664 μM
Relative melanoma cell survival
ROCKi G (μM)
ROCKi A (μM)
WM983A
WM983A/PLX/R
0.06 μM
3.2 μM
WM983B
WM983B/PLX/R
0.25 μM
6.28 μM
WM88
WM88/PLX/R
0.16 μM
16.5 μM
BRAFi (μM)
0.88 μM
0.05 μM
4.4 μM
0.14 μM
ROCKi (μM)
Synergy
Loewe
Antagonism
% dead cells
ROCKi
BRAFi
Propidium iodide
Annexin V
A375/D+T/R
Colo829/PLX/R
BRAFi resistant (intrinsic)
SKMEL5
LOX-IMVI
WM793B
BRAFi P
BRAFi D
+ MEKi
Myosin II inh.
Patient #35
sens
PLX/R
A375 pair
WM983A pair
MYL12B siRNA
% KD
ctrl
MLC2 human siRNA
rat MLC2 WT
kDa
p-MLC2-GFP
p-MLC2
MLC2-GFP
GFP
MLC2
GAPDH
rat MLC2
WT
TASA

Figure S3, related to Figure 3. Survival of targeted therapy-resistant melanomas is dependent on ROCK-driven Myosin II activity. (**A**) Survival after 3 day treatment (ROCKi GSK269962A, AT13148) (n=3). In GSK269962A experiments, resistant cells were cultured without PLX4720. (**B-C**) Survival after 3-5 day treatment (BRAFi PLX4720, ROCKi GSK269962A) (n=3-5). (**D**) Average synergy scores of ROCKi GSK269962A and BRAFi PLX4720 treatments of A375 cells (n=4) (*p<0.05; **p<0.001). (**E**) % of dead A375 cells by annexin V/PI staining after 3 day treatment (BRAFi PLX4720, ROCKi GSK269962A and H1152) (n=4). Representative FACS plots are shown. (**F**) Survival of A375/PLX/R (n=3, GSK269962A; n=4, H1152); A375/D+T/R (n=3); Colo829/PLX/R (n=3); and BRAFi-resistant cell lines (n=3) after treatments for 5 days (A375/PLX/R), 10 days (A375/D+T/R, Colo829/PLX/R) and 7-14 days (SKMEL5, LOX-IMVI, WM793B). BRAFi PLX4720 for A375/PLX/R, Colo829/PLX/R and intrinsic panel; BRAFi Dabrafenib and MEKi trametinib for A375/D+T/R; ROCKi GSK269962A, H1152. (**G**) Survival of Patient #35 cells after 10 day treatment (ROCKi H1152, BRAFi PLX4720) (n=3). (**H**) Survival after 5-10 day treatment (BRAFi PLX4720, Blebbistatin) (n=3). Data for cells under PLX4720 (+/- blebbistatin) is also shown in Figure 3L. (**I**) Survival 8 days after *MYL12B* KD (n=3 A375 pair; n=6 WM983A pair). Resistant cells were grown in the presence of 1 μM PLX4720 for the whole experiment. mRNA KD (% decrease vs control) by qRT-PCR is shown. (**J**) GFP, p-MLC2 immunoblots of A375/PLX/R cells after MLC2 knockdown and rescue with rat MLC2 (WT or TASA mutant). Graphs show mean ± SEM and individual data points (circle). p values by ANOVA with Tukey's correction (E-I), *p<0.05, **p<0.01, ***p<0.001, ****p<0.0001, ns not significant.

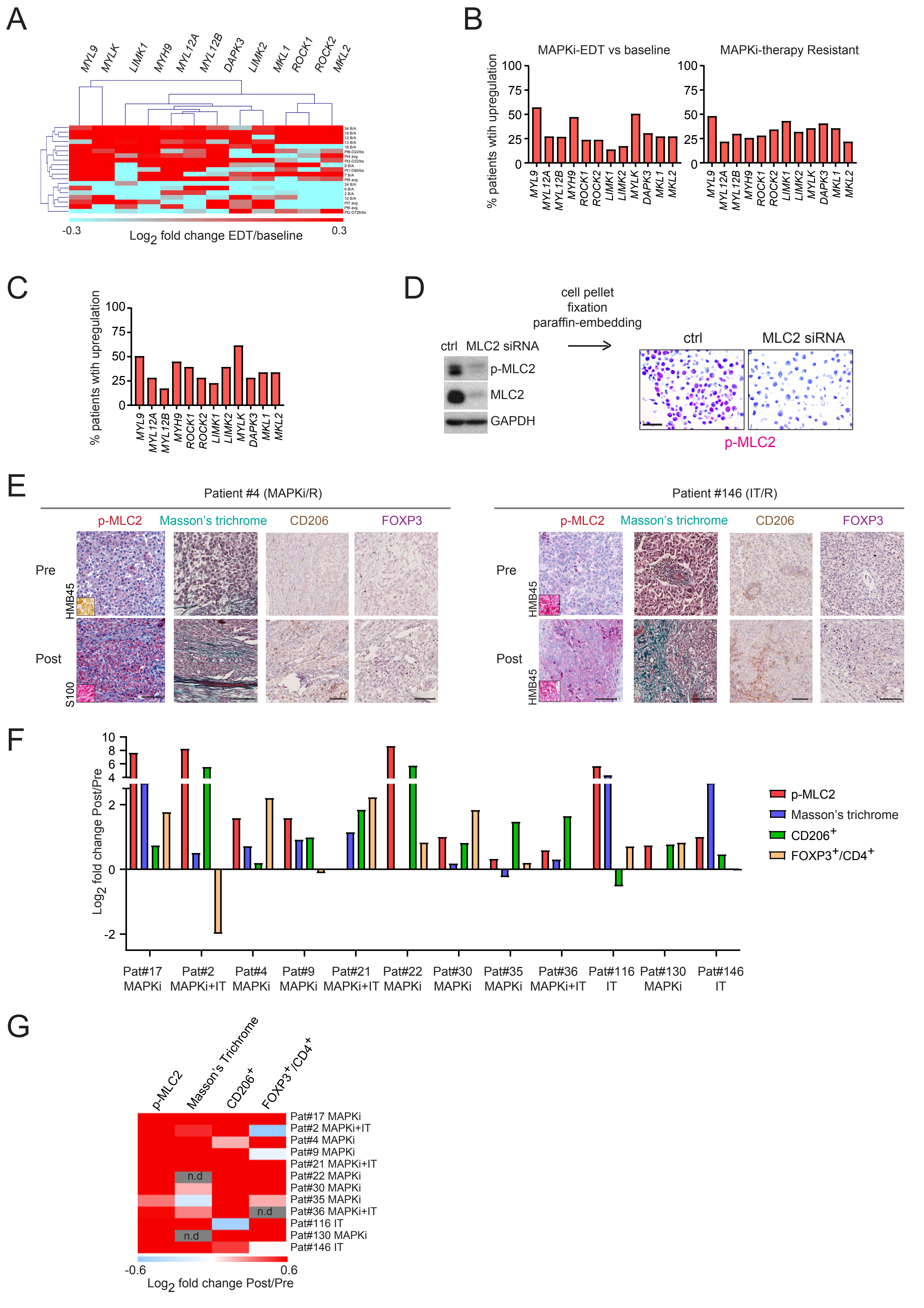

A
MYL9
MYLK
LIMK1
MYH9
MYL12A
MYL12B
DAPK3
LIMK2
MKL1
ROCK1
ROCK2
MKL2
-0.3
Log2 fold change EDT/baseline
0.3
B
MAPKi-EDT vs baseline
MAPKi-therapy Resistant
% patients wtih upregulation
C
% patients wtih upregulation
D
ctrl
MLC2 siRNA
p-MLC2
MLC2
GAPDH
cell pellet
fixation
paraffin-embedding
p-MLC2
E
Patient #4 (MAPKi/R)
Patient #146 (IT/R)
p-MLC2
Masson's trichrome
CD206
FOXP3
Pre
Post
HMB45
S100
F
Log2 fold change Post/Pre
p-MLC2
Masson's trichrome
CD206+
FOXP3+/CD4+
Pat#17 MAPKi
Pat#2 MAPKi+IT
Pat#4 MAPKi
Pat#9 MAPKi
Pat#21 MAPKi+IT
Pat#22 MAPKi
Pat#30 MAPKi
Pat#35 MAPKi
Pat#36 MAPKi+IT
Pat#116 IT
Pat#130 MAPKi
Pat#146 IT
G
p-MLC2
Masson's Trichrome
CD206+
FOXP3+/CD4+
n.d
-0.6
0.6
Log2 fold change Post/Pre

Figure S4, related to Figure 4. High Myosin II levels identify therapy-resistant melanomas in human samples. (**A**) Heatmap of fold change in expression of ROCK-Myosin II pathway genes in paired MAPKi-early during treatment (EDT) vs baseline patient samples from (Kwong et al., 2015; Song et al., 2017). (**B**) % of patients with upregulation (>1.3fold) of indicated genes in EDT samples vs baseline (left graph) (Kakavand et al., 2017; Kwong et al., 2015; Long et al., 2014; Rizos et al., 2014; Song et al., 2017); and in Resistant samples vs baseline (right graph) (Hugo et al., 2015; Kakavand et al., 2017; Kwong et al., 2015; Long et al., 2014; Rizos et al., 2014; Sun et al., 2014; Wagle et al., 2014). (**C**) % of patients with upregulation (>1.3fold) of indicated genes in samples on anti-PD-1 vs baseline from (Riaz et al., 2017). (**D**) Validation of p-MLC2 antibody in paraffin-embedded cell pellets of A375/PLX/R cells transfected with siRNA against MLC2. Non-targeting siRNA was used as control. Left, p-MLC2 and MLC2 immunoblots. Right, photomicrographs of p-MLC2 staining in cell pellet sections. Scale bar, 100 μm. (**E**) Photomicrographs of p-MLC2, Masson's Trichrome, CD206 and FOXP3 staining in representative paired samples from patients before and after therapy. Insets show IHC staining with melanoma markers HMB45/Melan-A or S100. Scale bars, 100 μm. (**F**) Fold change in levels of p-MLC2, Masson's, $CD206^{+}$ and ratio $FOXP3^{+}/CD4^{+}$ in 12 post- vs pre-treatment samples (including those in S4E and Figures 4H-K). (**G**) Heatmap of fold change in levels of indicated markers in post vs pre-treatment samples from S4F. n.d., not determined.

Table S5, related to Figure 4. List of gene signatures from ssGSEA.

Gene signature	Process
GOTZMANN_EPITHELIAL_TO_MESENCHYMAL_TRANSITION_UP	EMT/metastasis
ANASTASSIOU_CANCER_MESENCHYMAL_TRANSITION_SIGNATURE	EMT/metastasis
HALLMARK_EPITHELIAL_MESENCHYMAL_TRANSITION	EMT/metastasis
WU_CELL_MIGRATION	EMT/metastasis
LU_TUMOR_ANGIOGENESIS_UP	angiogenesis
LU_TUMOR_ENDOTHELIAL_MARKERS_UP	angiogenesis
LU_TUMOR_VASCULATURE_UP	angiogenesis
CLASPER_LYMPHATIC_VESSELS_DURING_METASTASIS_UP	angiogenesis
MANALO_HYPOXIA_UP	hypoxia
QI_HYPOXIA	hypoxia
ROY_WOUND_BLOOD_VESSEL_UP	wound healing
KEGG_TGF_BETA_SIGNALING_PATHWAY	TGFβ
COULOUARN_TEMPORAL_TGFB1_SIGNATURE_UP	TGFβ
AZARE_NEOPLASTIC_TRANSFORMATION_BY_STAT3_UP	STAT3
HALLMARK_IL6_JAK_STAT3_SIGNALING	STAT3
DAUER_STAT3_TARGETS_UP	STAT3
HALLMARK_INFLAMMATORY_RESPONSE	NF-κB
SCHOEN_NFKB_SIGNALING	NF-κB
HINATA_NFKB_TARGETS_FIBROBLAST_UP	NF-κB
HALLMARK_TNFA_SIGNALING_VIA_NFKB	NF-κB
CORDENONSI_YAP_CONSERVED_SIGNATURE	YAP

Table S6, related to Figure 4. Summary of clinical data of patient samples used for stainings.

**Patient#**	**Mutations**	**Treatment**	**Pre-Treatment Block Tissue**	**Post-Treatment Block tissue**
#17	$BRAF^{V600E}$ $NRAS^{Q61R}$	dabrafenib+trametinib	left axillary dissection	left axillary nodal mass
#4	$BRAF^{V600E}$	vemurafenib	nodule left breast	excision of cutaneous lesion
#2	$BRAF^{V600E}$	vemurafenib, ipilimumab	right chest	skin lesion to chest wall
#35	$BRAF^{V600E}$ $NRAS^{Q61R}$	vemurafenib	dorsum, right hand	right axillary lymph node
#9	$BRAF^{V600E}$	vemurafenib	left calf	left groin lymph node dissection
#21	$BRAF^{V600E}$	vemurafenib,+3 day pembrolizumab	right lower leg	right lower leg
#22	$BRAF^{V600E}$	vemurafenib	right dorsum of foot	right upper thigh tumour
#30	$BRAF^{V600E}$	vemurafenib dabtram	left ear lobe	left postauricular region
#36	$BRAF^{V600E}$	vemurafenib, ipilimumab	left neck	right deltoid melanoma deposit
#116	$NRAS^{Q61R}$	ipilimumab	right inguinal node dissection	proximal small bowel resection.
#130	$BRAF^{V600E}$	vemurafenib, dabrafenib+trametinib	excised from right flank	right frontal craniotomy
#146	BRAF Wildtype	ipilimumab	right Ileoinguinal node dissection	right external oblique. Right internal oblique mass

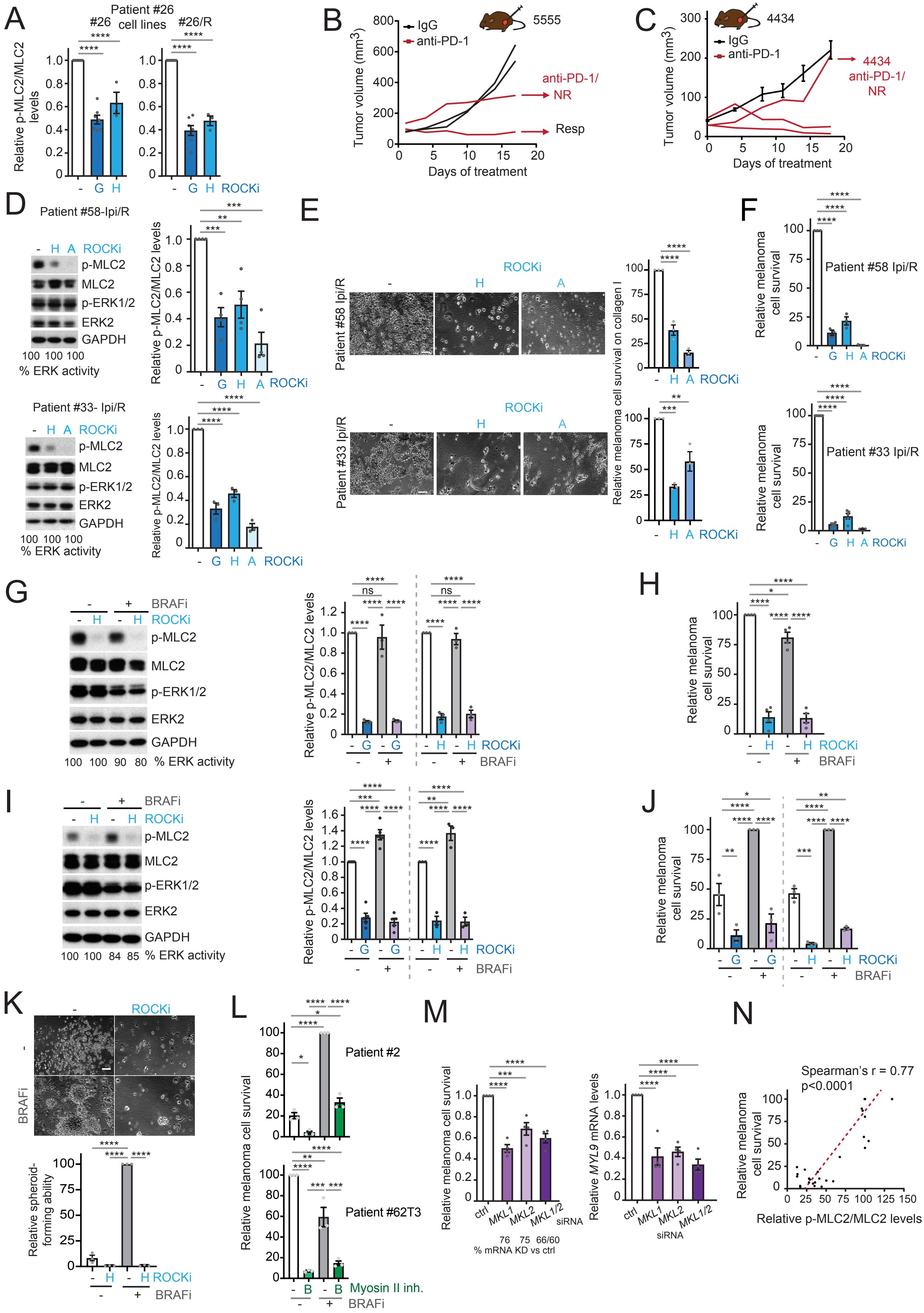
A
Patient #26 cell lines
#26
#26/R
Relative p-MLC2/MLC2 levels
- G H ROCKi
B
5555
IgG
anti-PD-1
Tumor volume (mm3)
anti-PD-1/ NR
Resp
Days of treatment
C
4434
IgG
anti-PD-1
4434 anti-PD-1/ NR
Tumor volume (mm3)
Days of treatment
D
Patient #58-Ipi/R
- H A ROCKi
p-MLC2
MLC2
p-ERK1/2
ERK2
GAPDH
100 100 100
% ERK activity
Relative p-MLC2/MLC2 levels
- G H A ROCKi
Patient #33- Ipi/R
- H A ROCKi
p-MLC2
MLC2
p-ERK1/2
ERK2
GAPDH
100 100 100
% ERK activity
E
ROCKi
Patient #58 Ipi/R
- H A
Patient #33 Ipi/R
Relative melanoma cell survival on collagen I
- H A ROCKi
F
Patient #58 Ipi/R
Patient #33 Ipi/R
Relative melanoma cell survival
- G H A ROCKi
G
BRAFi
- H - H ROCKi
p-MLC2
MLC2
p-ERK1/2
ERK2
GAPDH
100 100 90 80 % ERK activity
Relative p-MLC2/MLC2 levels
- G - G - H - H ROCKi
BRAFi
H
Relative melanoma cell survival
- H - H ROCKi
BRAFi
I
BRAFi
- H - H ROCKi
p-MLC2
MLC2
p-ERK1/2
ERK2
GAPDH
100 100 84 85 % ERK activity
Relative p-MLC2/MLC2 levels
- G - G - H - H ROCKi
BRAFi
J
Relative melanoma cell survival
- G - G - H - H ROCKi
BRAFi
K
- ROCKi
BRAFi
Relative spheroid-forming ability
- H - H ROCKi
BRAFi
L
Patient #2
Patient #62T3
Relative melanoma cell survival
- B - B Myosin II inh.
BRAFi
M
Relative melanoma cell survival
ctrl MKL1 MKL2 MKL1/2 siRNA
76 75 66/60
% mRNA KD vs ctrl
Relative MYL9 mRNA levels
ctrl MKL1 MKL2 MKL1/2
siRNA
N
Spearman's r = 0.77
p<0.0001
Relative melanoma cell survival
Relative p-MLC2/MLC2 levels

Figure S5, related to Figure 5. ROCK-driven Myosin II activity in immunotherapy-resistant melanoma. (**A**) p-MLC2 levels after 24 hr treatments (n=7 GSK269962A (G); n=3 H1152 (H)). Immunoblots are in Figure 5A. (**B**) Growth of $Braf^{V600E}$ mouse 5555 allografts after anti-PD-1 or IgG isotype treatment. Indicated cell lines were established from anti-PD-1/non-responder (NR) and anti-PD-1/responder (Resp) 5555 tumors. (**C**) Growth of $Braf^{V600E}$ mouse 4434 allografts after treatment. Indicated cell lines were established from anti-PD-1/NR tumors. (**D**) p-MLC2 immunoblots and quantification of Patient #58 cells (n=4) and Patient #33 (n=3) cells after 24 hr ROCKi treatment (GSK269962A (G); H1152 (H); AT13148 (A)). (**E**) Images and quantification of cell survival on collagen I for 9-14 days (n=3). Scale bars, 100 µm. (**F**) Survival after 9-14 day treatment (n=5 #33; n=3 #58). (**G**) p-MLC2 immunoblots and quantification of Patient #62T3 cells after 24 hr ROCKi treatment (ROCKi H1152 and GSK269962A, BRAFi PLX4720) (n=3). (**H**) Survival of Patient #62T3 cells after 10 day treatment (ROCKi H1152, BRAFi PLX4720) (n=4). (**I**) p-MLC2 immunoblots and quantification of Patient #2 cells after 24 hr treatment (ROCKi H1152 and GSK269962A; BRAFi PLX4720) (n=5 GSK269962A; n=3 H1152). (**J**) Survival of Patient #2 cells after 6 day treatment (ROCKi GSK269962A and H1152; BRAFi PLX4720) (n=3). (**K**) Spheroid-forming ability of Patient #2 cells on collagen I after 16 day treatment (ROCKi H1152, BRAFi PLX4720) (n=3). Scale bar, 100 µm. (**L**) Survival after 10 day treatment (Myosin II inhibitor Blebbistatin, BRAFi PLX4720) (n=3). Data for cells under PLX4720 (+/- blebbistatin) is also shown in main Figure 5K. (**M**) Survival (left) and MLC2 (*MYL9*) expression (right) in Patient #2 cells after MRTF (*MKL*) depletion (n=4). Expression and % of knockdown by qRT-PCR is shown. (**N**) Scatter plot of average relative p-MLC2/MLC2 levels and average relative survival in resistant cells. Red line indicates Deming linear regression analysis. Correlation (Spearman's r) between p-MLC2 and survival is also shown.

Graphs show mean ± SEM and individual data points (circle). p values by ANOVA with Dunnett's (A, D-F, M) or Tukey's correction (G-L) and Spearman's r (N).

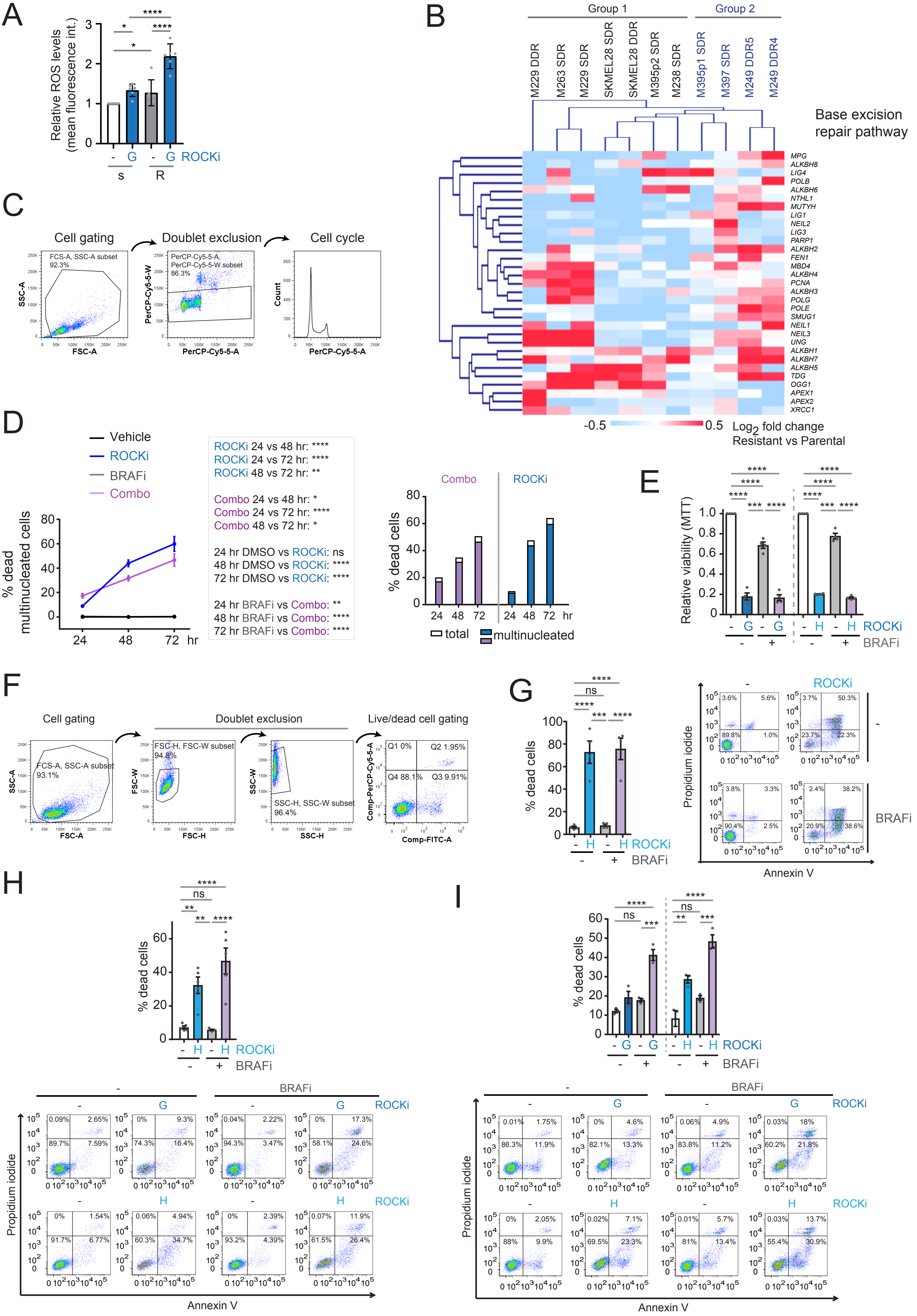
A
Relative ROS levels (mean fluorescence int.)
- G - G ROCKi
S R
B
Group 1
Group 2
M229 DDR
M263 SDR
M229 SDR
SKMEL28 SDR
SKMEL28 DDR
M395p2 SDR
M238 SDR
M395p1 SDR
M397 SDR
M249 DDR5
M249 DDR4
Base excision repair pathway
MPG
ALKBH8
LIG4
POLB
ALKBH6
NTHL1
MUTYH
LIG1
NEIL2
LIG3
PARP1
ALKBH2
FEN1
MBD4
ALKBH4
PCNA
ALKBH3
POLG
POLE
SMUG1
NEIL1
NEIL3
UNG
ALKBH1
ALKBH7
ALKBH5
TDG
OGG1
APEX1
APEX2
XRCC1
-0.5 0.5 Log2 fold change
Resistant vs Parental
C
Cell gating
Doublet exclusion
Cell cycle
FCS-A, SSC-A subset 92.3%
PerCP-Cy5-5-A, PerCP-Cy5-5-W subset 86.3%
D
Vehicle
ROCKi
BRAFi
Combo
% dead multinucleated cells
ROCKi 24 vs 48 hr: ****
ROCKi 24 vs 72 hr: ****
ROCKi 48 vs 72 hr: **
Combo 24 vs 48 hr: *
Combo 24 vs 72 hr: ****
Combo 48 vs 72 hr: *
24 hr DMSO vs ROCKi: ns
48 hr DMSO vs ROCKi: ****
72 hr DMSO vs ROCKi: ****
24 hr BRAFi vs Combo: **
48 hr BRAFi vs Combo: ****
72 hr BRAFi vs Combo: ****
% dead cells
total
multinucleated
E
Relative viability (MTT)
- G - G - H - H ROCKi
- + - + BRAFi
F
Cell gating
Doublet exclusion
Live/dead cell gating
FCS-A, SSC-A subset 93.1%
FSC-H, FSC-W subset 94.8%
SSC-H, SSC-W subset 96.4%
Q1 0%
Q2 1.95%
Q4 88.1%
Q3 9.91%
G
% dead cells
- H - H ROCKi
- + BRAFi
Propidium iodide
Annexin V
H
I

Figure S6, related to Figure 6. ROCK-Myosin II inhibition induces lethal reactive oxygen species (ROS), DNA damage and cell cycle arrest. (**A**) ROS levels in WM983A (s) and WM983A/PLX/R (R) cells after 48 hr ROCKi GSK269962A treatment (n= 6). (**B**) Heatmap of fold change in expression of genes in the base excision repair pathway in resistant vs parental cell lines from (Song et al., 2017). (**C**) Gating strategy for cell cycle experiments. (**D**) Left, % of dead multinucleated cells after 72 hr treatment (ROCKi GSK269962A, BRAFi PLX4720) of A375/PLX/R cells and imaged with time-lapse video-microscopy (n=6 movies/treatment pooled from 2 independent experiments). Right, average % of total dead cells and dead multinucleated cells. (**E**) Viability of A375/PLX/R cells by MTT assay after 72 hr treatment (ROCKi GSK269962A and H1152; BRAFi PLX4720) (n=4). (**F**) Gating strategy for annexin V/propidium iodide (PI) experiments. (**G-I**) % of dead cells measured by annexin V/PI staining after 72 hr treatment (ROCKi GSK269962A and H1152; BRAFi PLX4720) of: A375/PLXR (n=4) (**G**); Patient #2 (n=5) (**H**); Patient #35 (n=3) (**I**). Representative FACS plots are shown. Graphs show mean ± SEM and individual data points (circle). p values were estimated by one-way ANOVA with Tukey's correction (E, G-I), Benjamini, Krieger and Yekutieli correction (A) and two-way ANOVA with Tukey's correction (D), *p<0.05, **p<0.01, ***p<0.001, ****p<0.0001, ns not significant.

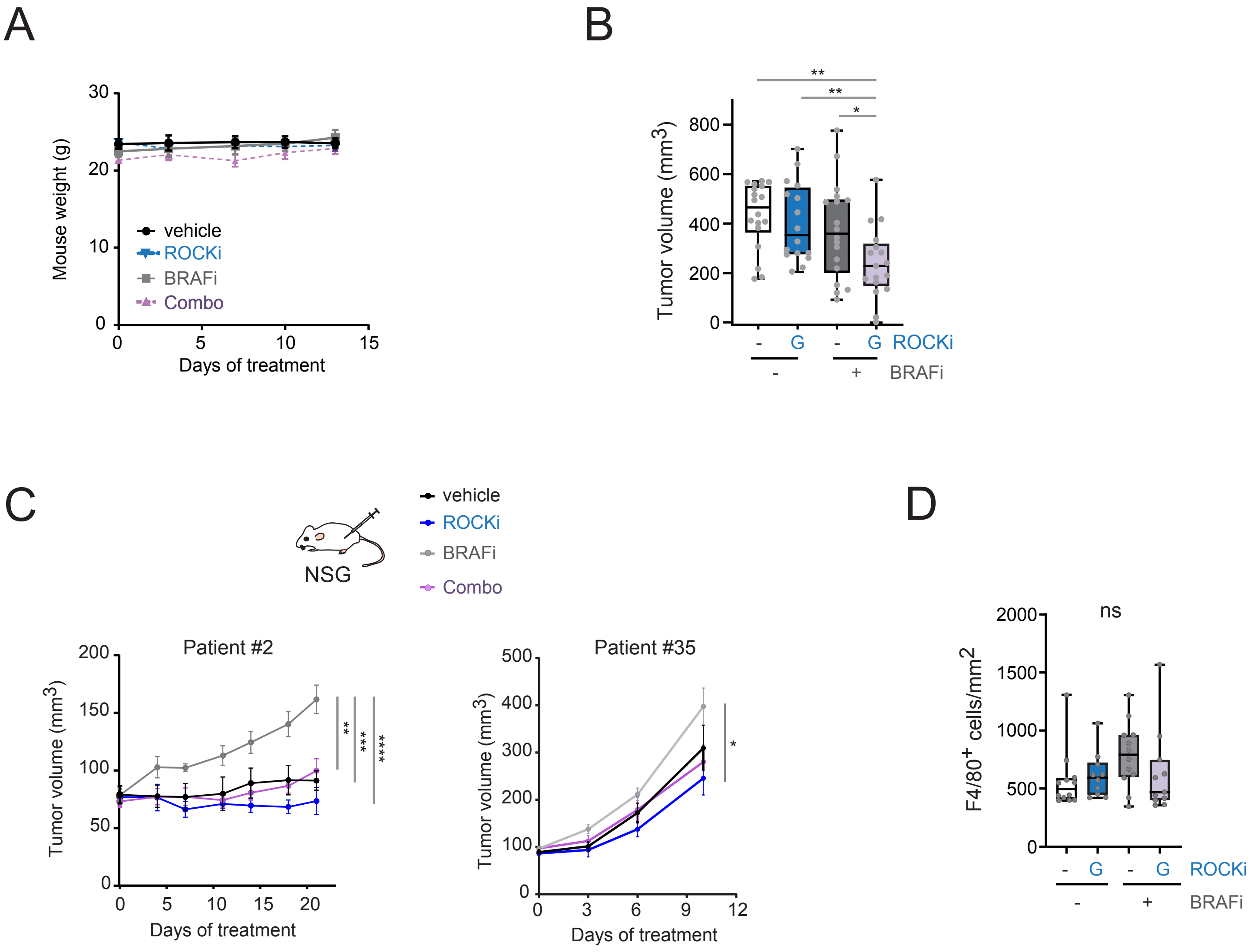

A
Mouse weight (g)
Days of treatment
vehicle
ROCKi
BRAFi
Combo
B
Tumor volume (mm³)
** ** *
- G - G ROCKi
- + BRAFi
C
NSG
vehicle
ROCKi
BRAFi
Combo
Patient #2
Tumor volume (mm³)
Days of treatment
** *** ****
Patient #35
Tumor volume (mm³)
Days of treatment
*
D
ns
F4/80+ cells/mm2
- G - G ROCKi
- + BRAFi

Figure S7, related to Figure 7. Combining ROCK inhibitors with BRAF inhibitors *in vivo*. (**A**) Weight of nude mice xenografted with A375/PLX/R after treatment. (**B**) Tumor volume at endpoint of A375/PLX/R xenografts. Pooled data from 3 independent experiments are shown. (**C**) Growth of (left) Patient #2 and (right) Patient #62T3 xenografts in NSG mice after treatments. (**D**) Quantification of F4/80$^+$ cells in A375/PLX/R xenografts.
A-D: ROCKi GSK269962A, BRAFi PLX4720.
A, C show mean ± SEM. B, D show box plots with median (center line); interquartile range (box); min-max (whiskers); and individual mice (circles). p values by ANOVA with Benjamini, Krieger and Yekutieli (B, D), Dunnett's (C, right) or Tukey's (C, left) correction.

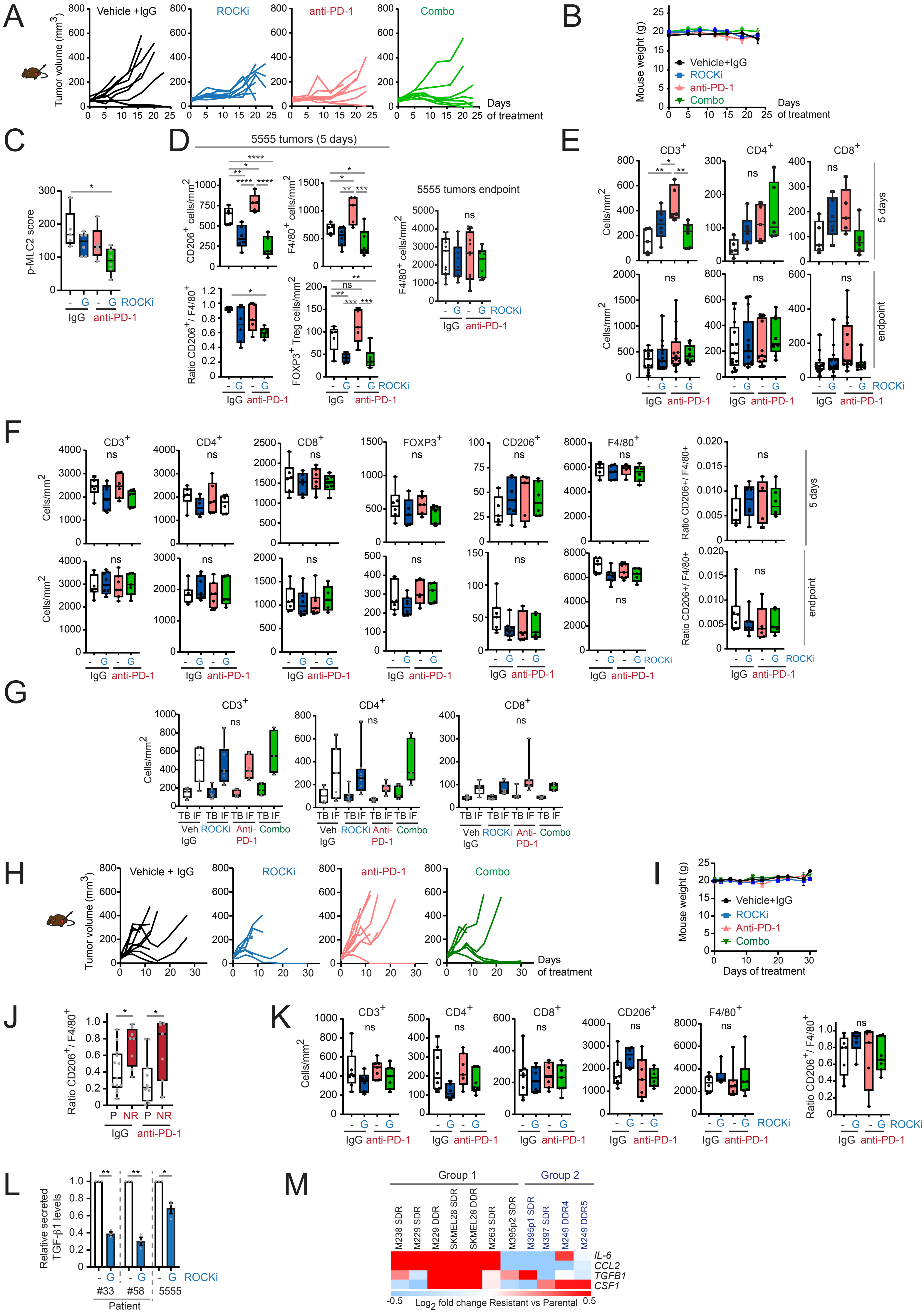
A
Vehicle +IgG
ROCKi
anti-PD-1
Combo
Tumor volume (mm3)
Days of treatment
B
Mouse weight (g)
Vehicle+IgG
ROCKi
anti-PD-1
Combo
Days of treatment
C
p-MLC2 score
IgG
anti-PD-1
ROCKi
D
5555 tumors (5 days)
CD206+ cells/mm2
F4/80+ cells/mm2
Ratio CD206+/ F4/80+
FOXP3+ Treg cells/mm2
5555 tumors endpoint
F4/80+ cells/mm2
E
CD3+
CD4+
CD8+
Cells/mm2
5 days
endpoint
F
CD3+
CD4+
CD8+
FOXP3+
CD206+
F4/80+
Ratio CD206+/ F4/80+
Cells/mm2
5 days
endpoint
G
CD3+
CD4+
CD8+
Cells/mm2
TB IF
Veh IgG
ROCKi
Anti-PD-1
Combo
H
Vehicle + IgG
ROCKi
anti-PD-1
Combo
Tumor volume (mm3)
Days of treatment
I
Mouse weight (g)
Vehicle+IgG
ROCKi
Anti-PD-1
Combo
Days of treatment
J
Ratio CD206+/ F4/80+
P NR P NR
IgG anti-PD-1
K
CD3+
CD4+
CD8+
CD206+
F4/80+
Cells/mm2
Ratio CD206+/ F4/80+
L
Relative secreted TGF-β1 levels
#33
#58
5555
Patient
ROCKi
M
Group 1
Group 2
M238 SDR
M229 SDR
M229 DDR
SKMEL28 SDR
SKMEL28 DDR
M263 SDR
M395p2 SDR
M395p1 SDR
M397 SDR
M249 DDR4
M249 DDR5
IL-6
CCL2
TGFB1
CSF1
-0.5
Log2 fold change Resistant vs Parental
0.5

Figure S8, related to Figure 8. ROCK-Myosin II inhibition improves efficacy of immune checkpoint inhibitors. (**A**) Growth of $Braf^{V600E}$ mouse 5555 allografts in C57BL/6J after treatment. (**B**) Mouse weights from A. (**C**) Quantification of p-MLC2 in 5555 tumors from A at 5 days of treatment. (**D**) Quantification of $CD206^+$, $FOXP3^+$ and $F4/80^+$ cells in 5555 tumors from A at 5 days of treatment (left); and $F4/80^+$ at endpoint (right). (**E**) Quantification of $CD3^+$, $CD4^+$ and $CD8^+$ cells in 5555 tumors from A. (**F**) Quantification of $CD3^+$, $CD4^+$, $CD8^+$, $FOXP3^+$, $CD206^+$ and $F4/80^+$ cells in spleens from 5555-bearing mice from A. (**G**) Regional analysis of immune populations in tumor body (TB) and invasive front (IF) of 5555 tumors from A at endpoint. (**H**) Growth of 5555-anti-PD-1/NR allografts after treatments. (**I**) Mouse weights from H. (**J**) Ratio $CD206^+/F4/80^+$ in parental 5555 (P) and 5555-anti-PD-1/NR (NR) tumors under IgG or anti-PD-1 treatment. (**K**) Quantification of $CD3^+$, $CD4^+$, $CD8^+$, $CD206^+$, $F4/80^+$ cells and ratio $CD206^+/F4/80^+$ in 5555-anti-PD-1/NR tumors from H. (**L**) TGF-β1 protein levels by ELISA after 48 hr treatment. (**M**) Heatmap of fold change in expression of indicated immunosuppressive cytokines in MAPKi-resistant vs parental cell lines from (Song et al., 2017).

A-I, K-L: ROCKi GSK269962A.

B, I, L show mean ± SEM. Box plots show median (center line); interquartile range (box); min-max (whiskers); and individual mice (circles). 5555 endpoint tumors in D-E include pooled data from 2 independent experiments.

P-values by ANOVA with Tukey's (G) or Benjamini, Krieger and Yekutieli correction (C-F, J-K), t-test with Welch's correction (L).

For G, statistical analysis of IF between groups: not significant. Statistical analysis $CD3^+$ TB vs IF: veh ($p=0.0063$), ROCKi ($p=0.0016$), anti-PD-1 ($p=0.01$), combo ($p=0.0009$). Statistical analysis $CD4^+$ TB vs IF: combo ($p=0.04$), not significant for the other groups. Statistical analysis $CD8^+$ TB vs IF: anti-PD-1 ($p=0.0037$), not significant for the other groups. *$p<0.05$, **$p<0.01$, ***$p<0.001$, ****$p<0.0001$, ns not significant.

Table S7, related to STAR Methods. List of RNAi sequences.

Target	RNAi sequences
Non-targeting Control siRNA	ON-TARGET (OT) plus non-targeting siRNA #1:<br>5'-UGGUUUACAUGUCGACUAA-3'
MKL1	OT SmartPool:<br>OT#5: 5'-GAUCGGAGCUGGUCAGGAU-3'<br>OT#6: 5'-GACAGAGGACUAUCUCAAA-3'<br>OT#7: 5'-GAACUAUCCCAAAGUAGCA-3'<br>OT#8: 5'-AAACUGAGCUGAUUGAGCG-3'
MKL2	OT SmartPool:<br>OT#5: 5'-GGACUAACGUCACAGAUUU -3'<br>OT#6: 5'- CAGCAGCCCUUUAUCAAUA -3'<br>OT#7: 5'-UCAGGGCGAUUUCUCAUUU-3'<br>OT#8: 5'-GAACCUAUGGGCAGUUUAU-3'
MYL9	OT#5: 5'-CCAAGGAUAAAGACGACUA-3'
MYH9	OT SmartPool:<br>OT#5: 5'-GUAUCAAUGUGACCGAUUU-3'<br>OT#6: 5'-CAAAGGAGCCCUGGCGUUA-3'<br>OT#7: 5'-GGAGGAACGCCGAGCAGUA-3'<br>OT#8: 5'-CGAAGCGGGUGAAAGCAAA-3'
MYL12B	OT SmartPool:<br>OT#9: 5'-CCACUUAGCACUUGUAUAA-3'<br>OT#10: 5'-GGGUGUAAAUUGUAUUGAA-3'<br>OT#11: 5'-CCUCAUAGAACCUGUUGCA-3'<br>OT#12: 5'-UGUAUUUAUUCCAGACCUU-3'
ROCK1	OT#8: 5'-CCAGGAAGGUAUAUGCUAU-3'
ROCK2	OT#8: 5'-GAAACUAAUAGGACACUAA-3'
