# Supplementary material for: Myosin II Reactivation and Cytoskeletal Remodeling as a Hallmark and a Vulnerability in Melanoma Therapy Resistance
Source: Cancer Cell. 2020 Jan 13;37(1):85–103.e9. doi: 10.1016/j.ccell.2019.12.003 (PMC6958528; doi:10.1016/j.ccell.2019.12.003)
Supplement: Document S2. Article plus Supplemental Information [file mmc6.pdf]

Article

Cancer Cell

Myosin II Reactivation and Cytoskeletal Remodeling as a Hallmark and a Vulnerability in Melanoma Therapy Resistance

Graphical Abstract

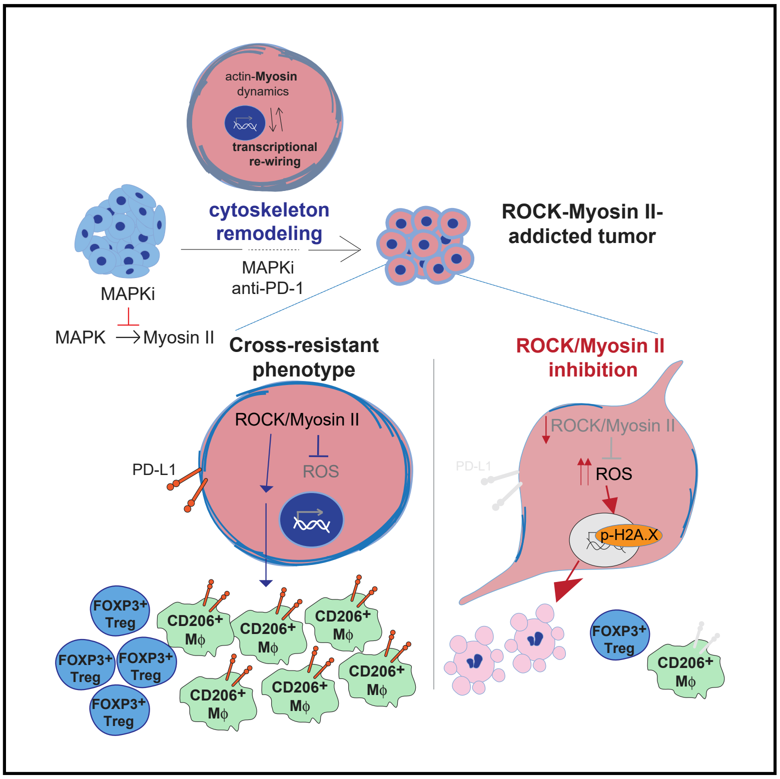

Authors

Jose L. Orgaz, Eva Crosas-Molist, Amine Sadok, ..., Sophia N. Karagiannis, Ilaria Malanchi, Victoria Sanz-Moreno

Correspondence

j.orgaz@qmul.ac.uk (J.L.O.), v.sanz-moreno@qmul.ac.uk (V.S.-M.)

In Brief

Orgaz et al. show that myosin II activity increases during melanoma adaptation to MAPK pathway inhibition. ROCK-myosin II signaling supports survival of resistant melanoma cells and promotes immunosuppression. ROCK inhibitors improve the efficacy of MAPK inhibitors and immunotherapies in melanoma models.

Highlights

- Therapy-resistant melanoma cells restore myosin II activity to increase survival
- High myosin II activity identifies targeted and immunotherapy-resistant melanomas
- ROCK-myosin II inhibition increases ROS-DNA damage and decreases PD-L1 and Tregs
- ROCK inhibition enhances efficacy of MAPK inhibitors and immunotherapies

Orgaz et al., 2020, Cancer Cell *37*, 85–103
January 13, 2020 © 2019 The Authors. Published by Elsevier Inc.
https://doi.org/10.1016/j.ccell.2019.12.003

CellPress

Myosin II Reactivation and Cytoskeletal Remodeling as a Hallmark and a Vulnerability in Melanoma Therapy Resistance

Jose L. Orgaz,[1,2,*] Eva Crosas-Molist,[1,2,10] Amine Sadok,[3,10] Anna Perdrix-Rosell,[1,2,4,10] Oscar Maiques,[1,2,10] Irene Rodriguez-Hernandez,[1,2,10] Jo Monger,[1] Silvia Mele,[5] Mirella Georgouli,[2] Victoria Bridgeman,[4] Panagiotis Karagiannis,[5,6] Rebecca Lee,[7] Pahini Pandya,[2] Lena Boehme,[2] Fredrik Wallberg,[8] Chris Tape,[9] Sophia N. Karagiannis,[5] Ilaria Malanchi,[4] and Victoria Sanz-Moreno[1,2,11,*]
[1]Barts Cancer Institute, Queen Mary University of London, John Vane Science Building, Charterhouse Square, London EC1M 6BQ, UK
[2]Randall Division of Cell and Molecular Biophysics, King's College London, New Hunt's House, Guy's Campus, London SE1 1UL, UK
[3]Translational Cancer Discovery Team, Cancer Research UK Cancer Therapeutics Unit, The Institute of Cancer Research, 15 Cotswold Road, Sutton, London SM2 5NG, UK
[4]Tumour Host Interaction, The Francis Crick Institute, 1 Midland Road, London NW1 1AT, UK
[5]St. John's Institute of Dermatology, King's College London & NIHR Biomedical Research Centre at Guy's and St. Thomas's Hospitals and King's College London, London SE1 9RT, UK
[6]Department of Oncology, Haematology and Stem Cell Transplantation, University Hospital of Hamburg Eppendorf, Hamburg 20246, Germany
[7]Molecular Oncology Group, Cancer Research UK Manchester Institute, Manchester M20 4BX, UK
[8]The Institute of Cancer Research, Chester Beatty Laboratories, 237 Fulham Road, London SW3 6JB, UK
[9]Cell Communication Lab, UCL Cancer Institute, 72 Huntley Street, London WC1E 6DD, UK
[10]These authors contributed equally
[11]Lead Contact
*Correspondence: j.orgaz@qmul.ac.uk (J.L.O.), v.sanz-moreno@qmul.ac.uk (V.S.-M.)
https://doi.org/10.1016/j.ccell.2019.12.003

SUMMARY

Despite substantial clinical benefit of targeted and immune checkpoint blockade-based therapies in melanoma, resistance inevitably develops. We show cytoskeletal remodeling and changes in expression and activity of ROCK-myosin II pathway during acquisition of resistance to MAPK inhibitors. MAPK regulates myosin II activity, but after initial therapy response, drug-resistant clones restore myosin II activity to increase survival. High ROCK-myosin II activity correlates with aggressiveness, identifying targeted therapy- and immunotherapy-resistant melanomas. Survival of resistant cells is myosin II dependent, regardless of the therapy. ROCK-myosin II ablation specifically kills resistant cells via intrinsic lethal reactive oxygen species and unresolved DNA damage and limits extrinsic myeloid and lymphoid immunosuppression. Efficacy of targeted therapies and immunotherapies can be improved by combination with ROCK inhibitors.

INTRODUCTION

Malignant melanoma has very poor survival rates (Balch et al., 2009) despite being at the forefront of personalized medicine (Lau et al., 2016). Mutant BRAF (V600) is the most common oncogene in melanoma (Davies et al., 2002), driving proliferation, survival, and tumor progression by hyper-activating MEK and ERK kinases (Gray-Schopfer et al., 2007). This led to BRAF^{V600E} inhibitors (BRAFi) development (Chapman et al., 2011; Flaherty et al., 2010; Zhang, 2015). Unfortunately, most patients had partial responses and disease progressed due to acquired resistance (Larkin et al., 2014; Robert et al., 2015; Zhang, 2015). Often, patients with resistance develop more metastases (Wagle et al., 2011) and 20% of BRAF mutant melanoma patients never respond to BRAFi due to intrinsic resistance (Zhang, 2015). Most resistance mechanisms involve MAPK reactivation

Significance

Resistance to therapies is a persistent problem in melanoma management. Here, we identify an adaptation strategy in response to either targeted therapies or immunotherapies. Under treatment, melanoma cells undergo cytoskeletal remodeling and consequent activation of ROCK-myosin II pathway. Such adaptation process renders resistant melanoma cells vulnerable to ROCK-myosin II inhibition, which can be exploited therapeutically.

This is an open access article under the CC BY license (http://creativecommons.org/licenses/by/4.0/).

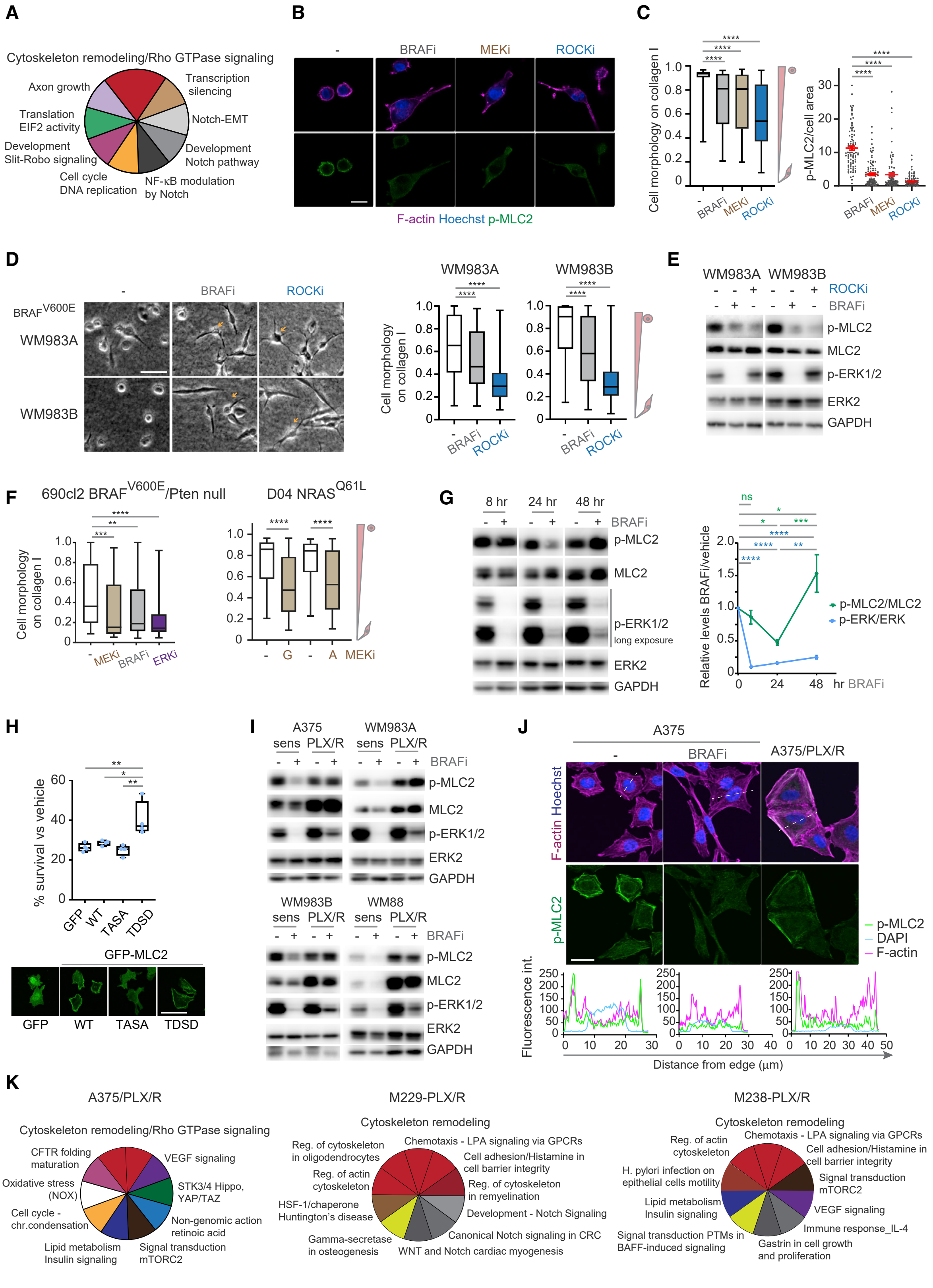

Figure 1. MAPK Regulates Myosin II Activity in Melanoma

(A) The 10 most enriched pathways in A375 cells after MEKi treatment compared with untreated cells from phospho-proteome data.

(B) p-MLC2 and F-actin confocal images of A375M2 cells on collagen I after treatment (BRAFi PLX4720, MEKi trametinib, ROCKi GSK269962A). Scale bar, 25 μm.

(legend continued on next page)

(Konieczkowski et al., 2018). Therefore, combination of a BRAFi with a MEK inhibitor (MEKi) was approved (Flaherty et al., 2012; Larkin et al., 2014; Long et al., 2014b). However, despite the improved responses, most patients still relapse (Flaherty et al., 2012; Konieczkowski et al., 2018).

Improved survival in patients with melanoma was reported after immune checkpoint inhibitor treatment (anti-PD-1 and anti-CTLA-4) (Hodi et al., 2010; Larkin et al., 2015; Sharma et al., 2017). However, there are patients who do not respond or relapse due to resistance (Sharma et al., 2017). Therefore, drug resistance is a persistent problem in melanoma management. Better understanding of the biological/biochemical changes in resistant cells will help develop improved treatments.

Given the overlap between migration and pro-survival pathways, drivers of resistance have been linked to metastatic ability (Alexander and Friedl, 2012). Importantly, cross-resistance to MAPK inhibitors (MAPKi) (Hugo et al., 2015) and immune checkpoint inhibitors (Hugo et al., 2016) has been described, involving transcriptomic alterations on genes key for epithelial-to-mesenchymal transition (EMT), metastasis/invasion, extracellular matrix (ECM) remodeling, hypoxia and angiogenesis (Hugo et al., 2015, 2016).

ROCK-myosin II pathway is a key regulator of invasive and metastatic behavior (Cantelli et al., 2015; Medjkane et al., 2009; Orgaz et al., 2014b; Sanz-Moreno et al., 2008, 2011). Non-muscle myosin II has contractile properties and is regulated by the phosphorylation of its light and heavy chains (Vicente-Manzanares et al., 2009). Myosin II-driven contractility relies on multiple kinases. Rho-kinase (ROCK) inactivates the myosin light chain 2 (MLC2) phosphatase, which leads to increased phosphorylation of MLC2 (p-MLC2) and myosin II activity (Ito et al., 2004; Olson, 2008). MLC2 is directly phosphorylated by ROCK and myosin light chain kinase (MLCK) (Vicente-Manzanares et al., 2009). ZIP kinase can also phosphorylate MLC2 directly and indirectly (Haystead, 2005). However, long-term depletion of ROCK1/2 cannot be substituted by any other kinase for generating actomyosin contractility (Kumper et al., 2016). Myosin II activity drives contractile forces required for migration (Clark et al., 2000; Lammermann and Sixt, 2009; Sahai and Marshall, 2002; Sanz-Moreno et al., 2008, 2011; Vicente-Manzanares et al., 2009; Wolf et al., 2003), metastatic colonization (Cantelli et al., 2015; Clark et al., 2000; Hall, 2012; Herraiz et al., 2015; Orgaz et al., 2014b; Sanz-Moreno et al., 2008, 2011), and aggressive amoeboid invasion (Cantelli et al., 2015; Medjkane et al., 2009; Orgaz et al., 2014b; Sanz-Moreno et al., 2008, 2011).

In vivo, ROCK inhibition diminishes tumor growth and metastatic spread (Itoh et al., 1999; Kumper et al., 2016; Sadok et al., 2015). However, the role of ROCK-myosin II during resistance to current cancer therapies has not been comprehensively investigated. Intriguingly, PAK contributes to MAPKi resistance (Lu et al., 2017) and Cdc42-PAK2-myosin II regulates amoeboid invasion (Calvo et al., 2011; Gadea et al., 2008).

Given the activation of pro-invasive/metastasis pathways during melanoma cross-resistance (Hugo et al., 2015, 2016), we sought to investigate the role of cytoskeletal remodeling in therapy resistance.

RESULTS

MAPK Regulates Myosin II Activity in Melanoma

To gain unbiased insight into molecular changes in melanoma cells after MAPKi, we analyzed the phosphoproteome of BRAF^{V600E} A375 melanoma cells early (24 h) on MEKi (GSK1120212 trametinib and PD184352) treatment (Figure 1A; Table S1). Using MetaCore Pathway enrichment analysis, we found that cytoskeletal remodeling and Rho GTPase signaling are top processes changing early on treatment (Figure 1A; Tables S1 and S2).

Because Rho GTPase regulates invasion via ROCK-myosin II activity and amoeboid behavior (Jaffe and Hall, 2005; Olson, 2008; Sadok et al., 2015; Sahai and Marshall, 2002; Sanz-Moreno et al., 2008), we studied how MAPK inhibition affected melanoma phenotypes on collagen I-recapitulating dermal environments (Cantelli et al., 2015; Orgaz et al., 2014b; Sanz-Moreno et al., 2008, 2011). Treatment of highly metastatic, amoeboid A375M2 melanoma cells with BRAFi PLX4720 and MEKi trametinib for 24 h led to loss of rounded-amoeboid behavior (Figure 1B). Inhibition of myosin II with ROCKi GSK269962A induced loss of circularity and a collapsed cytoskeleton (Figure 1B). Reduced myosin II activity (p-MLC2) was observed after BRAF, MEK, or ROCK inhibition (Figure 1C). Similar results were observed in other human and mouse melanoma cells and other MAPKi, including BRAF^{V600E} (WM983A, WM983B, 4599) (Figures 1D, 1E, S1A, and S1B), BRAF^{V600E}/Pten-null 690cl2 (Figures 1F and S1C) and NRAS$^{Q61L/R}$ (D04, MM485) (Figures 1F, S1D,

(C) Quantification of cell morphology and p-MLC2 by immunofluorescence from (B). Left, boxplot (n > 200 cells pooled from 3 experiments); right, mean ± SEM (n = 90 cells [dots] pooled from 3 experiments).

(D) Images and quantification of cell morphology on collagen I after treatment (BRAFi PLX4720, ROCKi GSK269962A) (n > 346 cells pooled from 2 experiments). Arrows show collapsed phenotype. Scale bar, 100 μm.

(E) p-MLC2 and p-ERK1/2 immunoblots from (D).

(F) Cell morphology on collagen I after treatment (690cl2, MEKi PD184352, BRAFi PLX4032, ERKi SCH772984, n = 50 cells; D04, MEKi GSK1120212, AZD6244, n = 125–150 cells).

(G) p-MLC2 and p-ERK1/2 levels after PLX4720 treatment (n = 5, mean ± SEM).

(H) Survival of A375 cells stably overexpressing wild type (WT), constitutively inactive TASA, or constitutively active TDSD MLC2 a after 5-day treatment with 0.1 μM PLX4720 (n = 4). Confocal images of GFP-MLC2. Scale bar, 50 μm.

(I) p-MLC2 and p-ERK1/2 immunoblots after PLX4720 treatment.

(J) p-MLC2 and F-actin confocal images (BRAFi PLX4720). Scale bar, 25 μm. Representative fluorescence intensity line scans (dashed lines in image) below.

(K) The 10 most enriched pathways in BRAFi-resistant A375/PLX/R (Girotti et al., 2013), M229-PLX/R, and M238-PLX/R cells (Titz et al., 2016) compared with parental cell lines from phospho-proteome data.

(A–F, I, and J) 24 h treatment.

(C, D, F, and H) Boxplots show median (center line); interquartile range (box); min-max (whiskers). p values by Kruskal-Wallis with Dunn's correction (C, D, and F), one-way ANOVA with Tukey's (H) or Benjamini, Krieger, and Yekutieli correction (G), *p < 0.05, **p < 0.01, ****p < 0.0001. See also Figure S1 and Tables S1 and S2.

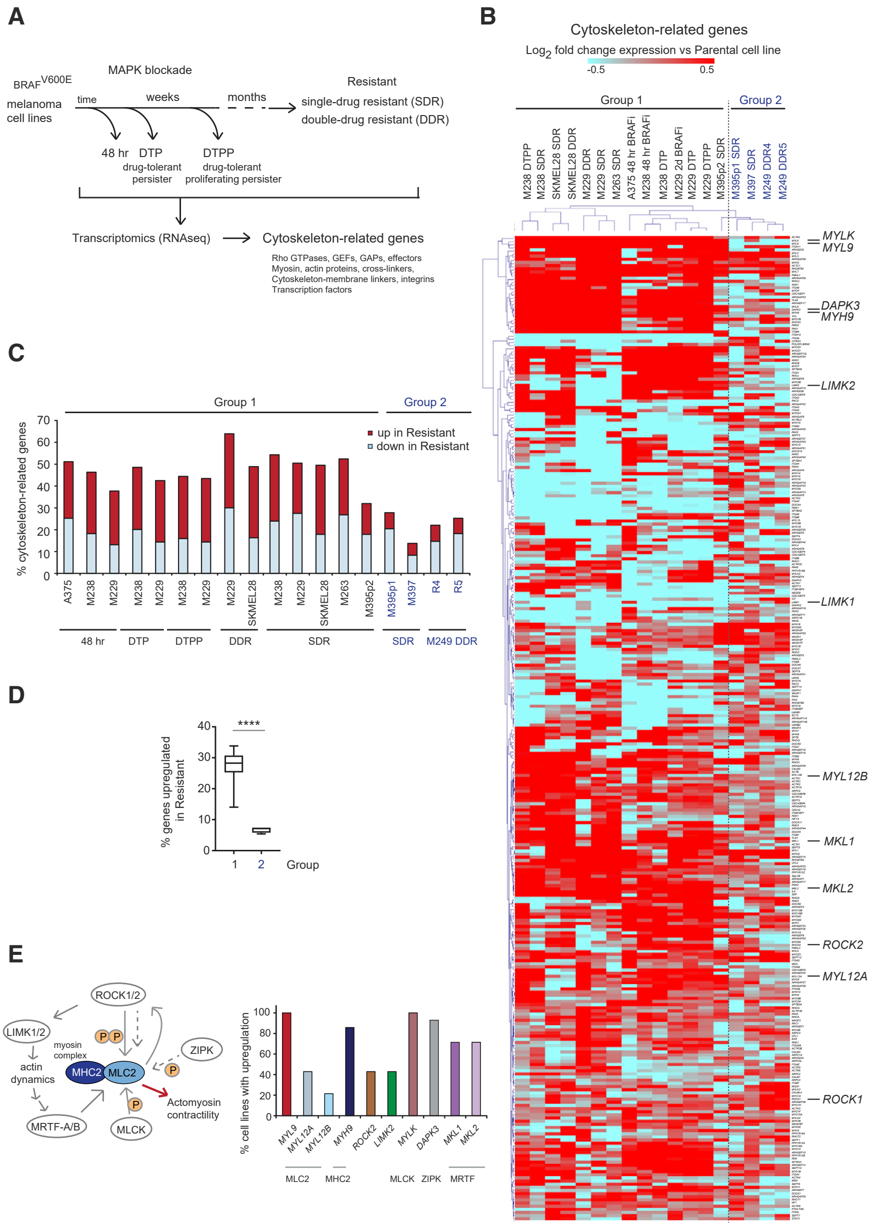

Figure 2. ROCK-Myosin II Pathway Is Transcriptionally Rewired during Development of Resistance

(A) Cell lines used for gene expression (Obenauf et al., 2015; Song et al., 2017).

(B) Heatmap of unsupervised hierarchical clustering of 313 cytoskeleton-related genes in A375 48 h BRAFi (Obenauf et al., 2015); M229-, M238-, SKMEL28-, M395p2-, M395p1-, and M249-derivatives (Song et al., 2017). Fold change expression in resistant versus parental lines is shown.

(C) Percentage of upregulated/downregulated (1.5-fold) cytoskeleton-genes versus parental line.

(D) Percentage of upregulated genes. Boxplot: median (center line); interquartile range (box); min-max (whiskers). p value by unpaired t test with Welch's correction, ****$p < 0.0001$.

(E) Left, schematic pathway. Right, percentage of group 1 cell lines with upregulation of indicated genes.

See also Table S3.

and S1E) cell lines. These data confirm that myosin II is regulated by MAPK in melanoma.

Restoration of ERK levels is observed during acquisition of resistance to MAPKi (Konieczkowski et al., 2018; Lito et al., 2012; Obenauf et al., 2015). Twenty-four hours after BRAFi reduced p-ERK was accompanied by reduced p-MLC2 (Figure 1G). However, 48 h after BRAFi treatment, p-MLC2 was restored concomitantly with very modest increase in p-ERK (Figure 1G). These data show that, early after treatment, cells remodel their cytoskeleton to recover myosin II activity, resulting in uncoupling of MAPK signaling from actomyosin.

We next hypothesized that, under therapy, myosin II could play a role in survival of cells with reduced MAPK activity. Strikingly, overexpression of a phosphomimetic MLC2 (TDSD) (Takaki et al., 2017) increased survival of A375 cells under BRAFi (Figures 1H and S1F). MLC2 overexpression did not affect p-ERK (Figure S1F). Moreover, high myosin II activity A375M2 cells were more resistant to BRAFi and MEKi compared with low metastatic, low myosin II activity A375 cells (Figures S1G and S1H). Similar results were observed using the pair WM983B (metastatic, high myosin II, and amoeboid) versus WM983A (primary tumor, low myosin II, and elongated) (Figures S1G and S1H). These data show that myosin II activity confers a survival advantage to BRAFi and could accelerate the onset of resistance. Accordingly, restored or increased p-MLC2 was seen in several BRAFi-resistant compared with parental cell lines (Figure 1I). MEKi did not affect p-MLC2 in resistant cells (Figure S1I), suggesting that MAPK-independent mechanisms may underlie p-MLC2 restoration. Importantly, cortical p-MLC2 was delocalized after 24-h BRAFi treatment in A375 cells and restored in BRAFi-resistant cells (Figure 1J). Phosphoproteomic analysis of several BRAFi-resistant melanoma cells compared with parental lines showed that cytoskeletal remodeling and Rho GTPase signaling were top enriched processes (Figure 1K; Table S2).

These data show that MAPK signaling regulates cytoskeletal myosin II and amoeboid behavior. During early responses to treatment, overexpression of myosin II allows melanoma cells to survive, independently of MAPK activity.

ROCK-Myosin II Pathway Is Transcriptionally Rewired during Development of Resistance

Transcriptomic alterations drive resistance to MAPK-targeted therapy (Hugo et al., 2015). Transcriptomic data of melanoma cells at different stages of MAPKi resistance (Figure 2A): 48 h

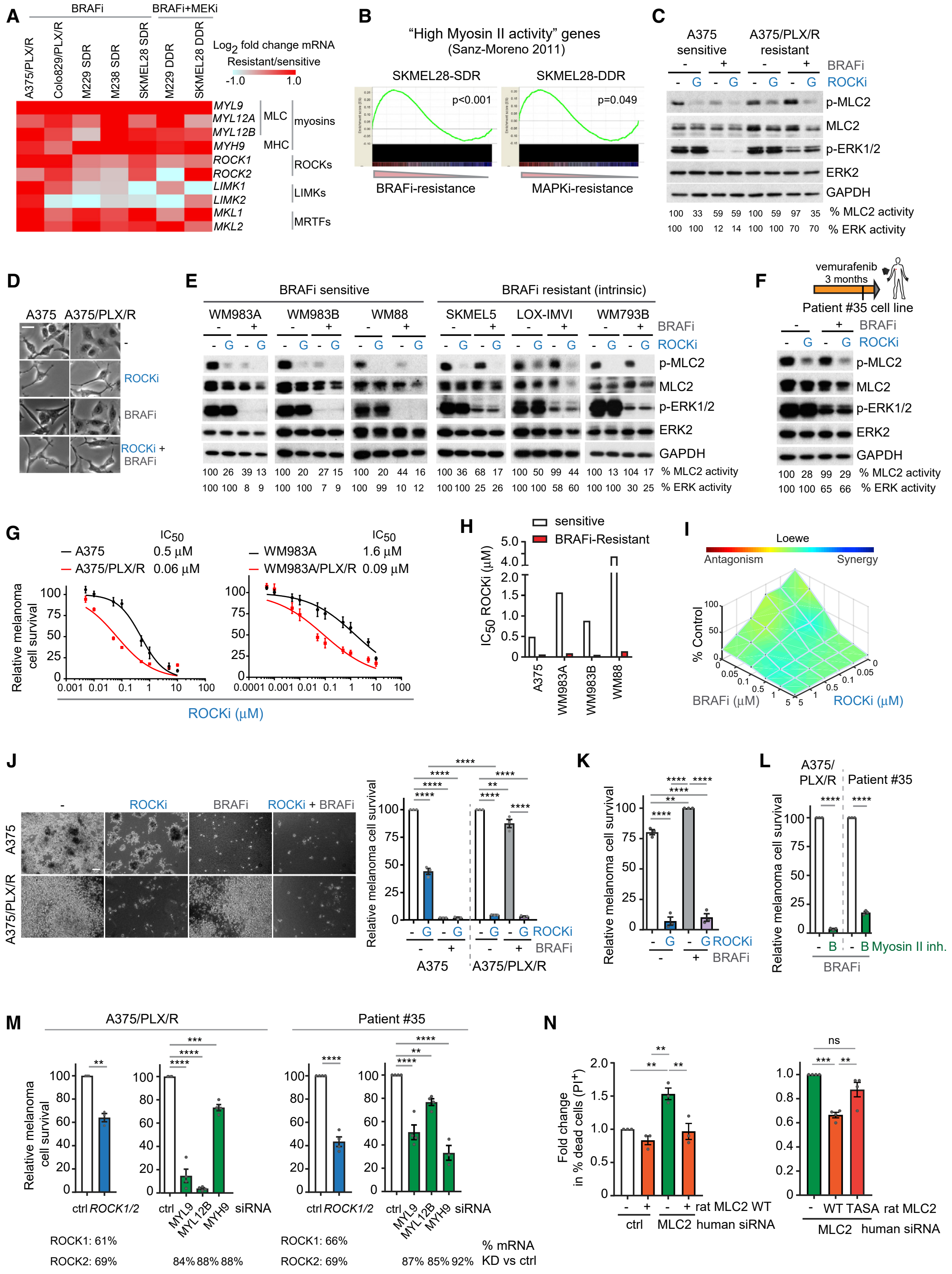

Figure 3. Survival of Targeted Therapy-Resistant Melanomas Is Dependent on ROCK-Driven Myosin II Activity

(A) Fold change in mRNA levels of ROCK-myosin II pathway genes in A375/PLX/R, Colo829/PLX/R by qRT-PCR (n = 3); and from published RNA sequencing data (Song et al., 2017).

(legend continued on next page)

(Obenauf et al., 2015; Song et al., 2017) or several weeks after treatment (drug-tolerant persisters [DTP], drug-tolerant proliferating persisters [DTPP]) (Song et al., 2017); and resistant cells after months-years (single-drug resistant [SDR, BRAFi], double-drug resistant [DDR, BRAFi + MEKi]) (Song et al., 2017) were used to analyze changes in 313 manually curated cytoskeletal-related genes (Table S3). Unsupervised hierarchical clustering classified melanoma cell lines into two groups (Figure 2B). Group 1 clustered the majority of cell lines, including 48-h BRAFi (when p-MLC2 was restored [Figure 1G]), DTP, DTPP, and SDR/DDR stages, which had a significant percentage of regulated genes (1.5-fold up- or downregulated) compared with baseline/sensitive cell-specifically upregulated genes (Figures 2C and 2D). Upregulated in group 1 were genes involved in generation/maintenance of myosin II-driven contractility (Figure 2E), such as myosin (MLC2 genes *MYL9*, *MYL12A/B*; and myosin heavy chain 2 [*MYH9*]), ROCK2, MLCK (*MYLK*), ZIPK (*DAPK3*), LIMK2, and transcriptional co-activator MRTF (*MKL1/2*), which directly regulates MLC2 expression (Medjkane et al., 2009). Of note, myosin II activity promotes myosin II expression to self-perpetuate (Calvo et al., 2013).

These data show that group 1 melanomas adapt to therapy by rewiring their transcriptome to alter cytoskeletal gene expression, ultimately restoring myosin II activity.

Survival of Targeted Therapy-Resistant Melanomas Is Dependent on ROCK-Driven Myosin II Activity

We next investigated if the ROCK-myosin II pathway could play a role in the survival of melanoma cells. Using qRT-PCR, we confirmed that MLC2 (*MYL9, MYL12A/B*) and other components of the ROCK-MLC2 pathway (*MYH9*, *ROCK1/2*, *LIMK*, *MKL1/2, MYLK*) were increased at the mRNA level in BRAFi-resistant cell line pairs (A375 and Colo829 cells, Figure 3A). Similar results were obtained using publicly available data from M229, M238, and SKMEL28 cells (Song et al., 2017) (Figure 3A). Gene set enrichment analysis (GSEA) showed that resistant cell lines displayed similar transcriptomes to cells with high myosin II activity (Figure 3B).

We compared the impact of MAPK inhibition on myosin II in sensitive/resistant melanoma cells. P-MLC2 was decreased after BRAFi treatment in sensitive but not in resistant A375/PLX/R cells. P-ERK was reduced by BRAFi in sensitive cells (Figures 3C and S2A). P-MLC2 in resistant cells was ROCK dependent, since several unrelated ROCKi (GSK269962A, H1152) (Feng et al., 2016) reduced p-MLC2 (Figures 3C and S2A). However, p-ERK was not affected by ROCKi.

Sensitive A375 cells lost circularity and became more spindle-shaped with long, thin protrusions after BRAF inhibition, with reduced p-MLC2 (Figures 1B–1G, 3D, and S2B). In contrast, A375/PLX/R cells did not change morphology after BRAFi treatment, while ROCKi decreased their circularity and promoted a collapsed (Sadok et al., 2015) cytoskeleton (Figures 3D and S2B).

We expanded these observations to PLX4720-resistant Colo829 (Figure S2C) and a panel of cell lines sensitive or intrinsically resistant to BRAFi (Baenke et al., 2015; Konieczkowski et al., 2014) (Figures 3E, S2D, and S2E). Similar results were observed in A375 cells resistant to BRAFi dabrafenib + MEKi trametinib (Flaherty et al., 2012; Long et al., 2014b) (A375/D + T/R) (Figures S2F and S2G); and in a resistant cell line established from a patient with acquired resistance to BRAFi (patient no. 35) (Figures 3F and S2H).

Because therapy-resistant cells maintain high p-MLC2 (Figure 1I) and that myosin II increases survival under therapy (Figure 1H), we assessed if myosin II could play a role in conferring a survival advantage to therapy-resistant cells. Reduced p-MLC2 after ROCKi impaired survival of sensitive and BRAFi-resistant melanoma pairs (A375, WM983A, WM983B, WM88) (Figures 3G, 3H, and S3A–S3C). BRAFi-resistant melanomas were 4- to 30-fold more sensitive to ROCKi GSK269962A (Figures 3G, 3H, S3A, and S3C) and AT13148 (Figure S3A). Moderate synergistic effects between ROCKi and BRAFi were observed in BRAFi-sensitive A375 cells (Figures 3I and S3D). More pronounced synergy was observed by annexin V/propidium iodide (PI) cell death staining (Figure S3E).

Importantly, A375/PLX/R cells grown on collagen I had increased sensitivity to ROCKi (Figure 3J). We observed impaired survival after ROCKi treatment in several models of drug resistance: A375/PLX/R, A375/D + T/R, Colo829 and BRAFi-intrinsic resistant lines (Figure S3F); and patient no. 35 cells (Figures 3K and S3G). Importantly, the survival advantage was provided by myosin II itself, since myosin II inhibitor blebbistatin strongly suppressed survival (Figures 3L and S3H). Moreover, siRNA targeting *ROCK1/2*, *MYL9*, *MYL12B*, or *MYH9*

(B) GSEA comparing high myosin II activity signature (Sanz-Moreno et al., 2011) to resistant cell lines (Song et al., 2017). Nominal p values shown, false discovery rate (FDR) < 0.2.
(C) p-MLC2 and p-ERK1/2 immunoblots after 24 h treatment.
(D) Images of cells from (C). Scale bar, 50 μm.
(E and F) p-MLC2 and p-ERK1/2 immunoblots of sensitive and intrinsically resistant cells (E); and patient no. 35 cells (F) after 24 h treatment (8 h for WM88). Vertical line in diagram (F): cell line establishment.
(G) Survival and half maximal inhibitory concentration (IC_{50}) values after a 3-day treatment (n = 3).
(H) IC_{50} values for GSK269962A.
(I) Cell survival as synergy graph of A375 cells treated for 3 days (n = 4).
(J) Images and quantification of cell survival on collagen I for 9 days (n = 3). Scale bar, 100 μm.
(K) Survival of patient no. 35 cells after 10 days (n = 3).
(L) Survival after a 5- to 10-day blebbistatin and PLX4720 treatment (n = 3).
(M) Survival 8 days after gene depletion by RNAi (n = 3; n = 4 A375/PLX/R myosin genes, patient no. 35 *MYL12B*, *ROCK1/2*). mRNA KD (percentage decrease versus control) by RT-PCR shown.
(N) Cell death in A375/PLX/R cells 3 days after transient MLC2 KD and rescue with rat MLC2 WT or TASA (n = 3, left graph; n = 4, right graph).
(C–K) ROCKi GSK269962A, BRAFi PLX4720.
Graphs show mean ± SEM and individual data points (circle). p values by one-way ANOVA with Tukey's (J, K, and N) or Dunnett's correction (M, myosin genes); t test with Welch's correction (L and M, *ROCK*), $^{**}p < 0.01$, $^{***}p < 0.001$, $^{****}p < 0.0001$; n.s., not significant. See also Figures S2 and S3.

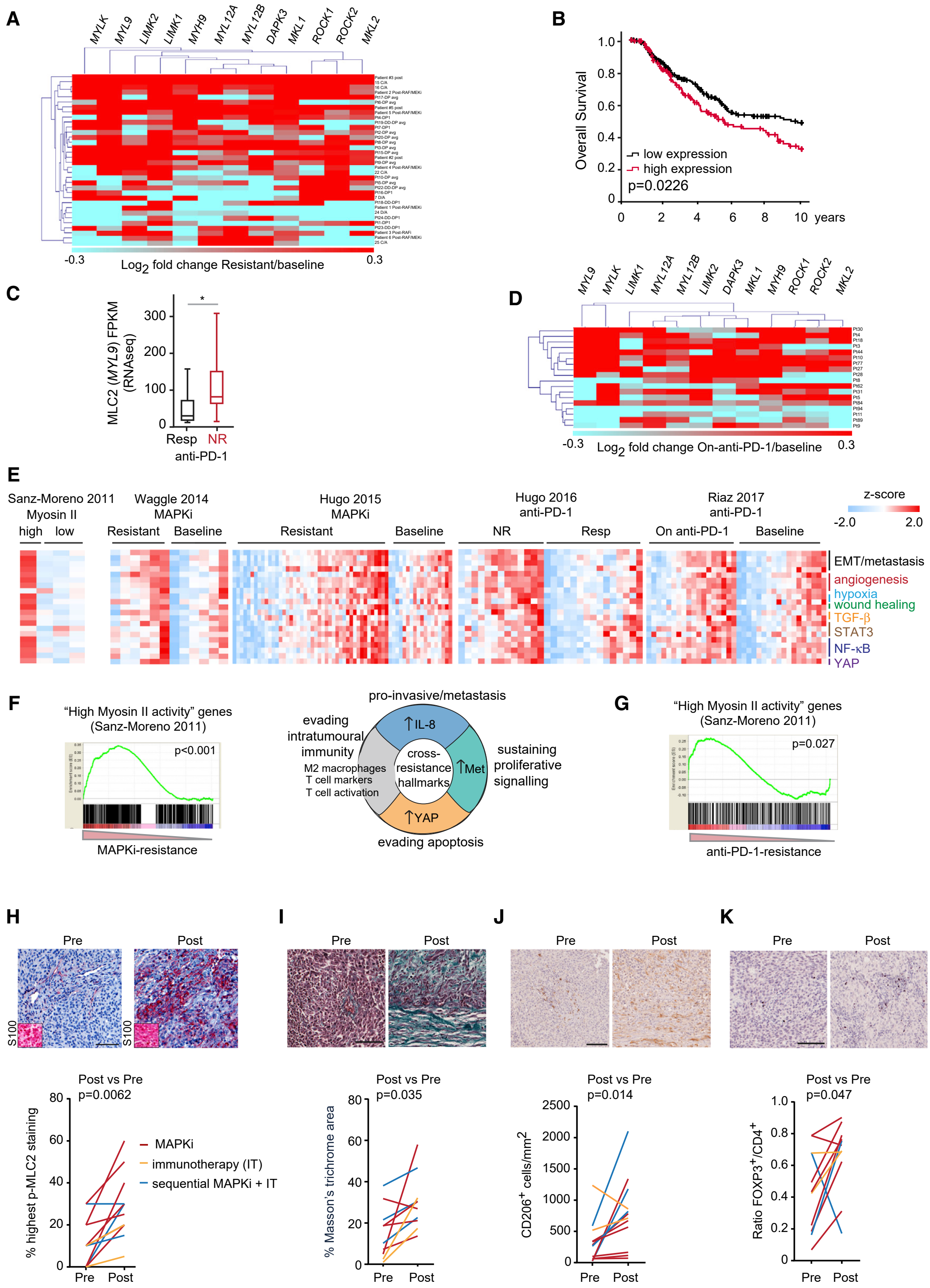

Figure 4. High Myosin II Levels Identify Therapy-Resistant Melanomas in Human Samples

(A) Heatmap of fold change in expression of ROCK-myosin II pathway genes in MAPKi-resistant versus baseline patient samples from (Hugo et al., 2015; Kwong et al., 2015; Sun et al., 2014; Wagle et al., 2014).

(B) Kaplan-Meier overall survival from The Cancer Genome Atlas according to expression of ROCK-myosin II genes (listed in A) (n = 389 melanoma patients).

(legend continued on next page)

reduced survival in A375/PLX/R and patient no. 35 cells (Figure 3M). The decrease in survival after MLC2 knockdown (KD) was more pronounced in BRAFi-resistant cells (Figure S3I). Therefore, both MLC2 expression and phosphorylation by ROCK are required to promote survival of resistant cells. Importantly, RNAi-insensitive rat MLC2 (Calvo et al., 2013) overexpression rescued the decreased survival observed after MLC2 depletion. This mechanism relied on MLC2 phosphorylation, since rescue was impaired by TASA-MLC2 inactive phospho-mutant (Figures 3N and S3J).

Overall, myosin II restoration confers a survival advantage to resistant melanomas.

High Myosin II Levels Identify Cross-Resistant Melanomas in Human Samples

We next validated our findings in clinical samples from published datasets (Hugo et al., 2015; Kakavand et al., 2017; Kwong et al., 2015; Long et al., 2014a; Rizos et al., 2014; Song et al., 2017; Sun et al., 2014; Wagle et al., 2014) (Table S4). There was a subset of melanoma tumors (~50%) with upregulation of ROCK-myosin II pathway genes (Figures 4A, S4A, and S4B), in accordance with data with resistant cell lines (Figure 2E). The Cancer Genome Atlas data showed that higher levels of ROCK-myosin II genes in treatment-naive melanoma patients confer worse prognosis (Figure 4B). MAPKi-resistant tumors quickly progress after relapse (Wagle et al., 2011), indicative of aggressiveness. We suggest that melanomas with intrinsically higher expression of the ROCK-myosin II pathway are more aggressive and prone to develop resistance.

Innately anti-PD-1-resistant (IPRES) tumors harbor a transcriptional signature of upregulated genes involved in the regulation of EMT, cell adhesion, ECM remodeling, angiogenesis, and hypoxia (Hugo et al., 2016). MAPK-targeted therapies in melanoma induce similar signatures with immunosuppressive features (Hugo et al., 2015). These studies suggest that non-genomic MAPKi resistance driven by transcriptional upregulation of metastasis-related pathways mediates cross-resistance to anti-PD-1 therapy. They also suggest that aggressive tumors resistant to one therapy (e.g., MAPKi) will likely not respond to second therapy (anti-PD-1). Therefore, we next investigated if ROCK-myosin II could predict anti-PD-1 responses as part of a cross-resistance mechanism. Samples before anti-PD-1 treatment (Hugo et al., 2016) showed higher *MYL9* expression in non-responding (NR) than in responding (Resp) patients (Figure 4C). Increased levels of ROCK-myosin II pathway genes were detected in a large subset of patients on anti-PD-1 treatment (Riaz et al., 2017) (Figures 4D and S4C).

We have previously generated a transcriptional signature for amoeboid metastatic melanoma cells harboring high ROCK-driven myosin II activity (Cantelli et al., 2015; Sanz-Moreno et al., 2011). We compared high myosin II signature; MAPK-targeted therapy-resistant signatures (Hugo et al., 2015; Sun et al., 2014; Wagle et al., 2014); anti-PD-1/NR signature (Hugo et al., 2016); and on-anti-PD-1-treatment signature (Riaz et al., 2017). Single sample GSEA (ssGSEA) showed that similar gene signatures are enriched in high myosin II amoeboid cells and therapy-resistant patient samples (Figure 4E), including EMT/metastasis, angiogenesis, hypoxia, wound healing, transforming growth factor β (TGF-β)-, STAT3-, nuclear factor κB-, and YAP-signaling genes (Table S5).

Global GSEA analysis showed a significant overlap between "high myosin II activity" melanoma cells (Cantelli et al., 2015; Sanz-Moreno et al., 2011) and MAPKi-resistant melanomas with immunosuppressive macrophages, and pro-invasive and pro-survival features (Hugo et al., 2015) (Figure 4F). There was significant overlap between high myosin II and anti-PD-1/NR patient signatures (IPRES [Hugo et al., 2016]) (Figure 4G).

Myosin II-driven contractility is regulated by MLC2 gene expression and phosphorylation/activity (Calvo et al., 2013; Medjkane et al., 2009; Olson, 2008). We assessed p-MLC2 levels in paired patient melanoma sections before and after therapy (targeted therapy, immunotherapy [IT], or sequential targeted and IT; Table S6). P-MLC2 levels were higher in all resistant tumors after treatment (Figures 4H and S4D–S4G). Specificity of p-MLC2 antibody was validated by RNAi (Figure S4D). Collagen density promotes myosin II activity (Laklai et al., 2016; Paszek et al., 2005), and ROCK-myosin II induces ECM stiffening (Samuel et al., 2011). Increased ECM deposition was observed in resistant compared with pre-treatment samples (Figures 4I and S4E–S4G). Melanoma cells with high ROCK-myosin II are highly secretory and polarize macrophages to tumor-promoting ($CD206^+$) phenotypes (Georgouli et al., 2019). Interestingly, $CD206^+$ cells were increased in resistant compared with pre-treatment samples (Figures 4J and S4E–S4G), correlating with higher p-MLC2 (Figure 4H). Immunosuppressive $FOXP3^+$ regulatory T cells (Tregs)/$CD4^+$ ratio was also increased in resistant samples (Figures 4K and S4F–S4G). These data suggest that high MLC2 (*MYL9*) expression and/or activation (p-MLC2) in melanoma cells together with immunosuppressive populations and higher collagen densities identify therapy-resistant melanomas, suggesting their potential as biomarkers.

Overall, resistant tumors and melanomas with high myosin II activity harbor a similar transcriptome. Importantly, ROCK-myosin II could be a key mediator of non-genomic cross-resistance.

(C) *MYL9* mRNA in Resp (n = 15) and NR (n = 13) anti-PD-1 patients from (Hugo et al., 2016). Boxplot: median (center line); interquartile range (box); min-max (whiskers).

(D) Heatmap of fold change in expression of ROCK-myosin II genes in on-anti-PD-1 versus baseline patient samples (Riaz et al., 2017).

(E) Heatmaps show ssGSEA of cross-resistance gene signatures (NR, non-responder; Resp, responder).

(F and G) GSEA comparing "high myosin II activity" signature (Sanz-Moreno et al., 2011) to a subset of MAPKi-resistant patient samples from (Hugo et al., 2015) (F) or anti-PD-1/NR samples (Hugo et al., 2016) (G). Chart pie in (F) with cross-resistance hallmarks from (Hugo et al., 2015). Nominal p values shown, FDR < 0.001 (F) and 0.145 (G).

(H–K) Images (patient no. 17) and quantification in 12 paired samples before and after therapies (including those in Figures S4E and S4F) of: p-MLC2 (% cells with highest score), melanoma marker S100 (inset) (H); Masson's trichrome staining (percentage stained area/section) (I); $CD206^+$ cells (J); $FOXP3^+$ cells (K). Scale bars, 100 μm.

p values by Mann-Whitney test (C, H–K). See also Figure S4 and Tables S4, S5, and S6.

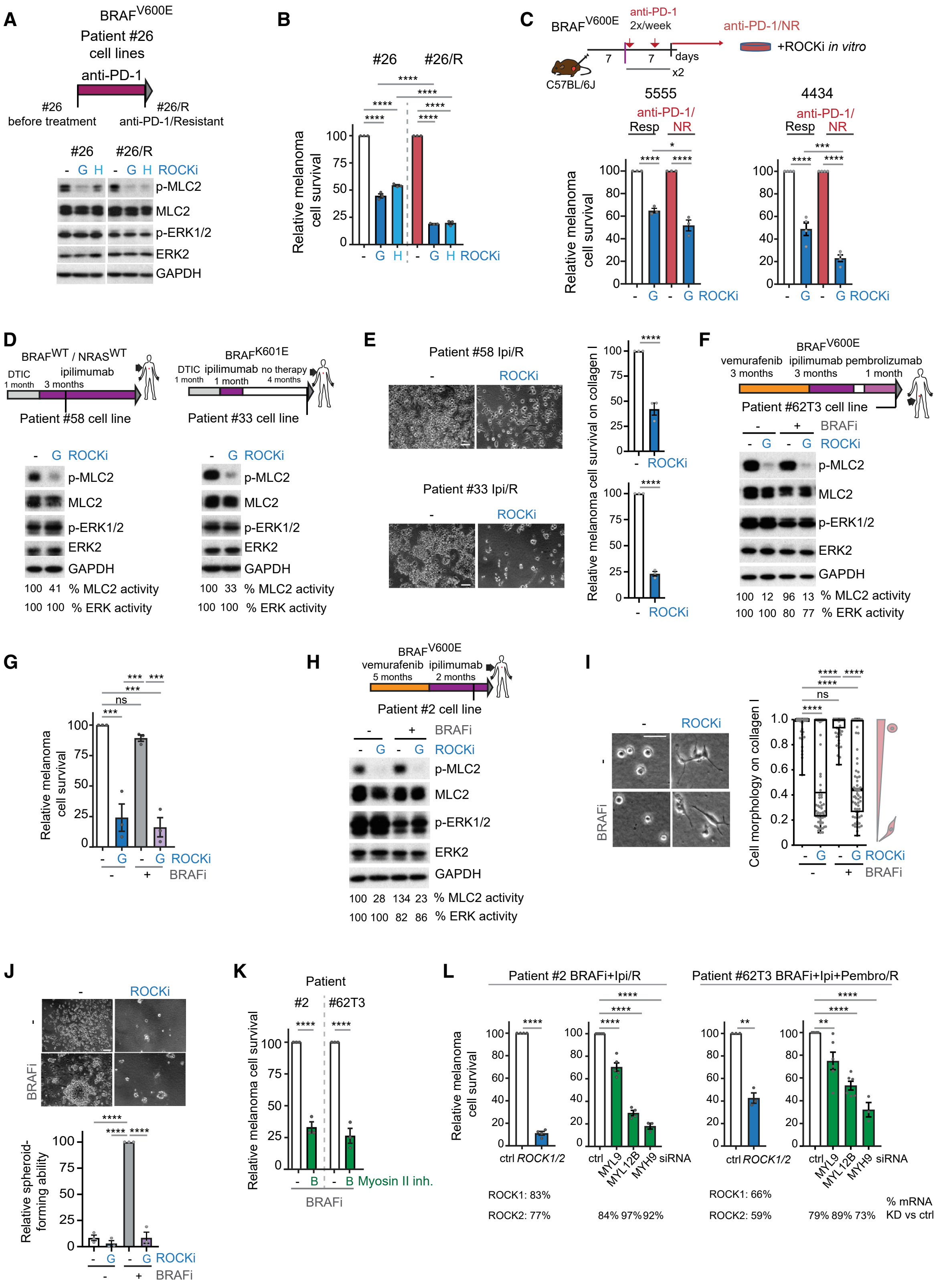

Figure 5. ROCK-Driven Myosin II Activity in Immunotherapy-Resistant Melanoma

(A) Top, schematic of cell lines. Bottom, p-MLC2 immunoblots after treatment: n = 7 (G); n = 3 (H).

(B) Survival of patient no. 26 cells treated for 10 days (n = 4).

(legend continued on next page)

ROCK-Driven Myosin II Activity in Immunotherapy-Resistant Melanoma

Next we investigated whether survival of immunotherapy-resistant melanomas could be dependent on ROCK-myosin II. To test this hypothesis *in vitro*, we used patient no. 26-derived cells established pre- and post-anti-PD-1 resistance (Figure 5A). Both cell lines rely on ROCK to sustain p-MLC2 (Figures 5A and S5A). Importantly, anti-PD-1/resistant cells were 2-fold more sensitive to ROCKi (Figure 5B). Increased sensitivity was further confirmed in a resistant brain metastasis-derived cell line from patient no. 26 (data not shown). We then grafted mouse Braf^{V600E} melanoma cell lines 5555 and 4434 cells (Dhomen et al., 2009) subcutaneously onto fully immunocompetent C57BL/6J mice and treated with anti-PD-1, which led to variable responses. We isolated NR and Resp tumors and grew them *ex vivo* (Figures S5B and S5C). Increased intrinsic sensitivity to ROCKi *in vitro* was found in anti-PD-1/NR-derived cells (Figure 5C), similar to the resistant human cell lines (Figure 5B). As melanoma cells activate an immune-evasion program they also trigger cytoskeletal remodeling, rendering them intrinsically vulnerable to ROCK-myosin II inhibition.

Using additional cell lines established from human melanomas resistant to immunotherapy (patients no. 58 and no. 33), we confirmed that these melanomas harbored ROCK-dependent p-MLC2 levels (Figures 5D and S5D). Cell survival was impaired after treatment with several ROCKi on 3D (Figures 5E and S5E) and 2D culture (Figure S5F).

Our data predict that cells that do not respond to MAPKi—if they undergo cross-resistant transcriptional rewiring of their cytoskeleton—they will not respond to immunotherapy either. Such cross-resistance will be susceptible now to ROCKi. Patient no. 62T3 cell line was established from a tumor with acquired resistance to BRAFi and developed primary resistance to anti-CTLA-4 and anti-PD-1 (Figure 5F). After BRAF inhibition, p-MLC2 was not affected in these cells, while ROCK inhibition decreased p-MLC2 (Figures 5F and S5G). Similar to our previous data, survival of patient no. 62T3 cells was impaired with ROCKi (Figures 5G and S5H).

Similarly, patient no. 2 cells were established from a tumor that never responded to targeted and immunotherapy (Figure 5H). The post-treatment-resistant biopsy had higher p-MLC2 compared with baseline tumor (Figure S4F). Similar to patient no. 62T3, BRAFi did not affect p-MLC2, while ROCKi decreased p-MLC2 in patient no. 2 cells (Figures 5H and S5I). Patient no. 2 cells on collagen I displayed very rounded morphology even in the presence of BRAFi, indicative of high p-MLC2 (Figure 5I). ROCKi decreased circularity and induced very thin protrusions and a spindle-shaped morphology in patient no. 2 cells. A common event during melanoma resistance is BRAFi/MEKi addiction, which occurs when resistant melanomas become drug dependent (Das Thakur et al., 2013; Hong et al., 2017; Kong et al., 2017; Moriceau et al., 2015; Sun et al., 2014). Patient no. 2 cells displayed addiction to BRAFi on 2D cultures (Figure S5J), but treatment with ROCKi impaired survival in the presence of BRAFi and further decreased survival upon BRAFi withdrawal (Figure S5J). This agrees with data on BRAFi-resistant patient no. 35 and Colo829/PLX/R cells (Figures 3K, S3F, and S3G), which also displayed varying degrees of BRAFi addiction. Interestingly, patient no. 2 cells grew as compact spheroids on collagen I under BRAFi treatment, but growth was abrogated by ROCKi (Figures 5J and S5K), showing that myosin II drives survival in BRAFi-addicted cells. Accordingly, myosin II inhibition with blebbistatin or RNAi against ROCK or myosin II genes impaired survival of patient no. 2 and no. 62T3 cells (Figures 5K, 5L, and S5L).

MRTF controls MLC2 expression (Medjkane et al., 2009) while MRTF activity is regulated by actin dynamics (Posern and Treisman, 2006). Expression of MRTF (*MKL*) was increased in resistant melanomas (Figures 2E and 4A) and its depletion impaired BRAFi-resistant cell survival (Figure S5M). Accordingly, *MYL9* mRNA levels decreased after MRTF depletion (Figure S5M).

Overall, melanomas with acquired and primary resistance to targeted and immunotherapies rely on myosin II activity for their survival. Consistently, p-MLC2 levels and cancer cell survival were positively correlated in resistant lines (Figure S5N).

ROCK-Myosin II Inhibition Induces Lethal Reactive Oxygen Species, DNA Damage, and Cell-Cycle Arrest

We next investigated why resistant cells rely on myosin II for survival. Resistant cells (Song et al., 2017) were enriched in oxidative stress and reactive oxygen species (ROS) metabolism gene signatures (Figure 6A) and had lower DNA damage repair gene expression (Figure 6B). Interestingly, ROCK-myosin II suppresses high ROS in migrating cells (Herraiz et al., 2015). ROCKi

(C) Top, schematic of experiment. Bottom, survival of 4434 and 5555 anti-PD-1/non-responder (NR) lines versus responder (Resp) after a 3-day treatment (n = 3, 5555; n = 4, 4434).
(D) p-MLC2 and p-ERK1/2 immunoblots of patient no. 58 (n = 4) and no. 33 (n = 3) cells after treatment.
(E) Images and quantification of cell survival on collagen I for 7 days (n = 3).
(F) p-MLC2 and p-ERK1/2 immunoblots of patient no. 62T3 cells after treatment (n = 3).
(G) Survival of patient no. 62T3 cells after a 10-day treatment (n = 3).
(H) p-MLC2 and p-ERK1/2 immunoblots of patient no. 2 cells after treatment (n = 5).
(I) Cell morphology of patient no. 2 cells on collagen I after treatment. n = 70 cells (dots) from 2 experiments. Scale bar, 50 μm.
(J) Survival of patient no. 2 cells as spheroid-forming ability on collagen I for 16 days (n = 3); Scale bar, 100 μm.
(K) Survival after a 10-day blebbistatin treatment (n = 3).
(L) Survival 8 days after gene depletion by RNAi (n = 3; n = 4 patient no. 2 *MYL12B-ROCK1/2*, no. 62T3 *MYL9*; n = 5 no. 62T3 *MYL12B*). Average percentage mRNA KD (percentage decrease versus control) by qRT-PCR is shown. Vertical line in (D, F, and H): cell line establishment.
(A–J) ROCKi GSK269962A, H1152; (F–K) BRAFi PLX4720.
(A, D, F, H, and I) 24 h treatment.
Graphs show mean ± SEM and individual data points (circle) except boxplot in I (median, center line; interquartile range, box; min-max, whiskers). p values by one-way ANOVA with Tukey's (B, C, G, and J) or Dunnett's correction (L, myosin genes); Kruskal-Wallis with Dunn's correction (I), t test with Welch's correction (E, K, and L, *ROCK*); **p < 0.01, ***p < 0.001, ****p < 0.0001; n.s., not significant. See also Figure S5.

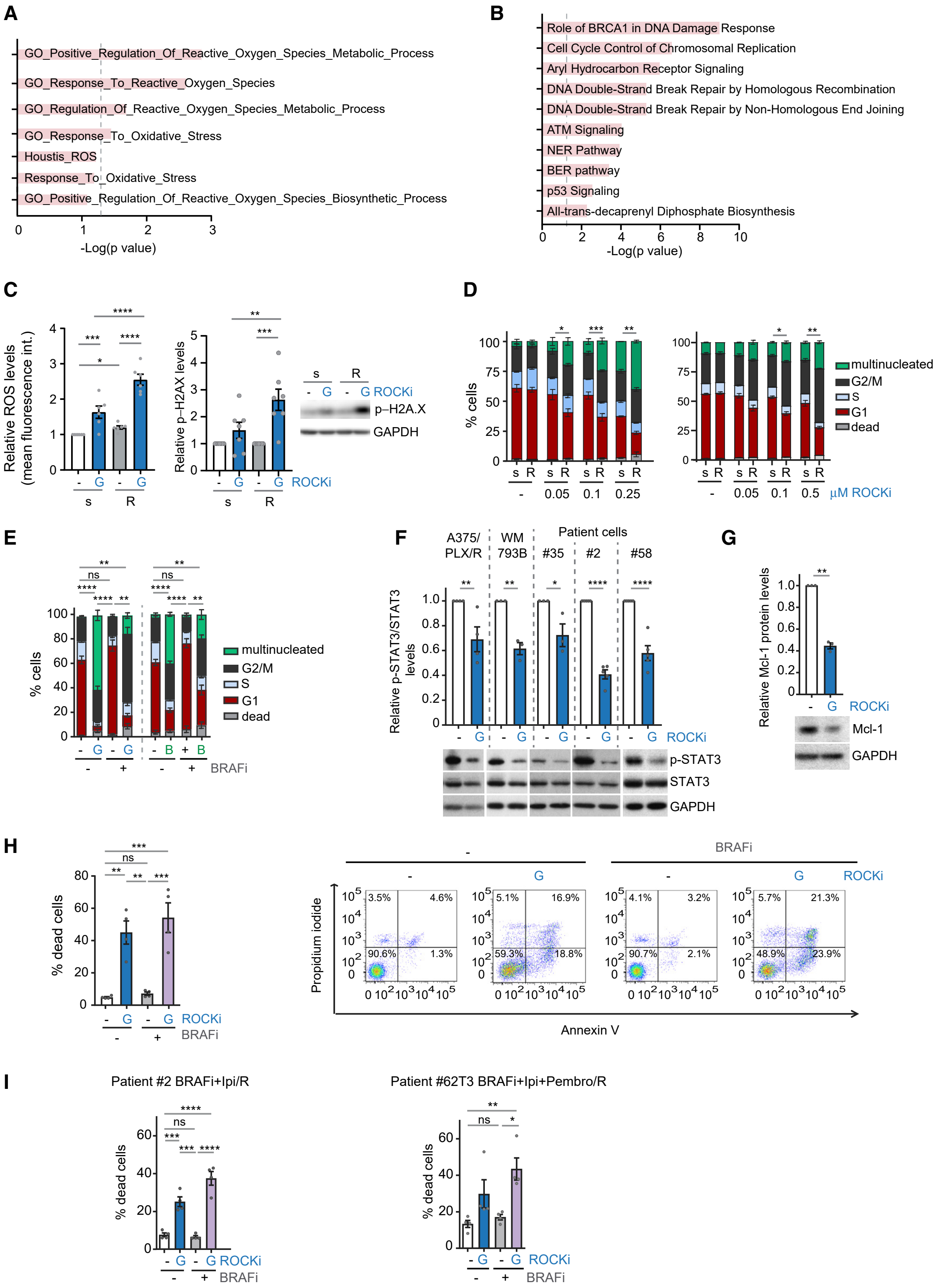

Figure 6. ROCK-Myosin II Inhibition Induces Lethal ROS, DNA Damage, and Cell-Cycle Arrest

(A) GSEA of ROS/oxidative stress-related gene signatures in MAPKi-resistant versus sensitive cell lines (group 1) from (Song et al., 2017). Dashed line indicates statistical significance.

(B) The 10 most enriched canonical pathways in downregulated genes in MAPKi-resistant cell lines (group 1) from (Song et al., 2017).

(C) Left, ROS levels in A375 (s) and A375/PLX/R (R) cells after treatment (n = 6). Right, quantification of p-H2A.X immunoblots (n = 7).

(legend continued on next page)

induced higher levels of ROS (Figures 6C and S6A) and phosphorylated H2A.X (p-H2A.X), indicative of DNA damage (Figure 6C), in BRAFi-resistant cells compared with sensitive cells. Resistant cells had lower expression of genes of the base excision repair pathway (Figure S6B) that repairs ROS-mediated DNA damage (Krokan and Bjoras, 2013).

Because BRAFi-resistant cells harbor higher ROS and have lost DNA damage repair machinery, ROCKi increases ROS levels leading to unrepaired DNA damage. Unrepaired DNA damage can induce cell-cycle arrest that, if prolonged, can lead to cell death (Shaltiel et al., 2015). Blocking myosin II activity using ROCKi resulted in a pronounced dose-dependent cell-cycle arrest in BRAFi-resistant melanomas (Figures 6D and S6C). Blebbistatin caused very similar results in resistant cells (Figure 6E). As a result of ROS-DNA damage, resistant cells suffer G2-M arrest and multinucleation. Accordingly, time-lapse video microscopy showed that cells suffering cell-cycle arrest died after 72 h (Figure S6D).

ROS production is counterbalanced by STAT3 (Poli and Camporeale, 2015) and both high myosin II activity and resistant cells harbor high STAT3 signaling (Figure 4E). ROCKi decreased p-STAT3 levels and its pro-survival target Mcl-1 in both targeted therapy- and immunotherapy-resistant cells (Figures 6F and 6G). Moreover, we measured decreased survival in A375/PLX/R cells after 72 h of ROCKi treatment using 3-(4,5-dimethylthiazol-2-yl)-2,5-diphenyl tetrazolium bromide assay (Figure S6E). Annexin V/PI staining (Figure S6F) showed increased cell death after ROCKi treatment in A375/PLX/R (Figures 6H and S6G), patient no. 2 (Figures 6I and S6H), no. 62T3 (Figure 6I), and no. 35 cells (Figure S6I).

Therefore, ROCK-driven myosin II protects tumor cells from toxic ROS levels, enabling correct cell-cycle progression and providing pro-survival signals. Because resistant cells have altered ROS and loss of DNA damage repair genes, ROCK-myosin II inhibition is particularly detrimental.

Combining ROCK Inhibitors with BRAF Inhibitors *In Vivo*

To translate our findings to pre-clinical *in vivo* models, we combined BRAFi and ROCKi (low dose) GSK269962A in BRAFi-resistant A375/PLX/R xenografts in nude mice. Mice tolerated drug treatments well (Figure S7A). The combination treatment was the most efficient and induced regression of tumors and improved mouse survival (Figures 7A and S7B).

Patient no. 2 cells displayed PLX4720 addiction *in vitro* (Figures 5J, S5J, and S5K) and also *in vivo* (Figure 7B), as seen by increased growth in the presence of PLX4720. ROCKi reduced growth and p-MLC2 levels of PLX4720-resistant patient no. 2 xenografts (Figures 7B, 7C, and S7C).

High myosin II activity provides an advantage during early survival in the lung, which is a limiting step in the metastatic process (Cantelli et al., 2015; Medjkane et al., 2009; Orgaz et al., 2014b; Sanz-Moreno et al., 2008, 2011). Many of the cross-resistance gene signatures were related to metastatic programs (Figure 4E). Survival of patient no. 2 cells in the lung after tail vein injection was improved after pre-treatment *in vitro* with BRAFi (Figure 7D). However, when pre-treated with ROCKi, survival was impaired (Figure 7D). Patient no. 35 BRAFi-addicted cell line (Figure 3K) showed reduced growth and p-MLC2 levels *in vivo* after ROCKi (Figures 7E and S7C).

High myosin II activity cells (Cantelli et al., 2015; Sanz-Moreno et al., 2011) and MAPKi-resistant melanomas with immunosuppressive features and pro-tumorigenic macrophages (Hugo et al., 2015) display transcriptional overlap (Figure 4F). We assessed myosin II activity and immunosuppressive populations in A375/PLX/R xenografts (Figure 7A). ROCKi-treated tumors had reduced p-MLC2 (Figure 7F) and lower number of CD206$^+$ macrophages (Figure 7G), which could contribute to reduced tumor growth. ROCKi decreased polarization to CD206$^+$ macrophages as F4/80$^+$ content was not affected (Figure S7D), only CD206$^+$/F4/80$^+$ ratio (Figure 7G). ROCK-myosin II inhibition could overcome cross-resistance to targeted/immunotherapies via intrinsic cell survival and extrinsic myeloid co-option.

ROCK-Myosin II Inhibition Improves Efficacy of Immune Checkpoint Inhibitors

As high myosin II identifies anti-PD-1/NR, we tested whether ROCKi could be given as combination therapy to improve response to anti-PD-1. We allografted treatment-naive 5555 cells into immunocompetent mice. Anti-PD-1 combined with ROCKi (combo) induced significantly more regressions of established tumors compared with single treatments (Figures 8A and S8A), and treatments were well tolerated based on weight (Figure S8B). ROCKi-treated tumors had reduced p-MLC2 after 5 days of treatment or at endpoint (Figures 8B and S8C). ROCKi also decreased immunosuppressive cell populations at both 5 days and endpoint: CD206$^+$ macrophages (Figures 8C and S8D) and FOXP3$^+$ Tregs (Figures 8D and S8D). F4/80$^+$ (Figure S8D) and other immune populations (CD3$^+$, CD4$^+$, CD8$^+$ cells) were not significantly affected by ROCKi in tumors or spleens (Figures S8E and S8F). ROCKi did not affect percentage of CD4$^+$ and CD8$^+$ cells expressing PD-1 (data not shown). CD206$^+$ polarization mainly occurred in tumors since polarization in the spleens was less than 1% (Figure S8F).

We analyzed infiltration in the tumor body (TB) and invasive front (IF) and found that TB were infiltrated with CD3$^+$, CD4$^+$, and CD8$^+$ cells—but mostly accumulated in the IF—while ROCKi

(D and E) Cell-cycle analysis after treatment (n = 3–4). Sensitive (s)-resistant (R) pairs (D, left A375; right WM983A). A375/PLX/R (E); G, GSK269962A; B, blebbistatin.

(F) p-STAT3 levels after treatment (n = 3 patient no. 35, WM793B; n = 4 A375/PLX/R; n = 5 patients no. 2 and 58).

(G) Mcl-1 levels of A375/PLX/R cells after treatment (n = 3).

(H and I) Percentage of dead cells by annexin V/PI staining of A375/PLX/R (H), patient no. 2 and no. 62T3 (I) cells after a 3-day treatment (n = 4 A375/PLX/R; n = 5 patient no. 2; n = 4 patient no. 62T3).

(C, D, and F–I) ROCKi GSK269962A; (E, H, and I) BRAFi PLX4720.

(C–G) 24 h treatment.

(C and F–I) Mean ± SEM and individual data points (circle). Asterisks in (D and E) are statistical significance in multinucleated cells. p values by one-way ANOVA with Tukey's (D–F, H, and I) or Benjamini, Krieger, and Yekutieli correction (C); unpaired t test with Welch's correction (G), *$p < 0.05$, **$p < 0.01$, ***$p < 0.001$, ****$p < 0.0001$; n.s., not significant. See also Figure S6.

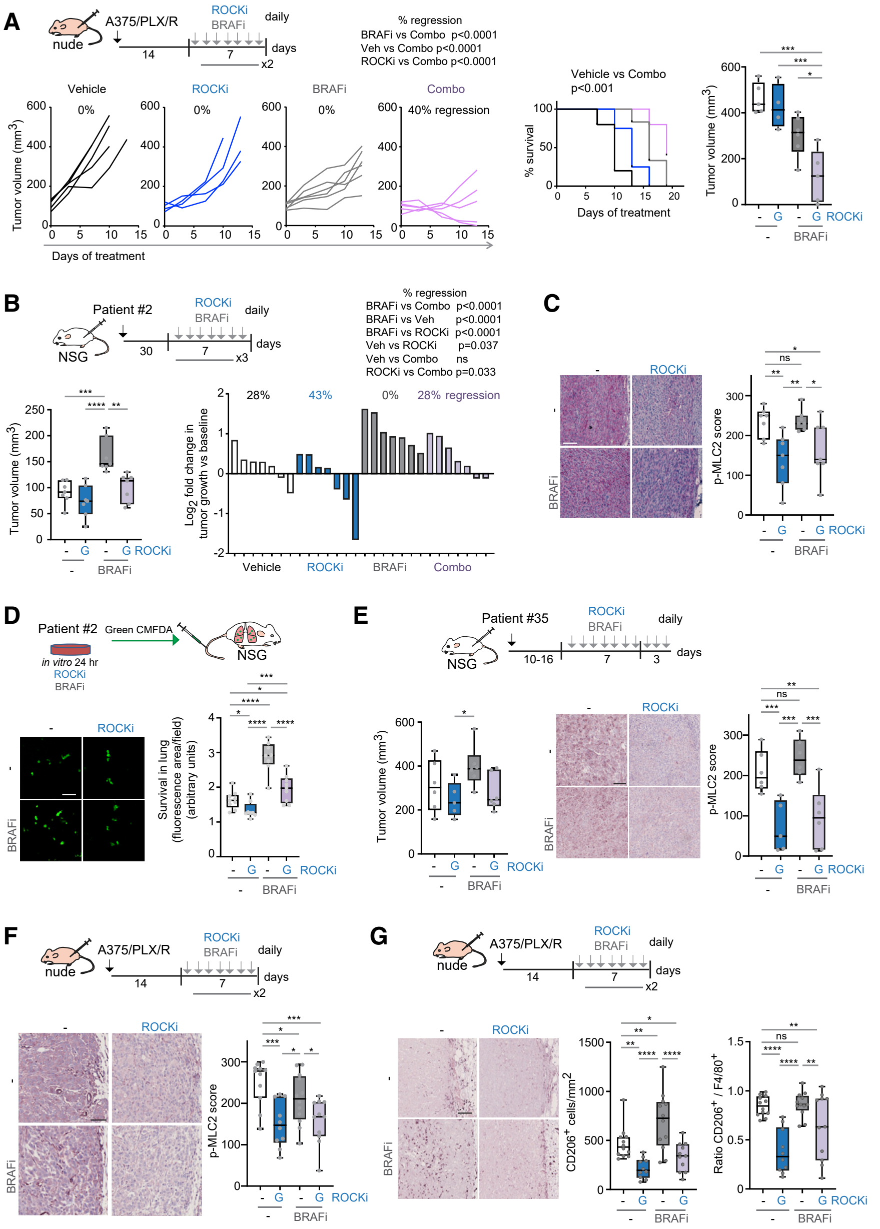

Figure 7. Combining ROCK Inhibitors with BRAF Inhibitors *In Vivo*

(A) Top, schematic of experiment. Left, growth of A375/PLX/R xenografts in nude mice after treatment. Middle, Kaplan-Meier survival plot. Right, tumor volume at endpoint (n = 4–6 mice/group).

(B) Left, volume of patient no. 2 xenografts in NSG mice after a 21-day treatment (n = 7 mice/group). Right, tumor growth at endpoint versus baseline.

(C) p-MLC2 staining in patient no. 2 xenografts. Scale bar, 100 μm.

(D) Survival of patient no. 2 cells in the mouse lung 24 h post-injection (n = 8–9 mice from 2 experiments). Scale bar, 100 μm.

(E) Left, volume of patient no. 35 xenografts in NSG mice after a 10-day treatment (n = 6 mice/group). Right, p-MLC2 staining. Scale bar, 100 μm.

(F and G) Images and quantification of p-MLC2 (F), CD206$^+$ (G) in A375/PLX/R xenografts from (A). Scale bars, 100 μm. Ratio of CD206$^+$/F4/80$^+$ shown. (F and G) Pooled data from 2 experiments.

(A–G) ROCKi GSK269962A, BRAFi PLX4720.

Boxplots show median (center line); interquartile range (box); min-max (whiskers); and individual mice (circles). p values by ANOVA with Tukey's: (A) right graph, (B) left graph; Benjamini, Krieger, and Yekutieli (C, D, F, G, and E, right) or Dunnett's correction (E, left), Mantel-Cox (A, survival plot), chi-square test: percentage regressions in (A, left) and (B, right). *p < 0.05, **p < 0.01, ***p < 0.001, ****p < 0.0001; n.s., not significant. See also Figure S7.

did not alter distribution (Figure S8G). Moreover, ROCKi did not affect viability of CD8$^+$ T cells or tumor-killing ability *in vitro* (data not shown). Therefore, ROCKi does not affect CD4$^+$ and CD8$^+$ cell functions tested.

We next analyzed anti-PD-1/NR and Resp tumors (Figures 8E and 8F). NR had increased levels of p-MLC2 and CD206$^+$ cells compared with Resp while on anti-PD-1 treatment (Figure 8F, middle). FOXP3$^+$ Tregs did not change (Figure 8F, right). NR tumors polarized most macrophages into CD206$^+$ compared with less polarization in Resp (Figure 8F, right) and in parental 5555 (Figure 8C). These data could in part explain the lack of response to anti-PD-1 (Figure 8F, left).

Then an anti-PD-1/NR (intrinsic resistance) was allografted into new recipient mice that were treated with anti-PD-1 twice a week post-injection to maintain resistance *in vivo* (Figures 8E, 8G, and S8H). After 7 days, we treated with anti-PD-1, ROCKi, or both. Tumors on anti-PD-1 grew rapidly but combo therapy resulted in >40% regression of established tumors and improved survival (Figures 8G and S8H). Treatments were tolerated (Figure S8I) and ROCKi reduced p-MLC2 (Figure 8H, left). Importantly, combo decreased expression of immune checkpoint ligand PD-L1 on tumor cells (Figure 8H, right). Anti-PD-1/NR tumors polarized most macrophages into CD206$^+$ phenotype (Figure S8J) and combo decreased expression of PD-L1 on CD206$^+$ macrophages (Figure 8I, left), while total macrophage content did not change (Figure S8K). Finally, combo decreased Tregs (Figure 8I, right), while other immune populations did not change (Figure S8K).

ROCK-myosin II regulates TGF-β secretion from amoeboid melanoma cells (Cantelli et al., 2015). TGF-β is a potent immunosuppressor that induces Tregs and myeloid-derived suppressor cells (Cantelli et al., 2017; Condamine et al., 2015; Nakamura et al., 2001). Therefore, ROCKi decreased TGF-β1 levels secreted by immunotherapy-resistant patient-derived cell lines and by 5555 cells (Figure S8L). Interleukin-6, CCL2, TGF-β1, and colony-stimulating factor 1/macrophage colony-stimulating factor immunomodulatory cytokines (Fisher et al., 2014; Mantovani et al., 2004; Qian et al., 2011; Roca et al., 2009) regulated by

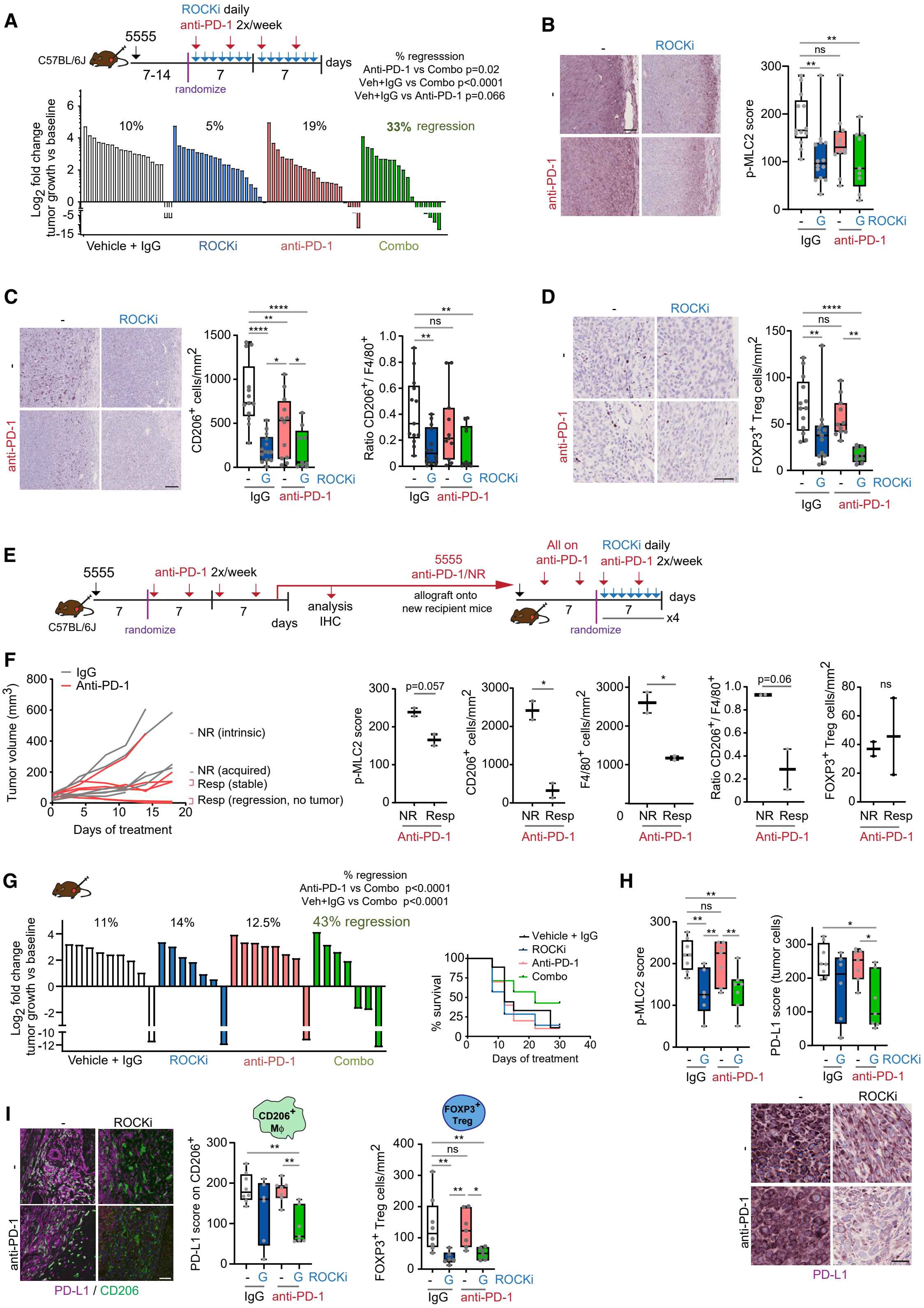

Figure 8. ROCK-Myosin II Inhibition Improves Efficacy of Immune Checkpoint Inhibitors

(A) Top, schematic of treatment. Bottom, growth of 5555 allografts in C57BL/6J mice after treatment. Pooled data from 3 experiments (n = 6–8 mice/group/experiment).

(legend continued on next page)

ROCK-myosin II (Georgouli et al., 2019; Le Dreau et al., 2010) were upregulated in group 1 MAPKi-resistant melanomas (Figure S8M). Therefore, blocking ROCK-myosin II reduces immunosuppressive microenvironments, improving anti-PD-1 action on pre-existing T cells (Mariathasan et al., 2018; Tauriello et al., 2018).

DISCUSSION

Recurrent transcriptional alterations occur during development of resistance to MAPKi (Song et al., 2017). In this study we find that adaptation to therapy occurs early on treatment through cytoskeletal remodeling leading to restoration/increase of myosin II levels in resistant melanomas. Because targeted and immunotherapy-resistant cells rely on ROCK-dependent myosin II for survival, this could be a key mediator of cross-resistance. Resistant melanomas increase either MLC2 expression and/or activity, which in turn increases and reinforces myosin II activity (Calvo et al., 2013; Medjkane et al., 2009). Cells under drug treatment upregulate myosin II as a pro-survival response to MAPK inhibition, resulting in uncoupling of ERK signals to the cytoskeleton.

Although myosin II activity is controlled by BRN2-mediated downregulation of PDE5A and increased calcium signaling in BRAF mutant melanoma (Arozarena et al., 2011), our mechanism seems operative in NRAS mutant melanoma. PDE5A expression increases in MAPKi-resistant lines compared with parental (Song et al., 2017) in a similar fashion as MLC2 (*MYL9*) (data not shown). Because p-MLC2 levels are restored/increased in resistant versus parental lines, there may be mechanisms blocking the inhibitory action of PDE5A on myosin II in resistant cells. Moreover, myosin II levels are ROCK dependent in resistant cells, so PDE5A may not regulate myosin II activity in this context.

MAPKi-resistant cells have been associated to bundled collagen and pro-survival signals (Brighton et al., 2018). Increased ECM deposition found in resistant tumors could contribute to myosin II activity *in vivo* (Laklai et al., 2016; Paszek et al., 2005). Likewise, ROCK-myosin II-driven contractility also induces ECM stiffening (Samuel et al., 2011), generating a feedback loop between myosin II and ECM.

Widely studied in cell migration (Jaffe and Hall, 2005; Olson, 2008; Sadok et al., 2015; Sahai and Marshall, 2002; Sanz-Moreno et al., 2008), ROCK-myosin II is proposed here as a therapeutic target that goes beyond this pro-migratory function. We show how this machinery controls intrinsic survival and extrinsic immunosuppression. Importantly, contractile cytoskeletal features are observed in metastatic lesions compared with primary tumors (Cantelli et al., 2015; Herraiz et al., 2015; Orgaz et al., 2014b; Sanz-Moreno et al., 2011), which suggests that metastatic traits can be linked to drug resistance (Alexander and Friedl, 2012). Pathways controlling invasion and metastasis are aberrantly activated by non-mutational mechanisms—overexpression or signaling alteration (Alexander and Friedl, 2012; Orgaz et al., 2014a)—in contrast with frequently mutated MAPK (Davies et al., 2002; Cancer Genome Atlas Network, 2015). Rho GTPases are overexpressed in cancer (Orgaz et al., 2014a); particularly RhoC is a driver of melanoma metastasis by increased expression (Clark et al., 2000). Lower frequency of mutations suggests that cancer cells are less addicted to these pathways and, upon inhibition, development of resistance could be less frequent. Although we have shown that myosin II inhibition also impairs survival of therapy-sensitive melanoma cells, therapy-resistant cells are more sensitive to ROCKi. This is due to resistant cells having gained certain survival traits, but acquired vulnerabilities in return, such as defective anti-oxidant and DNA damage repair responses.

Inhibition of myosin II activity overcomes resistance in melanoma through induction of lethal ROS, unresolved DNA damage, and loss of pro-survival signaling, which leads to cell-cycle arrest and cell death. A recent study has described that HDAC inhibitors (HDACi) also induce lethal ROS and DNA damage in MAPKi-resistant melanomas (Wang et al., 2018). It will be important to investigate if/how HDACi regulate cytoskeletal remodeling.

The tumor microenvironment has a key role in resistance to therapies in melanoma (Almeida et al., 2019) and macrophages can contribute to resistance to MAPKi through secretion of pro-survival factors (Smith et al., 2014). Furthermore, TGF-β inhibition enhanced efficacy of immune checkpoint inhibitors (Mariathasan et al., 2018; Tauriello et al., 2018). In addition to the cell intrinsic effects we observe, we report how inhibition of ROCK-myosin II reduces pro-tumorigenic CD206$^+$ macrophages, which could contribute to reducing tumor growth. Moreover, ROCK-myosin II inhibition decreases FOXP3$^+$ Tregs. Combination of ROCKi with anti-PD-1 also reduces PD-L1 expression on both tumor cells and CD206$^+$ macrophages. These effects could be due to lower STAT3 activity after ROCK inhibition (Sanz-Moreno et al., 2011), since PD-L1 expression can be regulated by STAT3 (Marzec et al., 2008; Pardoll, 2012). Effects on T cells are likely due to ROCK-myosin II regulation of TGF-β in cancer cells (Cantelli et al., 2015). Decreased TGF-β production by melanoma induced by ROCKi can contribute to improved anti-PD-1 responses.

(B–D) Images and quantification of p-MLC2 (B), CD206$^+$ (C), and FOXP3$^+$ (D) cells in 5555 tumors at endpoint (pooled data from 2 experiments). Ratio CD206$^+$/F4/80$^+$ shown. Scale bars, 100 μm (p-MLC2, CD206) and 50 μm (FOXP3).

(E) Schematic of experiment.

(F) Left, growth of 5555 allografts after treatment. Right, quantification of p-MLC2, CD206$^+$, F4/80$^+$, and ratio CD206$^+$/F4/80$^+$ in anti-PD-1/NR or Resp tumors.

(G) Left, growth of 5555 anti-PD-1/NR allografts in new recipient mice after treatment (n = 7-8 mice/group). Right, survival plot.

(H) Images and quantification of p-MLC2 and PD-L1 on tumor cells in tumors from (G). Scale bars, 25 μm.

(I) Left, images and quantification of PD-L1 on CD206$^+$ cells in tumors from (G). Images show merged pseudo-colors for each staining. Scale bar, 50 μm. Right, quantification of FOXP3$^+$ Tregs in tumors from (G).

(A–D and G–I) ROCKi GSK269962A.

Boxplots show median (center line); interquartile range (box); min-max (whiskers); and individual mice (circles). p values by ANOVA with Benjamini, Krieger, and Yekutieli correction (B–D and H–I), t test (F), chi-square test: percentage regressions in (A) and (G). *$p < 0.05$, **$p < 0.01$, ***$p < 0.001$, ****$p < 0.0001$; n.s., not significant. See also Figure S8.

ROCKi Fasudil has been used safely in Japan since 1995 to treat subarachnoid hemorrhage (SAH) after a head trauma and to prevent vasospasm associated with SAH (Feng et al., 2016; Olson, 2008). ROCKi is given as a vasodilator to lower blood pressure (Olson, 2008), and Fasudil and other ROCKi are being tested in clinical trials for glaucoma and other vascular diseases, such as pulmonary hypertension and atherosclerosis (Olson, 2008). Optimal ROCKi could be tested in broader range of disease, as a strategy to extend clinical response to different cancer therapies or even as a single therapy in the case of drug-addicted tumors. Importantly, therapy-resistant cells are more sensitive to ROCKi while its combination with current therapies seems to elicit a superior response. Lower doses of ROCKi and/or different schedule treatments could be used in combination with current therapies to prolong their efficacy and delay resistance. Alternatively, different delivery strategies of ROCKi (local, antibody-drug conjugate) could be considered.

In summary, we provide extensive evidence that targeting cytoskeletal regulators driving high myosin II activity overcomes resistance to targeted and immunotherapies in melanoma. The cytoskeletal adaptations that occur very early on treatment provide not only a survival advantage but also a vulnerability, which can be later exploited. High myosin II activity identifies therapy cross-resistant patients, suggesting its potential as a biomarker. Our work opens the possibility that cytoskeletal remodeling could be a conserved pro-survival mechanism of generating therapy-resistant cancer clones under the selection of other therapy regimes.

STAR★METHODS

Detailed methods are provided in the online version of this paper and include the following:

- KEY RESOURCES TABLE
- LEAD CONTACT AND MATERIALS AVAILABILITY
- EXPERIMENTAL MODEL AND SUBJECT DETAILS
  - Patient-Derived Samples
  - Cell Lines and Patient-Derived Cell Lines
  - Animals
- METHOD DETAILS
  - Chemicals
  - Antibodies
  - Analysis of Cell Morphology
  - Long-Term Survival
  - Long Term Survival on Collagen I Matrices
  - MTT Assay
  - Cell Cycle Analysis
  - AnnexinV/Propidium Iodide FACS
  - ROS Detection
  - Time Lapse Microscopy
  - RNAi
  - MLC2 Rescue Experiments
  - MLC2 Stable Overexpression
  - Immunofluorescence and Confocal Imaging
  - Immunoblotting
  - TGF-β1 ELISA
  - Phospho-proteomics
  - Phospho-Peptide Enrichment Analysis
  - Quantitative Real Time One-Step PCR
  - Gene Expression Studies and Analysis
  - Gene Enrichment Analyzes
  - Tumor Xenografts
  - Immunotherapy Experiments
  - Survival in the Lung Assay
  - Immunohistochemistry
  - Imaging and Scoring
- QUANTIFICATION AND STATISTICAL ANALYSIS
- DATA AND CODE AVAILABILITY

SUPPLEMENTAL INFORMATION

Supplemental Information can be found online at https://doi.org/10.1016/j.ccell.2019.12.003.

ACKNOWLEDGMENTS

The work was supported by Cancer Research UK (CRUK) C33043/A12065 (to V.S.-M., J.L.O., and E.C.-M.), C33043/A24478 (to V.S.-M., E.C.-M., M.G., and J.L.O.); C30122/A11527, C30122/A15774, C33043/A12065 (to S.N.K.); C107/A10433, C107/A104339 (to A.S.); The Harry J. Lloyd Charitable Trust (to J.L.O. and V.S.-M.); Barts Charity (to V.S.-M., I.R.-H., and J.M.); Royal Society RG110591 (to V.S.-M.); Fundación Ramón Areces (to E.C.-M.); Marie Sklodowska-Curie Action (H2020-MSCA-IF-2014-EF-ST) (to I.R.-H.); MR/L023091/1 (to S.N.K. and S.M.); CRUK/NIHR in England/DoH for Scotland, Wales and Northern Ireland ECMC (C10355/A15587) (to S.N.K.); Francis Crick Institute core funding from CRUK (FC001112), MRC (FC001112), and Wellcome Trust (FC001112) (to I.M. and A.P.); NIHR Biomedical Research Centre (BRC) at Guy's and St Thomas' NHS Foundation Trust and King's College London, IS-BRC-1215-20006 (to S.N.K.). Fluorescence-activated cell sorting was performed at BRC funded by NIHR. We are indebted to Richard Marais and his team (CRUK Manchester Institute) for kind provision of cell lines and Paul Lorigan (University of Manchester) for providing patient samples (study approved by Manchester Cancer Research Center Biobank Access Committee application 13_RIMA_01; the role of the MCRC Biobank is to distribute samples and cannot endorse studies performed or interpretation of results). We thank Fran Balkwill, Colin Pegrum, and the Biological Services Unit at Barts Cancer Institute for help with mouse work; Romina Girotti and Jeremy Carlton for helpful discussions; Fernando Calvo for MLC2 plasmids and advice on ssGSEA; Amaya Viros, Garry Ashton, Sandra Kümper, and Michela Perani for technical advice; Erik Sahai and Tohru Takaki for MLC2 lentivectors; Øystein Fodstad for LOX-IMVI cells; and Wellcome Trust Functional Genomics Cell Bank for MM485 cells.

AUTHOR CONTRIBUTIONS

Conceptualization, V.S.-M. and J.L.O.; Methodology, J.L.O., A.P.-R., R.L., I.M., V.S.-M., and F.W.; Investigation, J.L.O., E.C.-M., A.S., O.M., I.R.-H., A.P.-R., J.M., V.B., M.G., P.P., L.B., S.M., P.K., C.T., and F.W.; Validation, J.L.O., E.C.-M., I.R.-H., A.P.-R., and O.M.; Writing – Original Draft, V.S.-M. and J.L.O.; Writing – Review & Editing, V.S.-M., J.L.O., E.C.-M., and I.M.; Funding Acquisition, V.S.-M., J.L.O., I.M., and S.N.K.; Resources, I.M., S.N.K., and R.L.; Supervision, V.S.-M.

DECLARATION OF INTERESTS

The authors declare no competing interests.

Received: January 9, 2018
Revised: September 4, 2019
Accepted: December 6, 2019
Published: January 13, 2020

REFERENCES

Alexander, S., and Friedl, P. (2012). Cancer invasion and resistance: interconnected processes of disease progression and therapy failure. Trends Mol. Med. *18*, 13–26.

Almeida, F.V., Douglass, S.M., Fane, M.E., and Weeraratna, A.T. (2019). Bad company: microenvironmentally mediated resistance to targeted therapy in melanoma. Pigment Cell Melanoma Res. *32*, 237–247.

Arozarena, I., Sanchez-Laorden, B., Packer, L., Hidalgo-Carcedo, C., Hayward, R., Viros, A., Sahai, E., and Marais, R. (2011). Oncogenic BRAF induces melanoma cell invasion by downregulating the cGMP-specific phosphodiesterase PDE5A. Cancer Cell *19*, 45–57.

Baenke, F., Chaneton, B., Smith, M., Van Den Broek, N., Hogan, K., Tang, H., Viros, A., Martin, M., Galbraith, L., Girotti, M.R., et al. (2015). Resistance to BRAF inhibitors induces glutamine dependency in melanoma cells. Mol. Oncol. *10*, 73–84.

Balch, C.M., Gershenwald, J.E., Soong, S.J., Thompson, J.F., Atkins, M.B., Byrd, D.R., Buzaid, A.C., Cochran, A.J., Coit, D.G., Ding, S., et al. (2009). Final version of 2009 AJCC melanoma staging and classification. J. Clin. Oncol. *27*, 6199–6206.

Bankhead, P., Loughrey, M.B., Fernandez, J.A., Dombrowski, Y., McArt, D.G., Dunne, P.D., McQuaid, S., Gray, R.T., Murray, L.J., Coleman, H.G., et al. (2017). QuPath: open source software for digital pathology image analysis. Sci. Rep. *7*, 16878.

Brighton, H.E., Angus, S.P., Bo, T., Roques, J., Tagliatela, A.C., Darr, D.B., Karagoz, K., Sciaky, N., Gatza, M.L., Sharpless, N.E., et al. (2018). New mechanisms of resistance to MEK inhibitors in melanoma revealed by intravital imaging. Cancer Res. *78*, 542–557.

Calvo, F., Sanz-Moreno, V., Agudo-Ibanez, L., Wallberg, F., Sahai, E., Marshall, C.J., and Crespo, P. (2011). RasGRF suppresses Cdc42-mediated tumour cell movement, cytoskeletal dynamics and transformation. Nat. Cell Biol. *13*, 819–826.

Calvo, F., Ege, N., Grande-Garcia, A., Hooper, S., Jenkins, R.P., Chaudhry, S.I., Harrington, K., Williamson, P., Moeendarbary, E., Charras, G., and Sahai, E. (2013). Mechanotransduction and YAP-dependent matrix remodelling is required for the generation and maintenance of cancer-associated fibroblasts. Nat. Cell Biol. *15*, 637–646.

Cancer Genome Atlas Network. (2015). Genomic classification of cutaneous melanoma. Cell *161*, 1681–1696.

Cantelli, G., Orgaz, J.L., Rodriguez-Hernandez, I., Karagiannis, P., Maiques, O., Matias-Guiu, X., Nestle, F.O., Marti, R.M., Karagiannis, S.N., and Sanz-Moreno, V. (2015). TGF-b-induced transcription sustains amoeboid melanoma migration and dissemination. Curr. Biol. *25*, 2899–2914.

Cantelli, G., Crosas-Molist, E., Georgouli, M., and Sanz-Moreno, V. (2017). TGFBeta-induced transcription in cancer. Semin. Cancer Biol. *42*, 60–69.

Chapman, P.B., Hauschild, A., Robert, C., Haanen, J.B., Ascierto, P., Larkin, J., Dummer, R., Garbe, C., Testori, A., Maio, M., et al. (2011). Improved survival with vemurafenib in melanoma with BRAF V600E mutation. N. Engl. J. Med. *364*, 2507–2516.

Clark, E.A., Golub, T.R., Lander, E.S., and Hynes, R.O. (2000). Genomic analysis of metastasis reveals an essential role for RhoC. Nature *406*, 532–535.

Condamine, T., Ramachandran, I., Youn, J.I., and Gabrilovich, D.I. (2015). Regulation of tumor metastasis by myeloid-derived suppressor cells. Annu. Rev. Med. *66*, 97–110.

Davies, H., Bignell, G.R., Cox, C., Stephens, P., Edkins, S., Clegg, S., Teague, J., Woffendin, H., Garnett, M.J., Bottomley, W., et al. (2002). Mutations of the BRAF gene in human cancer. Nature *417*, 949–954.

Dhomen, N., Reis-Filho, J.S., da Rocha Dias, S., Hayward, R., Savage, K., Delmas, V., Larue, L., Pritchard, C., and Marais, R. (2009). Oncogenic Braf induces melanocyte senescence and melanoma in mice. Cancer Cell *15*, 294–303.

Le Dreau, G., Kular, L., Nicot, A.B., Calmel, C., Melik-Parsadaniantz, S., Kitabgi, P., Laurent, M., and Martinerie, C. (2010). NOV/CCN3 upregulates CCL2 and CXCL1 expression in astrocytes through beta1 and beta5 integrins. Glia *58*, 1510–1521.

Feng, Y., LoGrasso, P.V., Defert, O., and Li, R. (2016). Rho kinase (ROCK) inhibitors and their therapeutic potential. J. Med. Chem. *59*, 2269–2300.

Fisher, D.T., Appenheimer, M.M., and Evans, S.S. (2014). The two faces of IL-6 in the tumor microenvironment. Semin. Immunol. *26*, 38–47.

Flaherty, K.T., Puzanov, I., Kim, K.B., Ribas, A., McArthur, G.A., Sosman, J.A., O'Dwyer, P.J., Lee, R.J., Grippo, J.F., Nolop, K., and Chapman, P.B. (2010). Inhibition of mutated, activated BRAF in metastatic melanoma. N. Engl. J. Med. *363*, 809–819.

Flaherty, K.T., Infante, J.R., Daud, A., Gonzalez, R., Kefford, R.F., Sosman, J., Hamid, O., Schuchter, L., Cebon, J., Ibrahim, N., et al. (2012). Combined BRAF and MEK inhibition in melanoma with BRAF V600 mutations. N. Engl. J. Med. *367*, 1694–1703.

Gadea, G., Sanz-Moreno, V., Self, A., Godi, A., and Marshall, C.J. (2008). DOCK10-mediated Cdc42 activation is necessary for amoeboid invasion of melanoma cells. Curr. Biol. *18*, 1456–1465.

Georgouli, M., Herraiz, C., Crosas-Molist, E., Fanshawe, B., Maiques, O., Perdrix, A., Pandya, P., Rodriguez-Hernandez, I., Ilieva, K.M., Cantelli, G., et al. (2019). Regional activation of myosin II in cancer cells drives tumor progression via a secretory cross-talk with the immune microenvironment. Cell *176*, 757–774.e23.

Girotti, M.R., Pedersen, M., Sanchez-Laorden, B., Viros, A., Turajlic, S., Niculescu-Duvaz, D., Zambon, A., Sinclair, J., Hayes, A., Gore, M., et al. (2013). Inhibiting EGF receptor or SRC family kinase signaling overcomes BRAF inhibitor resistance in melanoma. Cancer Discov. *3*, 158–167.

Gray-Schopfer, V., Wellbrock, C., and Marais, R. (2007). Melanoma biology and new targeted therapy. Nature *445*, 851–857.

Hall, A. (2012). Rho family GTPases. Biochem. Soc. Trans. *40*, 1378–1382.

Haystead, T.A. (2005). ZIP kinase, a key regulator of myosin protein phosphatase 1. Cell Signal. *17*, 1313–1322.

Herraiz, C., Calvo, F., Pandya, P., Cantelli, G., Rodriguez-Hernandez, I., Orgaz, J.L., Kang, N., Chu, T., Sahai, E., and Sanz-Moreno, V. (2015). Reactivation of p53 by a cytoskeletal sensor to control the balance between DNA damage and tumor dissemination. J. Natl. Cancer Inst. *108*, https://doi.org/10.1093/jnci/djv289.

Hirata, E., Girotti, M.R., Viros, A., Hooper, S., Spencer-Dene, B., Matsuda, M., Larkin, J., Marais, R., and Sahai, E. (2015). Intravital imaging reveals how BRAF inhibition generates drug-tolerant microenvironments with high integrin beta1/FAK signaling. Cancer Cell *27*, 574–588.

Hodi, F.S., O'Day, S.J., McDermott, D.F., Weber, R.W., Sosman, J.A., Haanen, J.B., Gonzalez, R., Robert, C., Schadendorf, D., Hassel, J.C., et al. (2010). Improved survival with ipilimumab in patients with metastatic melanoma. N. Engl. J. Med. *363*, 711–723.

Hong, A., Moriceau, G., Sun, L., Lomeli, S., Piva, M., Damoiseaux, R., Holmen, S.L., Sharpless, N.E., Hugo, W., and Lo, R.S. (2017). Exploiting drug addiction mechanisms to select against MAPKi-resistant melanoma. Cancer Discov. *8*, 74–93.

Hugo, W., Shi, H., Sun, L., Piva, M., Song, C., Kong, X., Moriceau, G., Hong, A., Dahlman, K.B., Johnson, D.B., et al. (2015). Non-genomic and immune evolution of melanoma acquiring MAPKi resistance. Cell *162*, 1271–1285.

Hugo, W., Zaretsky, J.M., Sun, L., Song, C., Moreno, B.H., Hu-Lieskovan, S., Berent-Maoz, B., Pang, J., Chmielowski, B., Cherry, G., et al. (2016). Genomic and transcriptomic features of response to anti-PD-1 therapy in metastatic melanoma. Cell *165*, 35–44.

Ito, M., Nakano, T., Erdodi, F., and Hartshorne, D.J. (2004). Myosin phosphatase: structure, regulation and function. Mol. Cell. Biochem. *259*, 197–209.

Itoh, K., Yoshioka, K., Akedo, H., Uehata, M., Ishizaki, T., and Narumiya, S. (1999). An essential part for Rho-associated kinase in the transcellular invasion of tumor cells. Nat. Med. *5*, 221–225.

Jaffe, A.B., and Hall, A. (2005). Rho GTPases: biochemistry and biology. Annu. Rev. Cell Dev. Biol. *21*, 247–269.

Kakavand, H., Rawson, R.V., Pupo, G.M., Yang, J.Y.H., Menzies, A.M., Carlino, M.S., Kefford, R.F., Howle, J.R., Saw, R.P.M., Thompson, J.F., et al.

(2017). PD-L1 expression and immune escape in melanoma resistance to MAPK inhibitors. Clin. Cancer Res. *23*, 6054–6061.

Kong, X., Kuilman, T., Shahrabi, A., Boshuizen, J., Kemper, K., Song, J.Y., Niessen, H.W.M., Rozeman, E.A., Geukes Foppen, M.H., Blank, C.U., et al. (2017). Cancer drug addiction is relayed by an ERK2-dependent phenotype switch. Nature *550*, 270–274.

Konieczkowski, D.J., Johannessen, C.M., Abudayyeh, O., Kim, J.W., Cooper, Z.A., Piris, A., Frederick, D.T., Barzily-Rokni, M., Straussman, R., Haq, R., et al. (2014). A melanoma cell state distinction influences sensitivity to MAPK pathway inhibitors. Cancer Discov. *4*, 816–827.

Konieczkowski, D.J., Johannessen, C.M., and Garraway, L.A. (2018). A convergence-based framework for cancer drug resistance. Cancer Cell *33*, 801–815.

Krokan, H.E., and Bjoras, M. (2013). Base excision repair. Cold Spring Harb. Perspect. Biol. *5*, a012583.

Kumper, S., Mardakheh, F.K., McCarthy, A., Yeo, M., Stamp, G.W., Paul, A., Worboys, J., Sadok, A., Jorgensen, C., Guichard, S., and Marshall, C.J. (2016). Rho-associated kinase (ROCK) function is essential for cell cycle progression, senescence and tumorigenesis. Elife *5*, e12994.

Kwong, L.N., Boland, G.M., Frederick, D.T., Helms, T.L., Akid, A.T., Miller, J.P., Jiang, S., Cooper, Z.A., Song, X., Seth, S., et al. (2015). Co-clinical assessment identifies patterns of BRAF inhibitor resistance in melanoma. J. Clin. Invest. *125*, 1459–1470.

Laklai, H., Miroshnikova, Y.A., Pickup, M.W., Collisson, E.A., Kim, G.E., Barrett, A.S., Hill, R.C., Lakins, J.N., Schlaepfer, D.D., Mouw, J.K., et al. (2016). Genotype tunes pancreatic ductal adenocarcinoma tissue tension to induce matricellular fibrosis and tumor progression. Nat. Med. *22*, 497–505.

Lammermann, T., and Sixt, M. (2009). Mechanical modes of 'amoeboid' cell migration. Curr. Opin. Cell Biol. *21*, 636–644.

Larkin, J., Ascierto, P.A., Dreno, B., Atkinson, V., Liszkay, G., Maio, M., Mandala, M., Demidov, L., Stroyakovskiy, D., Thomas, L., et al. (2014). Combined vemurafenib and cobimetinib in BRAF-mutated melanoma. N. Engl. J. Med. *371*, 1867–1876.

Larkin, J., Chiarion-Sileni, V., Gonzalez, R., Grob, J.J., Cowey, C.L., Lao, C.D., Schadendorf, D., Dummer, R., Smylie, M., Rutkowski, P., et al. (2015). Combined nivolumab and ipilimumab or monotherapy in untreated melanoma. N. Engl. J. Med. *373*, 23–34.

Lau, P.K., Ascierto, P.A., and McArthur, G. (2016). Melanoma: the intersection of molecular targeted therapy and immune checkpoint inhibition. Curr. Opin. Immunol. *39*, 30–38.

Lito, P., Pratilas, C.A., Joseph, E.W., Tadi, M., Halilovic, E., Zubrowski, M., Huang, A., Wong, W.L., Callahan, M.K., Merghoub, T., et al. (2012). Relief of profound feedback inhibition of mitogenic signaling by RAF inhibitors attenuates their activity in BRAFV600E melanomas. Cancer Cell *22*, 668–682.

Long, G.V., Fung, C., Menzies, A.M., Pupo, G.M., Carlino, M.S., Hyman, J., Shahheydari, H., Tembe, V., Thompson, J.F., Saw, R.P., et al. (2014a). Increased MAPK reactivation in early resistance to dabrafenib/trametinib combination therapy of BRAF-mutant metastatic melanoma. Nat. Commun. *5*, 5694.

Long, G.V., Stroyakovskiy, D., Gogas, H., Levchenko, E., de Braud, F., Larkin, J., Garbe, C., Jouary, T., Hauschild, A., Grob, J.J., et al. (2014b). Combined BRAF and MEK inhibition versus BRAF inhibition alone in melanoma. N. Engl. J. Med. *371*, 1877–1888.

Lu, H., Liu, S., Zhang, G., Bin, W., Zhu, Y., Frederick, D.T., Hu, Y., Zhong, W., Randell, S., Sadek, N., et al. (2017). PAK signalling drives acquired drug resistance to MAPK inhibitors in BRAF-mutant melanomas. Nature *550*, 133–136.

Mantovani, A., Sica, A., Sozzani, S., Allavena, P., Vecchi, A., and Locati, M. (2004). The chemokine system in diverse forms of macrophage activation and polarization. Trends Immunol. *25*, 677–686.

Mariathasan, S., Turley, S.J., Nickles, D., Castiglioni, A., Yuen, K., Wang, Y., Kadel, E.E., III, Koeppen, H., Astarita, J.L., Cubas, R., et al. (2018). TGFbeta attenuates tumour response to PD-L1 blockade by contributing to exclusion of T cells. Nature *554*, 544–548.

Marzec, M., Zhang, Q., Goradia, A., Raghunath, P.N., Liu, X., Paessler, M., Wang, H.Y., Wysocka, M., Cheng, M., Ruggeri, B.A., and Wasik, M.A. (2008). Oncogenic kinase NPM/ALK induces through STAT3 expression of immunosuppressive protein CD274 (PD-L1, B7-H1). Proc. Natl. Acad. Sci. U S A *105*, 20852–20857.

Medjkane, S., Perez-Sanchez, C., Gaggioli, C., Sahai, E., and Treisman, R. (2009). Myocardin-related transcription factors and SRF are required for cytoskeletal dynamics and experimental metastasis. Nat. Cell Biol. *11*, 257–268.

Mjelle, R., Hegre, S.A., Aas, P.A., Slupphaug, G., Drablos, F., Saetrom, P., and Krokan, H.E. (2015). Cell cycle regulation of human DNA repair and chromatin remodeling genes. DNA Repair (Amst.) *30*, 53–67.

Moriceau, G., Hugo, W., Hong, A., Shi, H., Kong, X., Yu, C.C., Koya, R.C., Samatar, A.A., Khanlou, N., Braun, J., et al. (2015). Tunable-combinatorial mechanisms of acquired resistance limit the efficacy of BRAF/MEK cotargeting but result in melanoma drug addiction. Cancer Cell *27*, 240–256.

Nakamura, K., Kitani, A., and Strober, W. (2001). Cell contact-dependent immunosuppression by CD4(+)CD25(+) regulatory T cells is mediated by cell surface-bound transforming growth factor beta. J. Exp. Med. *194*, 629–644.

Obenauf, A.C., Zou, Y., Ji, A.L., Vanharanta, S., Shu, W., Shi, H., Kong, X., Bosenberg, M.C., Wiesner, T., Rosen, N., et al. (2015). Therapy-induced tumour secretomes promote resistance and tumour progression. Nature *520*, 368–372.

Olson, M.F. (2008). Applications for ROCK kinase inhibition. Curr. Opin. Cell Biol. *20*, 242–248.

Orgaz, J.L., Herraiz, C., and Sanz-Moreno, V. (2014a). Rho GTPases modulate malignant transformation of tumor cells. Small GTPases *5*, e29019.

Orgaz, J.L., Pandya, P., Dalmeida, R., Karagiannis, P., Sanchez-Laorden, B., Viros, A., Albrengues, J., Nestle, F.O., Ridley, A.J., Gaggioli, C., et al. (2014b). Diverse matrix metalloproteinase functions regulate cancer amoeboid migration. Nat. Commun. *5*, 4255.

Pardoll, D.M. (2012). The blockade of immune checkpoints in cancer immunotherapy. Nat. Rev. Cancer *12*, 252–264.

Paszek, M.J., Zahir, N., Johnson, K.R., Lakins, J.N., Rozenberg, G.I., Gefen, A., Reinhart-King, C.A., Margulies, S.S., Dembo, M., Boettiger, D., et al. (2005). Tensional homeostasis and the malignant phenotype. Cancer Cell *8*, 241–254.

Peng, G., Chun-Jen Lin, C., Mo, W., Dai, H., Park, Y.Y., Kim, S.M., Peng, Y., Mo, Q., Siwko, S., Hu, R., et al. (2014). Genome-wide transcriptome profiling of homologous recombination DNA repair. Nat. Commun. *5*, 3361.

Poli, V., and Camporeale, A. (2015). STAT3-mediated metabolic reprograming in cellular transformation and implications for drug resistance. Front. Oncol. *5*, 121.

Posern, G., and Treisman, R. (2006). Actin' together: serum response factor, its cofactors and the link to signal transduction. Trends Cell Biol. *16*, 588–596.

Qian, B.Z., Li, J., Zhang, H., Kitamura, T., Zhang, J., Campion, L.R., Kaiser, E.A., Snyder, L.A., and Pollard, J.W. (2011). CCL2 recruits inflammatory monocytes to facilitate breast-tumour metastasis. Nature *475*, 222–225.

Riaz, N., Havel, J.J., Makarov, V., Desrichard, A., Urba, W.J., Sims, J.S., Hodi, F.S., Martin-Algarra, S., Mandal, R., Sharfman, W.H., et al. (2017). Tumor and microenvironment evolution during immunotherapy with nivolumab. Cell *171*, 934–949.e16.

Rizos, H., Menzies, A.M., Pupo, G.M., Carlino, M.S., Fung, C., Hyman, J., Haydu, L.E., Mijatov, B., Becker, T.M., Boyd, S.C., et al. (2014). BRAF inhibitor resistance mechanisms in metastatic melanoma: spectrum and clinical impact. Clin. Cancer Res. *20*, 1965–1977.

Robert, C., Karaszewska, B., Schachter, J., Rutkowski, P., Mackiewicz, A., Stroiakovski, D., Lichinitser, M., Dummer, R., Grange, F., Mortier, L., et al. (2015). Improved overall survival in melanoma with combined dabrafenib and trametinib. N. Engl. J. Med. *372*, 30–39.

Roca, H., Varsos, Z.S., Sud, S., Craig, M.J., Ying, C., and Pienta, K.J. (2009). CCL2 and interleukin-6 promote survival of human CD11b+ peripheral blood mononuclear cells and induce M2-type macrophage polarization. J. Biol. Chem. *284*, 34342–34354.

Sadok, A., McCarthy, A., Caldwell, J., Collins, I., Garrett, M.D., Yeo, M., Hooper, S., Sahai, E., Kuemper, S., Mardakheh, F.K., and Marshall, C.J. (2015). Rho kinase inhibitors block melanoma cell migration and inhibit metastasis. Cancer Res. *75*, 2272–2284.

Sahai, E., and Marshall, C.J. (2002). RHO-GTPases and cancer. Nat. Rev. Cancer *2*, 133–142.

Samuel, M.S., Lopez, J.I., McGhee, E.J., Croft, D.R., Strachan, D., Timpson, P., Munro, J., Schroder, E., Zhou, J., Brunton, V.G., et al. (2011). Actomyosin-mediated cellular tension drives increased tissue stiffness and beta-catenin activation to induce epidermal hyperplasia and tumor growth. Cancer Cell *19*, 776–791.

Sanz-Moreno, V., Gadea, G., Ahn, J., Paterson, H., Marra, P., Pinner, S., Sahai, E., and Marshall, C.J. (2008). Rac activation and inactivation control plasticity of tumor cell movement. Cell *135*, 510–523.

Sanz-Moreno, V., Gaggioli, C., Yeo, M., Albrengues, J., Wallberg, F., Viros, A., Hooper, S., Mitter, R., Feral, C.C., Cook, M., et al. (2011). ROCK and JAK1 signaling cooperate to control actomyosin contractility in tumor cells and stroma. Cancer Cell *20*, 229–245.

Shaltiel, I.A., Krenning, L., Bruinsma, W., and Medema, R.H. (2015). The same, only different—DNA damage checkpoints and their reversal throughout the cell cycle. J. Cell Sci. *128*, 607–620.

Sharma, P., Hu-Lieskovan, S., Wargo, J.A., and Ribas, A. (2017). Primary, adaptive, and acquired resistance to cancer immunotherapy. Cell *168*, 707–723.

Smith, M.P., Sanchez-Laorden, B., O'Brien, K., Brunton, H., Ferguson, J., Young, H., Dhomen, N., Flaherty, K.T., Frederick, D.T., Cooper, Z.A., et al. (2014). The immune microenvironment confers resistance to MAPK pathway inhibitors through macrophage-derived TNFalpha. Cancer Discov. *4*, 1214–1229.

Song, C., Piva, M., Sun, L., Hong, A., Moriceau, G., Kong, X., Zhang, H., Lomeli, S., Qian, J., Yu, C.C., et al. (2017). Recurrent tumor cell-intrinsic and -extrinsic alterations during MAPKi-induced melanoma regression and early adaptation. Cancer Discov. *7*, 1248–1265.

Sun, C., Wang, L., Huang, S., Heynen, G.J., Prahallad, A., Robert, C., Haanen, J., Blank, C., Wesseling, J., Willems, S.M., et al. (2014). Reversible and adaptive resistance to BRAF(V600E) inhibition in melanoma. Nature *508*, 118–122.

Takaki, T., Montagner, M., Serres, M.P., Le Berre, M., Russell, M., Collinson, L., Szuhai, K., Howell, M., Boulton, S.J., Sahai, E., and Petronczki, M. (2017). Actomyosin drives cancer cell nuclear dysmorphia and threatens genome stability. Nat. Commun. *8*, 16013.

Tauriello, D.V.F., Palomo-Ponce, S., Stork, D., Berenguer-Llergo, A., Badia-Ramentol, J., Iglesias, M., Sevillano, M., Ibiza, S., Canellas, A., Hernando-Momblona, X., et al. (2018). TGFbeta drives immune evasion in genetically reconstituted colon cancer metastasis. Nature *554*, 538–543.

Das Thakur, M., Salangsang, F., Landman, A.S., Sellers, W.R., Pryer, N.K., Levesque, M.P., Dummer, R., McMahon, M., and Stuart, D.D. (2013). Modelling vemurafenib resistance in melanoma reveals a strategy to forestall drug resistance. Nature *494*, 251–255.

Titz, B., Lomova, A., Le, A., Hugo, W., Kong, X., Ten Hoeve, J., Friedman, M., Shi, H., Moriceau, G., Song, C., et al. (2016). JUN dependency in distinct early and late BRAF inhibition adaptation states of melanoma. Cell Discov. *2*, 16028.

Di Veroli, G.Y., Fornari, C., Wang, D., Mollard, S., Bramhall, J.L., Richards, F.M., and Jodrell, D.I. (2016). Combenefit: an interactive platform for the analysis and visualization of drug combinations. Bioinformatics *32*, 2866–2868.

Vicente-Manzanares, M., Ma, X., Adelstein, R.S., and Horwitz, A.R. (2009). Non-muscle myosin II takes centre stage in cell adhesion and migration. Nat. Rev. Mol. Cell Biol. *10*, 778–790.

Wagle, N., Emery, C., Berger, M.F., Davis, M.J., Sawyer, A., Pochanard, P., Kehoe, S.M., Johannessen, C.M., Macconaill, L.E., Hahn, W.C., et al. (2011). Dissecting therapeutic resistance to RAF inhibition in melanoma by tumor genomic profiling. J. Clin. Oncol. *29*, 3085–3096.

Wagle, N., Van Allen, E.M., Treacy, D.J., Frederick, D.T., Cooper, Z.A., Taylor-Weiner, A., Rosenberg, M., Goetz, E.M., Sullivan, R.J., Farlow, D.N., et al. (2014). MAP kinase pathway alterations in BRAF-mutant melanoma patients with acquired resistance to combined RAF/MEK inhibition. Cancer Discov. *4*, 61–68.

Wang, L., Leite de Oliveira, R., Huijberts, S., Bosdriesz, E., Pencheva, N., Brunen, D., Bosma, A., Song, J.Y., Zevenhoven, J., Los-de Vries, G.T., et al. (2018). An acquired vulnerability of drug-resistant melanoma with therapeutic potential. Cell *173*, 1413–1425.e14.

Wolf, K., Muller, R., Borgmann, S., Brocker, E.B., and Friedl, P. (2003). Amoeboid shape change and contact guidance: T-lymphocyte crawling through fibrillar collagen is independent of matrix remodeling by MMPs and other proteases. Blood *102*, 3262–3269.

Zhang, W. (2015). BRAF inhibitors: the current and the future. Curr. Opin. Pharmacol. *23*, 68–73.

STAR★METHODS

KEY RESOURCES TABLE

REAGENT or RESOURCE	SOURCE	IDENTIFIER
Antibodies		
pT202/Y204-p44/42 (ERK1/2)	Cell Signaling Technology	Cat# 4370; RRID:AB_2315112
pThr18/Ser19-MLC2	Cell Signaling Technology	Cat# 3674; RRID: AB_2147464
pSer19-MLC2	Cell Signaling Technology	Cat# 3671; RRID: AB_330248
MLC2	Cell Signaling Technology	Cat# 3672; RRID: AB_10692513
PD-L1 clone E1L3N	Cell Signaling Technology	Cat# 13684; RRID:AB_2687655
pY705-STAT3	Cell Signaling Technology	Cat# 9145; RRID:AB_2491009
ERK2	Santa Cruz Biotechnology	Cat# sc-154; RRID:AB_2141292
GAPDH	Santa Cruz Biotechnology	Cat# MAB374; RRID:AB_2107445
GFP	Santa Cruz Biotechnology	Cat# sc-8334; RRID:AB_641123
MCL-1	Santa Cruz Biotechnology	Cat# sc-819; RRID:AB_2144105
STAT3	Santa Cruz Biotechnology	Cat# sc-482; RRID:AB_632440
Rat IgG2a anti-PD-1 clone RMP1-14	BioXCell	Cat# BE0146; RRID:AB_10949053
Rat IgG2a isotype control clone 2A3	BioXCell	Cat# BE0089; RRID:AB_1107769
CD206	Abcam	Cat# ab64693; RRID:AB_1523910
CD3 anti-mouse	Abcam	Cat# ab134096
CD4 anti-mouse, clone I3T4	Abcam	Cat# ab183685; RRID:AB_2686917
FoxP3 anti-human, clone 236A/E7	Abcam	Cat# ab20034; RRID:AB_445284
P-H2A.X (S139)	Abcam	Cat# ab2893; RRID:AB_303388
CD8a anti-mouse, clone Ly2	Invitrogen	Cat# 14-0808-82; RRID:AB_2572861
F4/80 anti-mouse, clone BM8	Invitrogen	Cat# MF48000; RRID:AB_10376289
FoxP3 anti-mouse, clone FJK-16s	Invitrogen	Cat# 14-5773-82; RRID:AB_467576
CD4 anti-human, clone 11E9	Novocastra	Cat# NCL-L-CD4-368; RRID:AB_563559
Biological Samples		
Human melanoma pre-/post-therapy	Paul Lorigan, Richard Marais	N/A
Chemicals, Peptides, and Recombinant Proteins		
PLX4720	Selleck	#S1152
PLX4032	Selleck	#S1267
GSK2118436 Dabrafenib	ChemieTek	#CT-DABRF
GSK1120212 Trametinib	Selleck	#S2673
PD184352	Selleck	#S1020
AZD6244	Selleck	#S1008
SCH772984	Selleck	#S7101
GSK269962A	Axon MedChem	# Axon 1167
H1152	Calbiochem	#555550
AT13148	Selleck	#S7563
(±)-Blebbistatin	Calbiochem	#203390
Critical Commercial Assays		
Human/Mouse TGF-β1 ELISA	Biolegend	#436707
Trichrome Stain (Masson) Kit	Sigma	#HT15-1KT
Bouin's solution	Sigma	#HT10132
Weigert's iron hematoxylin solution	Sigma	# HT1079

(*Continued on next page*)

Continued

REAGENT or RESOURCE	SOURCE	IDENTIFIER
Deposited Data		
Mass spectrometry A375 MEKi 24h	This study	ProteomeXchange via PRIDE repository Project # PXD002621 (https://www.ebi.ac.uk/pride/archive/projects/PXD002621)
Experimental Models: Cell Lines		
Human: A375	ATCC	ATCC Cat# CRL-7904; RRID:CVCL_0132
Human: Colo829	ATCC	ATCC Cat# CRL-1974; RRID:CVCL_1137
Human: SKMEL5	ATCC	ATCC Cat# HTB-70; RRID:CVCL_0527
Human: WM88	Coriell Institute	Coriell Cat# WC00123; RRID:CVCL_6805
Human: WM983A	Coriell Institute	Coriell Cat# WC00048; RRID:CVCL_6808
Human: WM983B	Coriell Institute	Coriell Cat# WC00066; RRID:CVCL_6809
Human: WM793B	Coriell Institute	Coriell Cat# WC00062; RRID:CVCL_8787
Human: A375M2	Richard Hynes	Clark et al., 2000
Human: LOX-IMVI	Øystein Fodstad	RRID:CVCL_1381
Human: D04	Kevin Harrington	RRID:CVCL_H604
Human: MM485	Wellcome Trust Functional Genomics Cell Bank	RRID:CVCL_2610
Human: A375/PLX/R	Richard Marais	RRID:CVCL_IW10<br>Baenke et al., 2015
Human: Colo829/PLX/R	Richard Marais	RRID:CVCL_IW11<br>Baenke et al., 2015
Human: A375/D+T/R	Richard Marais	N/A
Human: Patient #2	Richard Marais	N/A
Human: Patient #35	Richard Marais	N/A
Human: Patient #62T3	Richard Marais	N/A
Human: Patient #58	Richard Marais	N/A
Human: Patient #33	Richard Marais	N/A
Mouse: 5555	Richard Marais	Dhomen et al., 2009; Hirata et al., 2015
Mouse: 5555-anti-PD-1/NR	This paper	N/A
Mouse: 4434	Richard Marais	Dhomen et al., 2009; Hirata et al., 2015
Mouse: 4599	Richard Marais	Dhomen et al., 2009; Hirata et al., 2015
Mouse: 690cl2	Richard Marais	Dhomen et al., 2009
Human: HEK293T	Jeremy Carlton	ATCC Cat# CRL-3216; RRID:CVCL_0063
Experimental Models: Organisms/Strains		
Mouse: CD-1 nu/nu	Charles River UK	RRID:IMSR_CRL:086
Mouse: NOD/SCID/IL-2Rγ-/- (NSG)	Charles River UK	RRID:IMSR_JAX:005557
Mouse: C57BL/6J	Charles River UK	RRID:IMSR_JAX:000664
Oligonucleotides		
See Table S7 for RNAi sequences	Dharmacon	
Recombinant DNA		
pEGFP-N3-EGFP	Fernando Calvo	Takara, Clontech #U57609
pEGFP-N3-MLC2-EGFP (rat MLC2)	Fernando Calvo	Calvo et al., 2013
pEGFP-N3-MLC2-TASA (T18A S19A) -EGFP	Fernando Calvo	Calvo et al., 2013
pEGFP-N3-MLC2-TDSD (T18D S19D) -EGFP	Fernando Calvo	Calvo et al., 2013
pLVX-EGFP	Erik Sahai, Tohru Takaki	Takara, Clontech #632164
pLVX-MLC2-EGFP	Erik Sahai, Tohru Takaki	Takaki et al., 2017

(*Continued on next page*)

Continued		
REAGENT or RESOURCE	SOURCE	IDENTIFIER
pLVX-MLC2-TASA (T18A S19A)-EGFP	Erik Sahai, Tohru Takaki	Takaki et al., 2017
pLVX-MLC2-TDSD (T18A S19A)-EGFP	Erik Sahai, Tohru Takaki	Takaki et al., 2017
Software and Algorithms		
GSEA, ssGSEA	Broad Institute http://www.broadinstitute.org/gsea/index.jsp	N/A
ImageJ	https://imagej.nih.gov/ij/	N/A
GraphPad Prism 8	GraphPad Software	N/A
SPSS	IBM	N/A

LEAD CONTACT AND MATERIALS AVAILABILITY

Further information and reasonable requests for resources and reagents should be directed to and will be fulfilled by the Lead Contact, Victoria Sanz-Moreno (v.sanz-moreno@qmul.ac.uk). All unique/stable reagents generated in this study are available from the Lead Contact with a completed Materials Transfer Agreement.

EXPERIMENTAL MODEL AND SUBJECT DETAILS

Patient-Derived Samples

Human melanoma samples were a kind gift from Paul Lorigan (University of Manchester). Tumor samples were collected under the Manchester Cancer Research Centre (MCRC) Biobank ethics application #07/H1003/161+5 with full informed consent from the patients. The work presented in this manuscript was approved by MCRC Biobank Access Committee application 13_RIMA_01. Patient sample information is in Table S6.

Cell Lines and Patient-Derived Cell Lines

Cell lines used are listed in the Key Resources Table. Cell lines were cultured under standard conditions in complete medium (DMEM or RPMI medium supplemented with 10% fetal bovine serum (FBS) and 1% penicillin/streptomycin (all from Gibco)). Cell lines were tested to be free from mycoplasma contamination. All melanoma cell lines used were $BRAF^{V600E}$ unless otherwise stated. A375, Colo829 and SKMEL5 cells were from ATCC. WM88, WM983A, WM983B, WM793B were purchased from Coriell Institute. A375M2 were from Dr Richard Hynes (HHMI, MIT, USA). LOX-IMVI cell line was a gift from Prof Øystein Fodstad (Oslo University Hospital). SKMEL5, WM983A, WM983B, WM793B, LOX-IMVI were grown in complete RPMI, WM88 was grown in complete DMEM. PLX4720-resistant WM983A, WM983B and WM88 cells were derived after exposure to PLX4720 for 2-3 months (1 μM PLX4720 for WM983A and WM983B; 0.5 μM PLX4720 for WM88), controls were treated with equivalent volume of DMSO.

PLX4720-resistant (A375/PLX/R, Colo829/PLX/R) (Baenke et al., 2015) and dabrafenib+trametinib-resistant (A375/D+T/R) cell lines were a kind gift from Richard Marais (Cancer Research UK Manchester Institute). Resistant cells were generated after exposure of parental A375 and Colo829 to increasing concentrations of drugs (up to 1 μM PLX4720; 1 μM dabrafenib plus 10 nM trametinib) until cells resumed growth. Cells were grown in complete DMEM (A375-derivatives) or complete RPMI (Colo829-derivatives) supplemented with 1 μM PLX4720 (A375/PLX/R and Colo829/PLX/R cells); 1 μM dabrafenib plus 10 nM trametinib (A375/D+T/R) or equivalent volume of DMSO (parental A375 and Colo829 cells).

Patient-derived melanoma cell lines (#2, #35, #62T3, #58, #33) were a very kind gift from Richard Marais and were grown in RPMI. Patient #2, #35, #62T3 were grown in complete RPMI supplemented with 1 μM PLX4720. Patient #2 cell line was established from a patient with stage IV BRAF mutant melanoma with primary resistance to vemurafenib and ipilimumab. Patient #/35 cell line was established from a lymph node metastasis after treatment with vemurafenib for 3 months. Patient #62T3 cell line was established from a resected tumor upon disease progression following vemurafenib treatment (acquired resistance) and immunotherapy (refractory to ipilimumab and subsequent pembrolizumab). Patient #58 cell line (wild-type for BRAF/NRAS) was established from a metastasis from a patient that never responded to ipilimumab treatment (3 months). Patient #33 cell line ($BRAF^{K601E}$) was established from a metastasis from a patient that never responded to ipilimumab treatment (1 month). Patient #58 and #33 had also been treated with dacarbazine (DTIC) before ipilimumab. Patient #26 cell lines were established before and after nivolumab treatment.

$Braf^{V600E}$ mouse melanoma cell lines 5555, 4434, 4599 and 690cl2 (from Richard Marais) were established from the following C57BL/6 mouse models: $Braf^{+/LSL-V600E}$;Tyr::$CreERT2^{+/o}$;$p16^{INK4a-/-}$ (5555, 4434); $Braf^{+/LSL-V600E}$;Tyr::$CreERT2^{+/o}$ (4599); Pten-null $Braf^{+/LSL-V600E}$;Tyr::$CreERT2^{+/o}$;$p16^{INK4a-/-}$;Pten-/- (690cl2) (Dhomen et al., 2009; Hirata et al., 2015). NRAS mutant cell lines used: D04 was from Kevin Harrington (The Institute of Cancer Research); MM485 was obtained from the Wellcome Trust Functional Genomics Cell Bank (UK). HEK293T cells were from Jeremy Carlton (The Francis Crick Institute).

A375, A375/PLX/R, Colo829, Colo829/PLX/R, SKMEL5 cells and Patient-derived cell lines were confirmed by STR profiling at CRUK Manchester Institute; A375M2, WM983A, WM983B at King's College London; WM88 and WM793B cells were purchased from Coriell Institute in June 2014.

Animals

All animals were maintained under specific pathogen-free conditions and handled in accordance with the Institutional Committees on Animal Welfare of the UK Home Office (The Home Office Animals Scientific Procedures Act, 1986). All animal experiments were approved by the Ethical Review Process Committees at Barts Cancer Institute, King's College London and The Francis Crick Institute, in accordance with the Animals (Scientific Procedures) Act 1986 and according to the guidelines of the Committee of the National Cancer Research Institute.

Animals used in this study were from Charles River UK: 5-week-old female nude CD-1 nu/nu mice; 5-8-week old NOD/SCID/IL-2Rγ-/- (NSG) mice (male and female); 5-7-week-old female C57BL/6J mice. Tumors were allowed to establish, sizes (average 60-100 mm^3) were matched and then mice were randomly allocated to groups of 6-8 animals. No blinding was used in the treatment schedules for these experiments since the different treatments were identified by ear notching/mark on tail. Based on previous studies in the literature (Hong et al., 2017; Kong et al., 2017) and our own experience, groups of 6-8 animals were used to have sufficient animals per group to provide statistically significant data while keeping the number of animals used to a minimum. Tumor size was determined by caliper measurements of tumor length, width and depth and tumor volume was calculated as volume = 0.5236 x length x width x depth (mm). Note that this formula calculates smaller tumors (approximately 2-fold smaller) compared to those calculated using the formula volume = 0.5236 x length x $width^2$ (mm).

METHOD DETAILS

Chemicals

Chemicals used in this study (stocks resuspended in DMSO unless otherwise stated): BRAFi PLX4720 and PLX4032 (Selleck), BRAFi Dabrafenib (GSK2118436, ChemieTek), MEKi Trametinib (GSK1120212, Selleck), MEKi PD184352 (Selleck), MEKi AZD6244 (Selleck), ERKi SCH772984 (Selleck), ROCKi GSK269962A (Axon Medchem), ROCKi H1152 (resuspended in water; Calbiochem), AGC kinase inhibitor and ROCKi AT13148 (Selleck), myosin II inhibitor blebbistatin (in 95% DMSO; Calbiochem). Concentrations used unless otherwise stated in other STAR Methods sections: 5 μM ROCKi GSK269962A, 5 μM ROCKi H1152, 5 μM ROCKi AT13148, 25 μM myosin II inhibitor blebbistatin, 5 μM BRAFi PLX4720. "Analysis of cell morphology" section lists the inhibitors and concentrations used for those experiments.

Antibodies

Antibodies and concentrations used: pThr18/Ser19-MLC2 (#3674; 1:750, immunoblot), pSer19-MLC2 (#3671; 1:50, immunohistochemistry; 1:200, immunofluorescence), MLC2 (#3672; 1:750), pT202/Y204-p44/42 (ERK1/2) (#4370; 1:1,000), pY705-STAT3 (#9145; 1:750), PD-L1 (clone E1L3N, #13684, 1:200) from Cell Signaling Technology; STAT3 (sc-482; 1:500), ERK2 (sc-154; 1:1,000), MCL-1 (sc-819; 1:1,000), GFP (sc-8334; 1:1,000) from Santa Cruz Biotechnology; GAPDH (MAB374; 1:10,000) from Millipore; P-H2A.X (S139) (ab2893;1:1000), CD206 (ab64693; 1:1,000), CD3 (anti-mouse, ab134096; 1:500), CD4 (anti-mouse, clone I3T4, ab183685; 1:300), FoxP3 (anti-human, clone 236A/E7, ab20034; 1:200) from Abcam; F4/80 (anti-mouse, clone BM8, MF48000, 1:1000), CD8a (anti-mouse, clone Ly2, 14-0808-82; 1:200), FoxP3 (anti-mouse, clone FJK-16s, 14-5773-82; 1:200) from Invitrogen; CD4 (anti-human, clone 11E9, NCL-L-CD4-368; 1:300) from Novocastra.

Analysis of Cell Morphology

Cell morphology was analyzed on still phase-contrast images (cells on plastic or on collagen I) using ImageJ software (http://rsb.info.nih.gov/ij/). In order to quantify cell morphology on 2D and on collagen matrices, the morphology descriptor Circularity was used after manually drawing around the cell. Values closer to 1 represent rounded morphology; values closer to 0 represent more spread and/or spindle-shaped cells with multiple protrusions.

Treatments were for 24 hr as follows: A375M2 cells with 50 nM BRAFi PLX4720, 0.1 nM MEKi GSK1120212, 1 μM ROCKi GSK269962A (Figure 1B); WM983A/B cells with 5 μM ROCKi GSK269962A, 5 μM BRAFi PLX4720 (Figures 1D and 1E); 690cl2 cells with 200 nM MEKi PD184352, 200 nM BRAFi PLX4032, 500 nM ERKi SCH772984 (Figures 1F and S1C); D04, MM485 cells with 50 nM MEKi GSK1120212, 50 nM AZD6244 (Figures 1F, S1D, and S1E); 4599 cells with 500 nM MEKi GSK1120212, 1 nM MEKi AZD6244 (Figure S1B). A375 and A375/PLX/R on plastic (Figure 3D); and Patient #2 cells on collagen I (Figure 5I) were treated with 5 μM ROCKi GSK269962A, 5 μM BRAFi PLX4720 or both.

Long-Term Survival

Long-term survival was performed on tissue culture plastic dishes unless otherwise specified. Cells were seeded in 6-well plates (10,000 cells/well) and treated for 5-14 days, re-adding drugs in fresh media every 2-3 days (daily for blebbistatin). Then cells were fixed with 1% formaldehyde and stained with 0.25% crystal violet. Plates were scanned and images analyzed using ImageJ software. For experiments with inhibitors, percentage of the well covered by crystal violet-stained cells was calculated and shown relative to control cells. For dose-response experiments, cells were seeded in 12-well or 96-well plates and survival was analyzed

after 3-5 days treatment with indicated drugs using crystal violet. Crystal violet was solubilized with 10% acetic acid and absorbance was measured at 590 nm. In dose-response experiments, BRAFi-resistant cells were cultured in the presence of BRAFi throughout the experiment unless otherwise stated. In Figure 5C, 4434- and 5555-derivatives were treated with 0.1 μM ROCKi.

For synergy experiments, 1,000 A375 cells were seeded in 96-well plates, cultured overnight and next day treated in quintuplicates with ROCKi GSK269962A or BRAFi PLX4720, either alone or in several combinations in complete medium. Three days later, plates were fixed, stained with crystal violet and solubilized and quantified as above. Values were normalized to vehicle controls and analyzed with Combenefit software (Loewe model) (Di Veroli et al., 2016). Average of 4 independent experiments is shown.

Long Term Survival on Collagen I Matrices

Cells were grown on collagen I matrices as described (Orgaz et al., 2014b). Briefly, bovine collagen I (PureCol, #5005-B; Advanced BioMatrix) thick gels were polymerized at 1.7 mg/ml in 24-well plates. Cells were seeded at 10,000 cell/well and treatments started 16 hr later for 5-14 days. In experiments using A375-derivatives, cells were treated with 1 μM ROCKi, 1 μM BRAFi or both. Patient-derived cell lines were treated with 5 μM ROCKi. Fresh complete media with drugs was added every 2-3 days. At the end of the experiment collagen I gels were fixed with 4% formaldehyde and phase-contrast images were taken. Percentage of area covered by cells was quantified using QuPath software Version 0.1.2 and a SLIC superpixel image segmentation (Gaussian sigma value 5 pixels, superpixel spacing 20 pixels) (Bankhead et al., 2017). Software was trained to identify cells and background (surrounding collagen). Detection measurements were then exported to Excel and values for area/pixel^2 were normalized to each untreated control as percentage of area covered by cells. For Patient #2 cells, spheroid-forming ability was quantified as the sum of areas occupied by spheroids from phase-contrast images using ImageJ.

MTT Assay

Cells were seeded in 96-well plates (2,000 cells/well). Drugs were added every 2 days. Three days after seeding, plates were incubated with MTT (3-(4,5-dimethylthiazol-2-yl)-2,5-diphenyl tetrazolium bromide; Millipore) following the manufacturer's instructions and absorbance measured at 572 nm. Background at 630 nm was subtracted and data represented as relative viability.

Cell Cycle Analysis

For DNA cell cycle analysis, floating and adherent cells were fixed in 70% ethanol at -20°C, washed in phosphate-buffered saline and treated with 40 μg/ml propidium iodide (PI) (Biolegend) and 100 μg/ml ribonuclease (Sigma) for 25 min at 37°C. Staining was detected using a FACS BD Canto II (BD Biosciences) and analyzed and plotted using FlowJo (FlowJo LLC). The starting gating of the whole cell population, excluding any debris, was performed with FSC-A/SSC-A. Using this as a parental gate, doublets were excluded using PerCP-Cy5.5-A/ PerCP-Cy5.5-W (PI). The gated singlets were represented as histograms for PerCP-Cy5.5-A to show the peaks for the cell cycle phases.

AnnexinV/Propidium Iodide FACS

Floating and adherent cells were collected, spun down, and labelled with FITC Annexin V Apoptosis Detection Kit with PI (#640914, Biolegend UK Ltd), following the manufacturer's instructions. Staining was detected using a FACS BD Canto II and analyzed and plotted using FlowJo. The starting gating of the whole cell population, excluding any debris, was performed with FSC-A/SSC-A. This was followed by a double exclusion of doublets using first FSC-H/FSC-W and then SSC-H/SSC-W. The gated singlets were then gated as 'quad gates' using FITC-A (AnnexinV) versus PerCP-Cy5.5-A (PI) and represented as FACS dot plots. Graphs show percentage of dead cells as the sum of percentage of early apoptotic (annexin V^{high}, propidium iodide^{low}) and percentage of late apoptotic/necrotic cells (annexin V^{high}, propidium iodide^{high}).

ROS Detection

Cells were treated with 1 μM ROCKi for 24 hr (A375 pair) or 48 hr (WM983A pair). Then cells were collected and ROS levels were detected using CellROX Green Flow Cytometry Assay Kit (C10492, Life Technologies), according to the manufacturer's instructions. FACS and gating strategy were as described in Cell Cycle section.

Time Lapse Microscopy

Multi-site bright-field microscopy of cells in 24-well plates was performed in a humidified chamber at 37°C and 5% CO_2 using a 10X/0.3 NA Plan Fluor ELWD objective lens on a fully motorized (Prior Scientific) multi-field Nikon TE2000 microscope with an ORCA camera (Hamamatsu) controlled by Micro-Manager (https://micro-manager.org/) and ImageJ. Sixteen hr after seeding, cells were treated for 72 hr with ROCKi, BRAFi or both in the presence of 1.5 μM PI to identify dead cells. Total number of cells, percentage of multinucleated (alive, dead) and total dead cells were quantified for 72 hr.

RNAi

One hundred thousand cells were plated per 35-mm dish and transfected the next day with 26 nM siRNA oligonucleotides, using Optimem-I and Lipofectamine 2000 (Invitrogen). Forty eight hr after transfection cells were harvested and equal numbers re-seeded on 35-mm wells. Cells were transfected again 2 days later and plates were fixed and stained with crystal violet 2-4 days after the second transfection. Crystal violet was solubilized and absorbance at 590 nm measured as above. Cells were grown in the presence

of 1 μM PLX4720 during the whole experiment. All siRNA sequences were On-Targetplus (OT) from Dharmacon (Lafayette, USA) and are listed in Table S7.

MLC2 Rescue Experiments

One hundred thousand cells were plated per 35-mm dish and transfected the next day with Lipofectamine 2000 and 1 μg plasmid encoding GFP (as control), wild-type rat MLC2 (*MYL12B*) fused with GFP or inactive phospho-mutant TASA-MLC2 fused with GFP (T18A, S19A) (Calvo et al., 2013) (plasmids were a gift from Fernando Calvo). Next day cells were transfected with 26 nM siRNA oligonucleotides against *MYL12B*. Cell death was assessed 2-3 days after siRNA transfection by PI (1.5 μM) incorporation by FACS. Percentage of dead cells (PI^+) was quantified within transfected (GFP^+) cells.

MLC2 Stable Overexpression

Lentivectors encoding EGFP-fused rat MLC2-derivatives (wild-type, phospho-mimetic TDSD (T18D, S19D) and inactive phospho-mutant TASA (T18A, S19A)) (Takaki et al., 2017) were a kind gift from Erik Sahai and Tohru Takaki (The Francis Crick Institute). HEK293T cells were transfected with MLC2-lentivectors and packaging plasmids using standard procedures, and after 48 hr supernatants were collected and spun down to remove debris. A375 cells were transduced with lentiviral supernatants for 8 hr, and 48 hr later cells were selected with 1 μg/ml puromycin for 5 days, then cells were used for subsequent experiments.

Immunofluorescence and Confocal Imaging

Cells were fixed with 4% formaldehyde, permeabilised with 0.2% Triton X-100 for 5 min, blocked with 5% BSA-PBS for 1 hr at room temperature, and incubated with anti-p-MLC2 (p-MLC2S19, 1:200 in 5% BSA-PBS) overnight at 4°C. Alexa-488 anti-rabbit secondary antibody (Life Technologies) was used at 1:500 for 1 hr at room temperature. F-actin was detected with Phalloidin (1 hr RT) and nuclei were stained with Hoechst 33258 (Life Technologies). Imaging was carried out on a Zeiss LSM 510 Meta confocal microscope (Carl Zeiss) with C-Apochromat × 40/1.2 NA (water) or a Plan Apochromat × 63/1.4 NA (oil) objective lenses and Zen software (Carl Zeiss). Line scan analysis was performed in ImageJ.

Immunoblotting

Cells were lysed in Laemmli buffer and snap frozen. Lysates were then boiled, sonicated for 15 s and spun down. Cell lysates were fractionated using sodium dodecyl sulfate-polyacrylamide (SDS-PAGE) gels in non-reducing conditions, and transferred subsequently to PVDF filters. Membranes were blocked in 5% BSA in 0.1% Tween 20-TBS. Primary antibodies were incubated overnight at 4°C. For detection, ECL or Prime ECL detection systems coupled to HRP-conjugated secondary antibodies (GE Healthcare) with X-ray films and an Amersham Imager 600 were used. Bands were quantified using ImageJ. Levels of phospho-proteins were calculated after correction to total levels of the relevant protein.

TGF-β1 ELISA

Cells were seeded on T6-well plates (150,000 cells/well), next day cells were washed 3 times and then grown in serum-free media with or without ROCKi GSK269962A (5 μM). Forty-eight hr later supernatants were collected, spun down and assayed fresh or frozen at -80°C. TGF-β1 levels were detected by ELISA using Total TGF-β1 Legend Max^{TM} ELISA Kit with Pre-coated plate (#436707, Biolegend) on neat samples diluted 1/5 following the manufacturer's instructions.

Phospho-proteomics

Preparation of tandem mass tagged (TMT)-multivariate phosphoproteomic samples. Cells treated with MEKi (200 nM GSK1120212 trametinib or 200 nM PD184352) or vehicle (DMSO) for 24 hr were lysed in 6 M urea, sonicated, centrifuged to clear cell debris and protein concentration was determined by BCA (Pierce 23225). 100 μg of each condition was individually digested by FASP (PMID: 19377485) using 1:100 Lys-C (Wako 125-05061), 1:100 Trypsin (Worthington), and amine-TMT-10 plex labeled (Pierce 90111) on membrane (iFASP) (PMID: 23692318). TMT channel assignment: 126 = Control (Bio. Rep. 1); 127N = Control (Bio. Rep. 2), 127C = Control (Bio. Rep. 3); 128N = Control (Bio. Rep. 4); 128C = MEKi A (Bio. Rep. 1); 129N = MEKi A (Bio. Rep. 2); 129C = MEKi A (Bio. Rep. 3); 130N = MEKi B (Bio. Rep. 1); 130C = MEKi B (Bio. Rep. 2); 131 = MEKi B (Bio. Rep. 3) (A= GSK1120212, B= PD184352). Peptides were then eluted, pooled, lyophilized and subjected to automated phosphopeptide enrichment (APE) (PMID: 25233145). Phosphopeptides were desalted using OLIGO R3 resin (Life Technologies 1-1339-03) and lyophilised prior to LC-MS/MS analysis (see below).

Data-dependent acquisition LC-MS/MS. Phosphopeptide samples were resuspended in 0.1% formic acid and analyzed on a Q-Exactive Plus mass spectrometer (Thermo Scientific) coupled to a Dionex Ultimate 3000 RSLCnano System (Thermo Scientific). Reversed-phase chromatographic separation was performed on a C18 PepMap 300 Å trap cartridge (0.3 mm i.d. x 5 mm, 5 μm bead size; loaded in a bi-directional manner), a 75 μm i.d. x 50 cm column (5 μm bead size) using a 120 min linear gradient of 0-50% solvent B (MeCN 100% + 0.1% formic acid (FA)) against solvent A (H2O 100% + 0.1% FA) with a flow rate of 300 nL/min. The mass spectrometer was operated in the data-dependent mode to automatically switch between dual Orbitrap MS and MS/MS acquisition. Survey full scan MS spectra (from m/z 400-2000) were acquired in the Orbitrap with a resolution of 70,000 at m/z 400 and FT target value of 1 x 106 ions. The 20 most abundant ions were selected for fragmentation using higher-energy collisional dissociation (HCD) and dynamically excluded for 30 s. Fragmented ions were scanned in the Orbitrap at a resolution 35,000 at m/z 400. The isolation window

was reduced to 1.2 m/z (to reduce ion co-isolation) and a MS/MS fixed first mass of 120 m/z was used (to ensure consistent TMT reporter ion coverage). For accurate mass measurement, the lock mass option was enabled using the polydimethylcyclosiloxane ion (m/z 445.120025) as an internal calibrant.

For peptide identification, raw data files produced in Xcalibur 2.1 (Thermo Scientific) were processed in Proteome Discoverer 1.4 (Thermo Scientific) and searched against Human Unitprot database using Mascot (v2.2). Searches were performed with a precursor mass tolerance set to 10 ppm, fragment mass tolerance set to 0.05 Da and a maximum number of missed cleavages set to 2. Static modifications were limited to carbamidomethylation of cysteine, and variable modifications used were oxidation of methionine, deamidation of asparagine/glutamine, and phosphorylation of serine, threonine and tyrosine residues. Peptides were further filtered using a mascot significance threshold <0.05, a peptide ion Score >20 and a FDR <0.01 (evaluated by Percolator (PMID: 17952086)). Phospho-site localization probabilities were calculated with phosphoRS 3.1 (>75%) (PMID: 22073976). For relative phosphopeptide quantification, MEKi/vehicle ratios were calculated by Proteome Discoverer 1.4. See Data and Code Availability section below for further details.

Phosphoproteomic data analysis. Phosphopeptides from Proteome Discoverer 1.4 were normalised against total protein levels (from SILAC in-gel digest experiments), and protein-level phospho-site locations (phosphoRS 3.1 score >75%, maximum 4-PTM/peptide) were manually annotated using PhosphoSitePlus. Precursor ion spectra, extracted ion chromatograms, and product ion spectra were manually inspected for each regulated phosphopeptide. Empirical parent kinases were manually identified by referenced Uniprot annotation and putative parent kinases were manually assigned using ScanSite (PMID: 12824383) 3 (top 1 percentile of all sites, lowest score). Phospho-sites that did not meet these conditions were not annotated. Regulated phospho-peptides in Table S1 were those which were significant across both MEKi (GSK1120212 and PD184352) compared to vehicle-treated cells.

Phospho-Peptide Enrichment Analysis

Pathway enrichment analyzes of the list of phospho-peptides increased in MEKi-treated A375 compared to vehicle-treated A375 cells (this study, see Phospho-proteomics section; Table S1); A375/PLX/R compared to A375 cells (data from (Girotti et al., 2013)) and M229- and M238-vemurafenib-resistant vs parental cells from (Titz et al., 2016) were performed using MetaCore from GeneGo Inc. (https://portal.genego.com/).

Quantitative Real Time One-Step PCR

RNA was isolated using TriZol (Life technologies). For experiments comparing expression in parental vs BRAFi-resistant cells (A375- and Colo829-derivatives), resistant cells were cultured with 1 μM PLX4720 and sensitive cells with equivalent volume of DMSO for 24 hr. QuantiTect Primer Assays (Qiagen) and Brilliant II SYBR Green QRT-PCR 1-step system (Agilent Technologies) with 100 ng RNA were used following the manufacturer's instructions. GAPDH was used as loading control. The following QuantiTect Primers were used (Qiagen): *GAPDH* (QT00079247), *LIMK1* (QT00008680), *LIMK2* (QT00084357), *MKL1* (QT00067921), *MKL2* (QT00010115), *MYH9* (QT00073101), *MYL9* (QT00072268), *MYL12A* (QT01665741), *MYL12B* (QT00075264), *ROCK1* (QT00034972), *ROCK2* (QT00011165). Primer sequences are not provided by Qiagen, as stated in their website: 'Sequences of the QuantiTect Primer Assays are not provided. Approximate location of primers within a specific gene can be viewed on the Product Detail pages retrieved via our GeneGlobe data base.'

Gene Expression Studies and Analysis

Normalized gene expression microarray and RNAseq (FPKM, fragments per kilobase of transcripts per million mapped reads) data from published studies were downloaded from Gene Expression Omnibus (GEO) unless otherwise stated: Hugo 2015 (GSE65185 and GSE65184) (Hugo et al., 2015); Hugo 2016 (GSE78220) (Hugo et al., 2016); Kakavand 2017 (GSE99898) (Kakavand et al., 2017); Kwong 2015 (European Genome-phenome Archive (EGA S00001000992)) (Kwong et al., 2015); Long 2014 (GSE61992) (Long et al., 2014a); Obenauf 2015 (GSE64741) (Obenauf et al., 2015); Rizos 2014 (GSE50509) (Rizos et al., 2014); Riaz 2017 (Ipi-naive cohort; GSE91061) (Riaz et al., 2017); Song 2017 (GSE75299, GSE103630) (Song et al., 2017); Sun 2014 (GSE50535) (Sun et al., 2014); Wagle 2014 (GSE77940) (Wagle et al., 2014). In patients with several biopsies, their average is shown (see Table S4).

RSEM-normalized expression data and clinical information of human melanoma samples (70 primary and 319 metastatic melanomas) from The Cancer Genome Atlas (TCGA) database were downloaded from Firehose (https://gdac.broadinstitute.org/). Only TCGA samples with no neo-adjuvant treatment prior to tumor resection were considered.

The ROCK-myosin II pathway expression signature (*MYL9, MYL12A, MYL12B, MYH9, ROCK1, ROCK2, LIMK1, LIMK2, MKL1, MKL2, MYLK, DAPK3*) was generated by the sum of normalized expression values of signature genes for each TCGA patient. ROCK-myosin II pathway signature was categorized as low or high using the mean expression.

Heatmaps and unsupervised hierarchical clustering analyzes were generated using Multiexperiment Viewer (http://www.tm4.org/mev.html). Distance metric used for the clustering was Euclidean distance. In patients with several biopsies, their average is shown.

Gene Enrichment Analyzes

Gene sets for cross-resistance processes (EMT, metastasis, angiogenesis, hypoxia, wound healing, TGF-β, STAT3, NF-κB, YAP) were downloaded and analyzed using Gene Set Enrichment Analysis (GSEA) software (http://www.broadinstitute.org/gsea/index.jsp) with the settings: permutations-1,000, permutation type-gene set, metric for ranking genes-t-test. Significantly enriched gene sets in resistant vs baseline samples were considered according to p value <0.05 and FDR <0.25 in at least 2 of the 5 comparisons

performed. To calculate the gene-signature score in each sample, we used single-sample Gene Set Enrichment Analysis (ssGSEA) Projection Software from GenePattern platform (https://www.broadinstitute.org/cancer/software/genepattern).

For the transcriptional signature of melanoma cells with high myosin II activity, genes upregulated in high myosin II activity compared to low myosin II activity melanoma cells (cells treated with ROCKi and blebbistatin) (Cantelli et al., 2015; Sanz-Moreno et al., 2011) were selected using a fold change $\geq$ 1.5 and a p value <0.01. GSEA analysis was performed as described above. Enrichment plot (green line) show upregulation of gene signature in indicated samples (resistant, non-responders or on-treatment). Nominal p values are shown along plot, false discovery rate (FDR) in figure legend.

For analysis of ROS-related gene signatures, all available ROS/oxidative stress gene sets were downloaded from GSEA Broad Institute (http://www.broadinstitute.org/gsea/index.jsp). Graph shows ($-Log_{10}$) p value.

For analysis of expression of DNA repair genes, we compiled a DNA repair gene signature from the list in (Mjelle et al., 2015) and the homologous recombination defect signature (Peng et al., 2014). Network enrichment analysis of genes commonly downregulated (<0.65-fold) in at least 4 of 7 cell lines from Group 1 (Figure 2B) was performed using Ingenuity Pathway Analysis (Qiagen).

Tumor Xenografts

A375/PLX/R cells (1 x 10^6) were injected subcutaneously into the right flank of 5-week-old female nude CD-1 mice (Charles River). Patient #2 cells (4 x 10^6) or Patient #35 cells (6 x 10^6) were injected into 5-8-week old NOD/SCID/ IL-2Rγ-/- (NSG, Charles River) mice (male and female). Tumors were allowed to establish, sizes (average 60-100 mm^3) were matched and then mice were randomly allocated to groups of 7-8 animals. Treatment was by orogastric gavage with 45 mg/kg PLX4720, 10-25 mg/kg GSK269962A or both drugs together. GSK269962A was used at 25 mg/kg for A375/PLX/R and 10 mg/kg for Patient #2, #35. Drugs were dissolved in 5% DMSO or in 6% DMSO+50% PEG300+ 9% Tween 80. All the drugs were administered daily, 7 days a week. Tumor size was determined by caliper measurements of tumor length, width and depth and tumor volume was calculated as volume = 0.5236 x length x width x depth (mm).

Immunotherapy Experiments

5555 cells (100,000, 250,000 or 1 million) were subcutaneously injected into the right flank of 5-7-week-old female C57BL/6J mice. After 7-14 days, mice with tumors (50-80 mm^3) were randomly allocated into groups of 6-7 animals and treated daily with ROCKi GSK269962A (10 mg/kg, oral gavage) or vehicle and every 3 days with anti-PD-1 monoclonal antibody (InVivoPlus clone RMP1-14, BioXCell #BE0146) (10 mg/kg, intraperitoneally (i.p.)) or rat IgG2a isotype control (clone 2A3 BioXCell # BE0089). Vehicle for ROCKi was 5% DMSO or 5% DMSO, 10% Tween 80, 6.5% ethanol. Tumor volume was determined as above. Anti-PD-1-non-responder (NR) lines were established in culture by digesting tumors with a mixture of Liberases (TH and TM, 75 μg/ml each, Roche Diagnostics) and 1 μg/ml DNase I (Sigma) in HBSS for 1 hr at 37°C with shaking, and then passed through 100 μM strainers. For experiments using 5555-anti-PD-1/NR cells, 1 million cells were injected subcutaneously into 7-week old C57BL/6J mice. Next day, all mice were given 1 dose of anti-PD-1 (10 mg/kg) i.p., and then again 3 days later. At day 7, mice were randomized into 4 treatment groups (ROCKi, anti-PD-1, combo or control) as above.

Survival in the Lung Assay

Patient #2 cells were pre-treated for 24 hr with 5 μM PLX4720, 5 μM GSK269962A or both (control had DMSO), then cells were labelled with 10 μM CMFDA-Green in OptiMem (Life Technologies) for 10 min, trypsinized and equal numbers were injected into the tail vein of NSG mice in 100 μl PBS along with drugs (same concentrations as pre-treatment). At the time of injection, mice (male and female) were 6-10 weeks old and weighed around 20-22 g; mice were age and sex-matched between the groups. Mice were sacrificed 30 min (to confirm that equal numbers arrived at the lung) and 24 hr after tail vein injection. The lungs were extracted, washed twice with PBS, fixed (4% formaldehyde for 16 hr at 4°C) and examined for fluorescently-labelled cells under a Zeiss LSM 510 Meta confocal microscope (Carl Zeiss) with a 20X objective. Lung retention is represented as fluorescence area (CMFDA-Green from melanoma cells) per field, and approximately 20 fields per mouse lung were analyzed. Each experiment had 4-5 mice/ condition, and experiments were replicated twice and data pooled together. Quantification of survival in the lung 24 hr after injection is shown as mean fluorescence area/field.

Immunohistochemistry

Tumors and spleens were formalin-fixed and paraffin-embedded using standard protocols. For cell pellets, transfected cells were harvested 48 hr after transfection using a cell scraper, spun down, fixed with 4% formalin for 30 min and washed with PBS. Cell pellet was resuspended in 2% agarose and then embedded in paraffin. Four μm thick sections were incubated at 60°C for 20 min and then subjected to antigen retrieval using Access Super Tris pH 9 buffer (A.Menarini Diagnostics) at 110°C for 6 min in a Decloaking Chamber NxGen (Biocare Medical). Samples were blocked with Dual Endogenous Enzyme-Blocking Reagent (Dako) for 10 min and then were incubated with primary antibodies for 40 min at RT, washed and then incubated with biotinylated secondary antibodies (rabbit, mouse or rat; 1:200; Vector-Labs) for 30 min at RT. Signal was then amplified using VECTASTAIN ABC HRP kit (PK-4000) for 20 min at RT and the reaction was developed using VIP substrate (SK-4600, Vector-Labs) for 10 min at RT. Stainings were counterstained with Hematoxylin. Positive and negative controls were included in each experiment, including staining of melanoma markers

HMB45/Melan-A or S100. For ECM staining, samples were fixed in Bouin's solution (HT10132, Sigma) for 1 hr at 60°C, then stained with Weigert's iron hematoxylin solution (HT1079, Sigma) for 5 min at RT and with Trichrome Stain (Masson) Kit (HT15-1KT, Sigma) following the manufacturer's instructions.

Imaging and Scoring

Sections from tumor xenograft experiments and from paired melanoma samples from 12 patients (tumor tissue before and after treatment) were imaged using NanoZoomer S210 slide scanner (Hamamatsu, Japan). Staining quantification was performed using QuPath 0.1.2 (Bankhead et al., 2017). For p-MLC2 stainings, whole sections were scanned and images were analyzed performing positive cell detection, and three different thresholds were applied according to the intensity scores (0, 1, 2 and 3). Next, the software was trained by creating random trees classification algorithm combined with the intensity information, in order to differentiate tumor from stroma, necrosis and immune cells. Values used in the analysis correspond to the quantification of p-MLC2 in the invasive front (mouse tumors) or highest score in the whole section (human samples).

To characterize the immune infiltrate (CD206, F4/80, CD3, CD4, CD8 and FOXP3) a similar approach was performed using QuPath. First, positive cell detection was applied, using only a single value to differentiate negative (blue) from positive (red). Data are represented as cellular density (cells/mm^2).

For PD-L1 analysis, $CD206^+$ cells were identified and both PD-L1 and CD206 stainings were aligned using QuPath 2.03m. From CD206 staining, positive detections ($CD206^+$) were transferred to PD-L1 in order to quantify the actual score for PD-L1 in $CD206^+$ cells. The negative detection for CD206 was used to quantify PD-L1 on tumor cells, these were identified as $CD206^-$ after discarding stromal/immune cells. Image composition was performed artificially attributing a color code, and images were overlaid using ImageJ (trackEM2). For PD-L1 and CD206, merge images in Figure 8I were generated with QuPath by overlaying pseudo-color images for each staining.

For ECM analysis with Masson's Trichrome staining, whole section images were quantified with QuPath applying a SLIC algorithm for segmentation of sections according to pixel density. Next, colors were deconvoluted and the green channel was used to quantify the percentage of the area occupied by collagen.

QUANTIFICATION AND STATISTICAL ANALYSIS

GraphPad Prism (GraphPad Software) was used to perform unpaired two-tailed t-test, Mann-Whitney test, Wilcoxon test, one-way or two-way ANOVA with post hoc tests (Tukey's, Dunnet's, Benjamini, Krieger and Yekutieli correction), Kruskal-Wallis, Deming linear regression, Spearman correlation and Chi-square test. Survival curves were estimated by the Kaplan-Meier method and the log-rank test using SPSS (IBM). Details of statistical analysis performed are in the figure legends. Bar graphs report mean ± SEM with individual data points as explained in figure legends. Box plots show median (center line); interquartile range (box); min-max values (whiskers). In Figure legends, "n" means number of independent experiments unless otherwise stated. Significance was defined as $p<0.05$. *$p<0.05$, **$p<0.01$, ***$p<0.001$, ****$p<0.0001$, ns not significant.

DATA AND CODE AVAILABILITY

The mass spectrometry proteomics data have been deposited to the ProteomeXchange Consortium (http://www.proteomexchange.org) via the PRIDE partner repository (PMID: 23203882) with the dataset identifier PXD002621 (https://www.ebi.ac.uk/pride/archive/projects/PXD002621).

Cancer Cell, Volume *37*

Supplemental Information

Myosin II Reactivation and Cytoskeletal Remodeling as a Hallmark and a Vulnerability in Melanoma Therapy Resistance

Jose L. Orgaz, Eva Crosas-Molist, Amine Sadok, Anna Perdrix-Rosell, Oscar Maiques, Irene Rodriguez-Hernandez, Jo Monger, Silvia Mele, Mirella Georgouli, Victoria Bridgeman, Panagiotis Karagiannis, Rebecca Lee, Pahini Pandya, Lena Boehme, Fredrik Wallberg, Chris Tape, Sophia N. Karagiannis, Ilaria Malanchi, and Victoria Sanz-Moreno

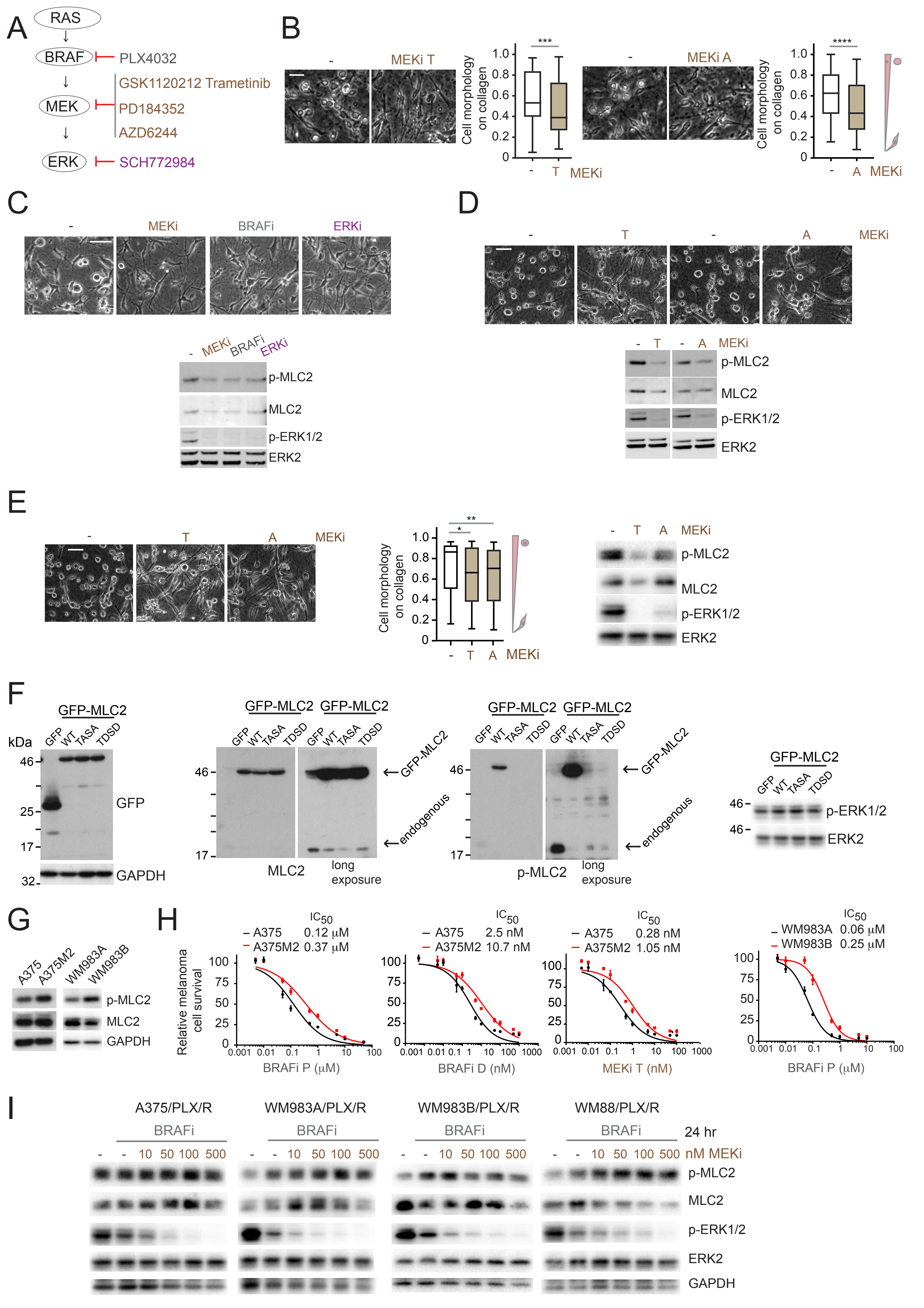
A
RAS
BRAF
PLX4032
MEK
GSK1120212 Trametinib
PD184352
AZD6244
ERK
SCH772984
B
-
MEKi T
Cell morphology on collagen
***
MEKi A
****
MEKi
C
MEKi
BRAFi
ERKi
p-MLC2
MLC2
p-ERK1/2
ERK2
D
T
A
E
*
**
F
GFP-MLC2
GFP
WT
TASA
TDSD
kDa
GFP
GAPDH
long exposure
endogenous
p-MLC2
G
A375
A375M2
WM983A
WM983B
H
IC50
A375 0.12 µM
A375M2 0.37 µM
A375 2.5 nM
A375M2 10.7 nM
A375 0.28 nM
A375M2 1.05 nM
WM983A 0.06 µM
WM983B 0.25 µM
Relative melanoma cell survival
BRAFi P (µM)
BRAFi D (nM)
MEKi T (nM)
I
A375/PLX/R
WM983A/PLX/R
WM983B/PLX/R
WM88/PLX/R
BRAFi
24 hr
10 50 100 500 nM MEKi

Figure S1, related to Figure 1. MAPK regulates Myosin II activity in melanoma. (**A**) Summary of inhibitors used. (**B**) Morphology of 4599 cells on collagen I after 24 hr treatment (MEKi Trametinib, AZD6244) (n>100 cells). (**C-D**) Images of (**C**) $BRAF^{V600E}$/Pten null 690cl2 cells and (**D**) $NRAS^{Q61L}$ D04 on collagen I after 24 hr treatments (690 cl2 MEKi PD184352, BRAFi PLX4032, ERKi SCH772984; D04, MEKi Trametinib, AZD6244). p-MLC2 and p-ERK1/2 immunoblots are shown. (**E**) Images and cell morphology quantification of $NRAS^{Q61R}$ MM485 cells on collagen I after 24 hr treatment (n=150 cells). (**F**) Immunoblots of A375 cells overexpressing WT or mutant MLC2. (**G**) p-MLC2 and MLC2 immunoblots. (**H**) Dose-response curves and IC_{50} values to indicated inhibitors of cells treated for 3 days (BRAFi PLX4720, BRAFi Dabrafenib, MEKi Trametinib) (n=3). (**I**) Immunoblots of BRAFi-resistant cell lines after 24 hr treatment (BRAFi PLX4720, MEKi Trametinib).

Scale bars (B-E), 50 µm. Box plots (B, E) show median (center line); interquartile range (box); min-max values (whiskers). p values by Kruskal Wallis with Dunn's correction. *p<0.05, **p<0.01, ***p<0.001, ****p<0.0001.

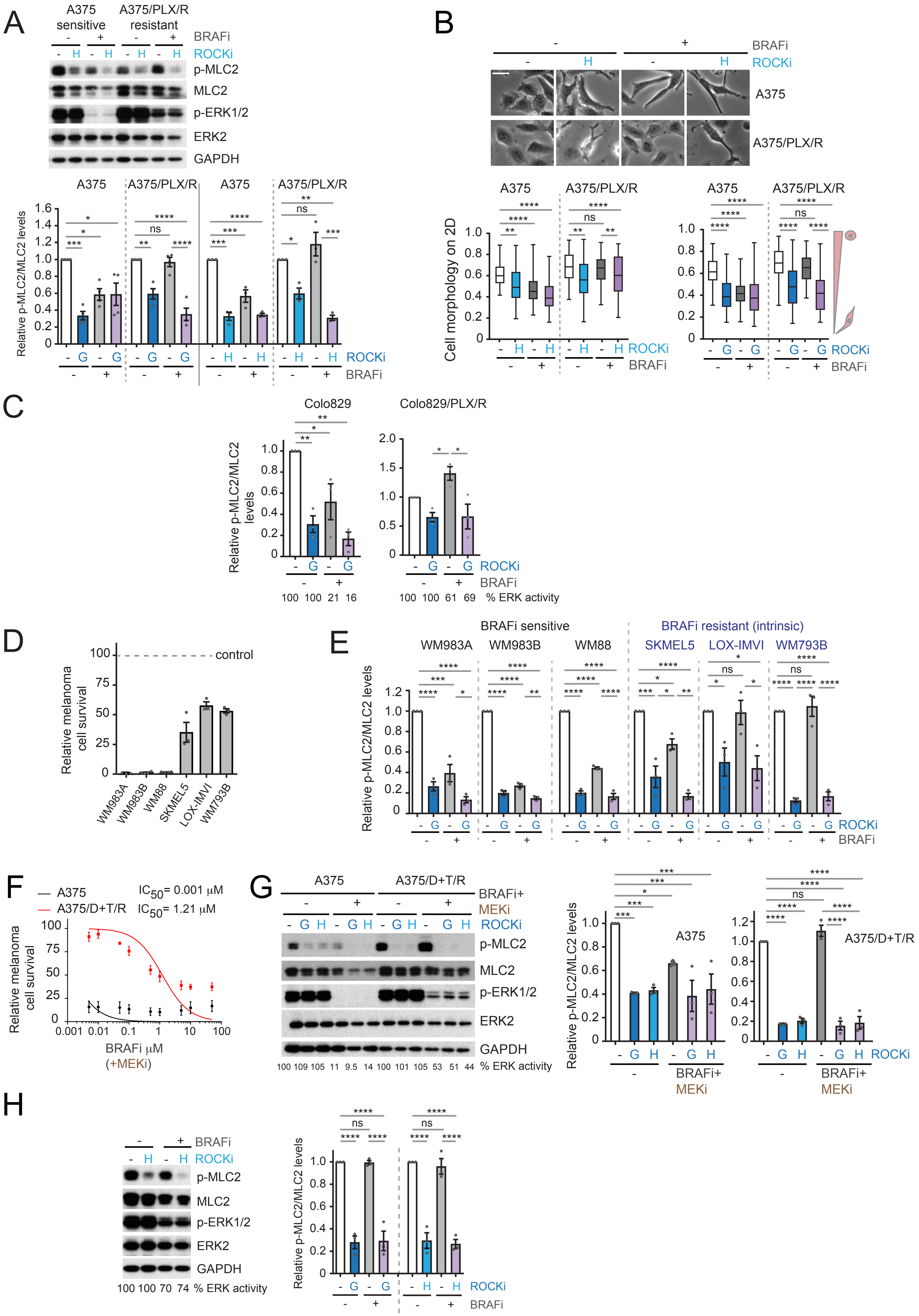
A
A375 sensitive
A375/PLX/R resistant
BRAFi
ROCKi
p-MLC2
MLC2
p-ERK1/2
ERK2
GAPDH
A375
A375/PLX/R
Relative p-MLC2/MLC2 levels
ROCKi
BRAFi
B
BRAFi
ROCKi
A375
A375/PLX/R
Cell morphology on 2D
ROCKi
BRAFi
C
Colo829
Colo829/PLX/R
Relative p-MLC2/MLC2 levels
ROCKi
BRAFi
100 100 21 16
100 100 61 69 % ERK activity
D
control
Relative melanoma cell survival
WM983A
WM983B
WM88
SKMEL5
LOX-IMVI
WM793B
E
BRAFi sensitive
BRAFi resistant (intrinsic)
WM983A
WM983B
WM88
SKMEL5
LOX-IMVI
WM793B
Relative p-MLC2/MLC2 levels
ROCKi
BRAFi
F
A375
A375/D+T/R
IC50= 0.001 μM
IC50= 1.21 μM
Relative melanoma cell survival
BRAFi μM
(+MEKi)
G
A375
A375/D+T/R
BRAFi+
MEKi
ROCKi
p-MLC2
MLC2
p-ERK1/2
ERK2
GAPDH
100 109 105 11 9.5 14 100 101 105 53 51 44 % ERK activity
Relative p-MLC2/MLC2 levels
A375
A375/D+T/R
ROCKi
BRAFi+
MEKi
H
BRAFi
ROCKi
p-MLC2
MLC2
p-ERK1/2
ERK2
GAPDH
100 100 70 74 % ERK activity
Relative p-MLC2/MLC2 levels
ROCKi
BRAFi

Figure S2, related to Figure 3. Therapy-resistant melanomas maintain high ROCK-driven Myosin II levels. (**A**) p-MLC2 immunoblots and quantification after 24 hr treatment with BRAFi PLX4720 and ROCKi GSK269962A (n=4) and H1152 (n=3). (**B**) Images and cell morphology quantification of A375 and A375/PLX/R cells after 24 hr treatment (BRAFi PLX4720; ROCKi GSK269962A, H1152) (n=100 cells pooled from 3 experiments). Scale bar, 50 µm. (**C**) p-MLC2 levels from immunoblotting after 24 hr treatment (BRAFi PLX4720, ROCKi GSK269962A) (n=3). (**D**) Survival after 7-14 day 5 µM PLX4720 treatment. (**E**) p-MLC2 levels after 24 hr treatment (BRAFi PLX4720, ROCKi GSK269962A) (n=3). (**F**) Survival of A375 and A375/D+T/R cells after 6 day treatment (BRAFi Dabrafenib and 10 nM MEKi Trametinib) (n=3). (**G**) p-MLC2 and p-ERK1/2 immunoblots and quantification after 24 hr treatment (BRAFi Dabrafenib, MEKi Trametinib; ROCKi GSK269962A and H1152) (n=3). (**H**) p-MLC2 and p-ERK1/2 immunoblots of Patient #35 cells after 24 hr treatment (BRAFi PLX4720; ROCKi GSK269962A, H1152) (n=3).

Graphs show mean ± SEM and individual data points (circle) except box plot in B (median (center line); interquartile range (box); min-max values (whiskers)). p values by ANOVA with Tukey's correction (A, C, E, G, H), Kruskal-Wallis with Dunn's correction (B). *p<0.05, **p<0.01, ***p<0.001, ****p<0.0001, ns not significant.

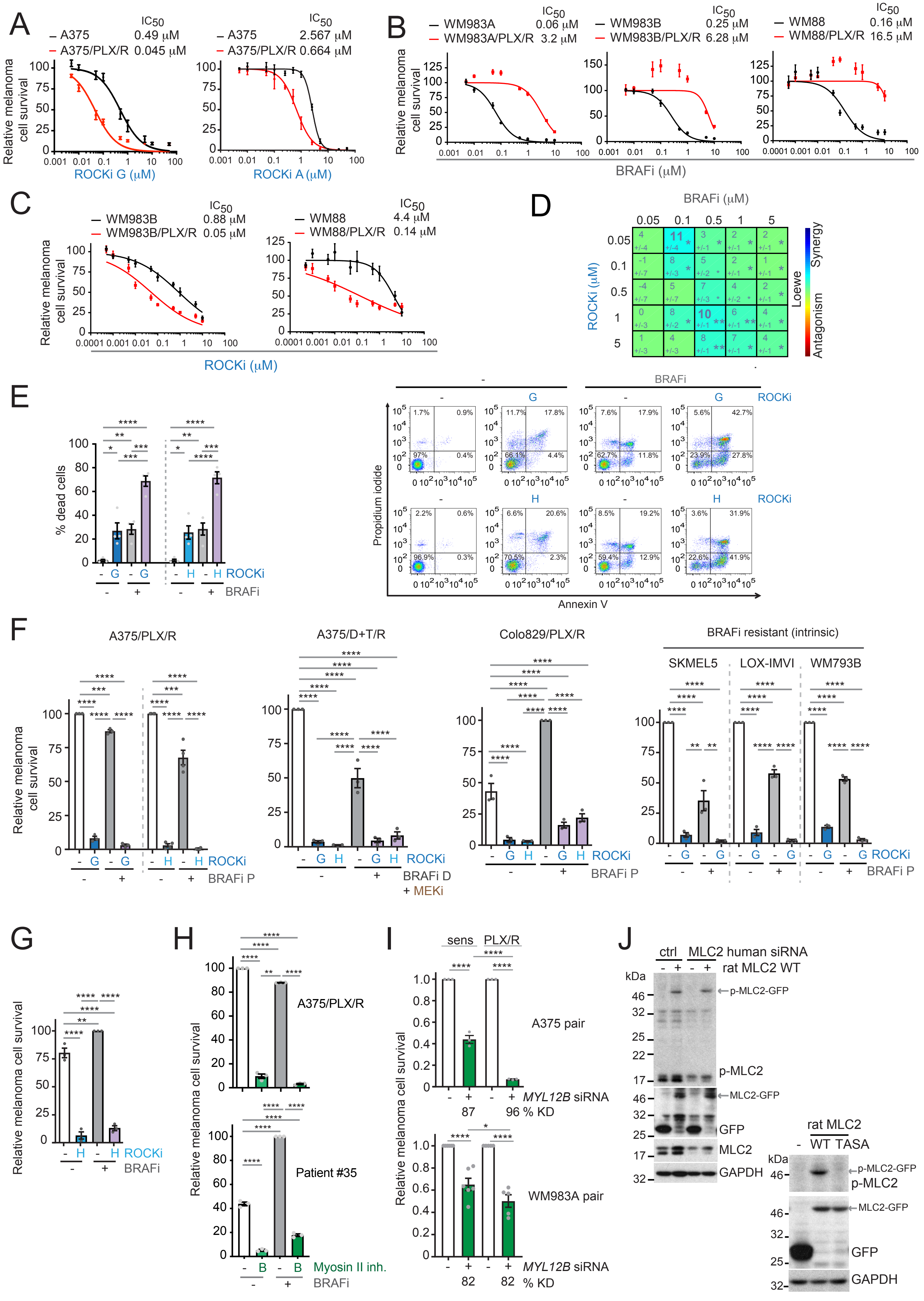
A
A375
A375/PLX/R
IC50
0.49 μM
0.045 μM
2.567 μM
0.664 μM
Relative melanoma cell survival
ROCKi G (μM)
ROCKi A (μM)
B
WM983A
WM983A/PLX/R
0.06 μM
3.2 μM
WM983B
WM983B/PLX/R
0.25 μM
6.28 μM
WM88
WM88/PLX/R
0.16 μM
16.5 μM
BRAFi (μM)
C
WM983B
WM983B/PLX/R
0.88 μM
0.05 μM
WM88
WM88/PLX/R
4.4 μM
0.14 μM
ROCKi (μM)
D
BRAFi (μM)
ROCKi (μM)
Synergy
Loewe
Antagonism
E
% dead cells
ROCKi
BRAFi
Propidium iodide
Annexin V
F
A375/PLX/R
A375/D+T/R
Colo829/PLX/R
BRAFi resistant (intrinsic)
SKMEL5
LOX-IMVI
WM793B
ROCKi
BRAFi P
BRAFi D
+ MEKi
G
Relative melanoma cell survival
ROCKi
BRAFi
H
A375/PLX/R
Patient #35
Relative melanoma cell survival
Myosin II inh.
BRAFi
I
sens
PLX/R
A375 pair
WM983A pair
Relative melanoma cell survival
MYL12B siRNA
87
96 % KD
82
82 % KD
J
ctrl
MLC2 human siRNA
rat MLC2 WT
kDa
p-MLC2-GFP
p-MLC2
MLC2-GFP
GFP
MLC2
GAPDH
rat MLC2
WT
TASA

Figure S3, related to Figure 3. Survival of targeted therapy-resistant melanomas is dependent on ROCK-driven Myosin II activity. (**A**) Survival after 3 day treatment (ROCKi GSK269962A, AT13148) (n=3). In GSK269962A experiments, resistant cells were cultured without PLX4720. (**B-C**) Survival after 3-5 day treatment (BRAFi PLX4720, ROCKi GSK269962A) (n=3-5). (**D**) Average synergy scores of ROCKi GSK269962A and BRAFi PLX4720 treatments of A375 cells (n=4) (*p<0.05; **p<0.001). (**E**) % of dead A375 cells by annexin V/PI staining after 3 day treatment (BRAFi PLX4720, ROCKi GSK269962A and H1152) (n=4). Representative FACS plots are shown. (**F**) Survival of A375/PLX/R (n=3, GSK269962A; n=4, H1152); A375/D+T/R (n=3); Colo829/PLX/R (n=3); and BRAFi-resistant cell lines (n=3) after treatments for 5 days (A375/PLX/R), 10 days (A375/D+T/R, Colo829/PLX/R) and 7-14 days (SKMEL5, LOX-IMVI, WM793B). BRAFi PLX4720 for A375/PLX/R, Colo829/PLX/R and intrinsic panel; BRAFi Dabrafenib and MEKi trametinib for A375/D+T/R; ROCKi GSK269962A, H1152. (**G**) Survival of Patient #35 cells after 10 day treatment (ROCKi H1152, BRAFi PLX4720) (n=3). (**H**) Survival after 5-10 day treatment (BRAFi PLX4720, Blebbistatin) (n=3). Data for cells under PLX4720 (+/- blebbistatin) is also shown in Figure 3L. (**I**) Survival 8 days after *MYL12B* KD (n=3 A375 pair; n=6 WM983A pair). Resistant cells were grown in the presence of 1 μM PLX4720 for the whole experiment. mRNA KD (% decrease vs control) by qRT-PCR is shown. (**J**) GFP, p-MLC2 immunoblots of A375/PLX/R cells after MLC2 knockdown and rescue with rat MLC2 (WT or TASA mutant). Graphs show mean ± SEM and individual data points (circle). p values by ANOVA with Tukey's correction (E-I), *p<0.05, **p<0.01, ***p<0.001, ****p<0.0001, ns not significant.

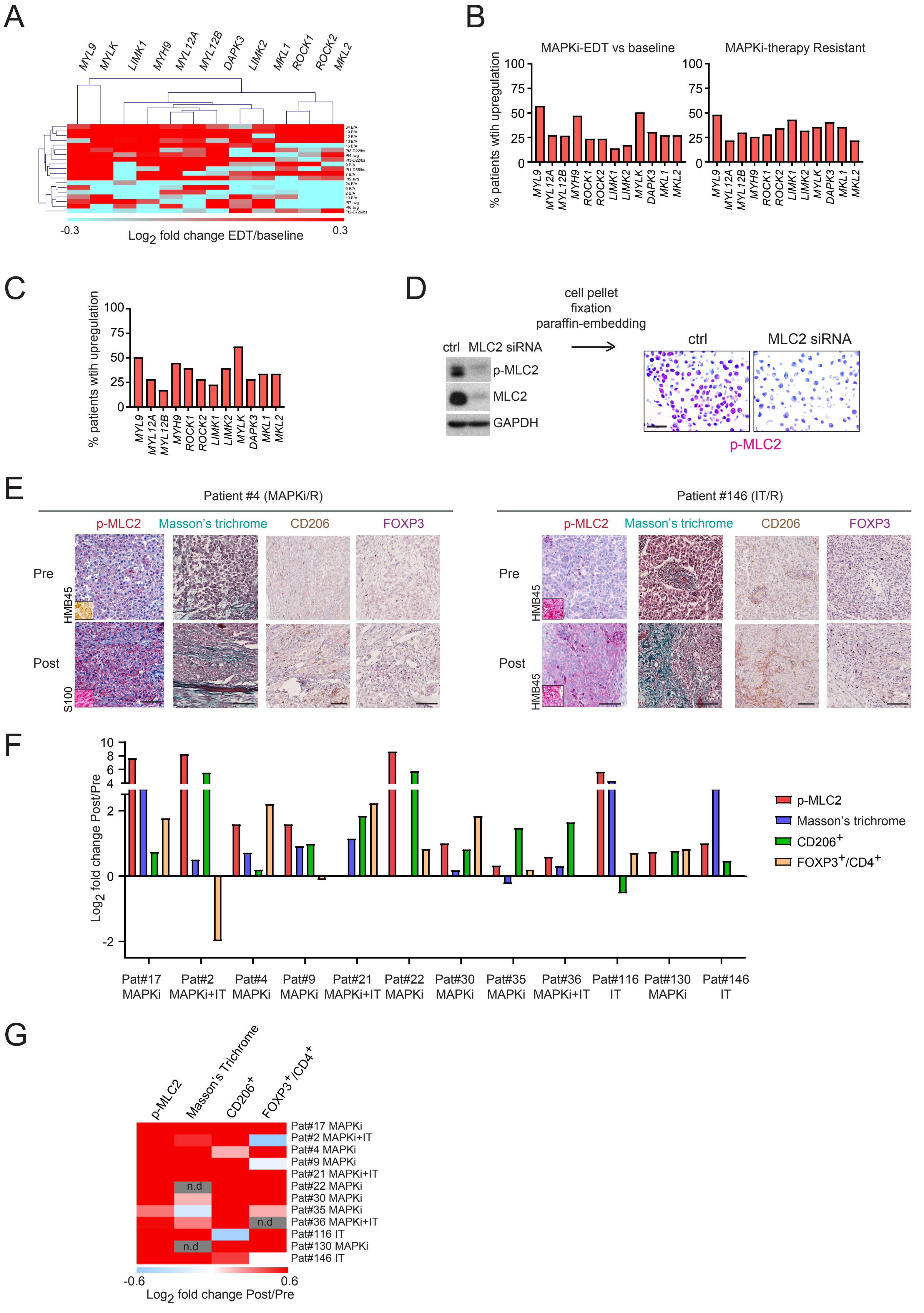
A
MYL9
MYLK
LIMK1
MYH9
MYL12A
MYL12B
DAPK3
LIMK2
MKL1
ROCK1
ROCK2
MKL2
-0.3
0.3
Log2 fold change EDT/baseline
B
MAPKi-EDT vs baseline
MAPKi-therapy Resistant
% patients wtih upregulation
C
% patients wtih upregulation
D
cell pellet
fixation
paraffin-embedding
ctrl
MLC2 siRNA
p-MLC2
MLC2
GAPDH
E
Patient #4 (MAPKi/R)
Patient #146 (IT/R)
p-MLC2
Masson's trichrome
CD206
FOXP3
Pre
Post
HMB45
S100
F
Log2 fold change Post/Pre
p-MLC2
Masson's trichrome
CD206+
FOXP3+/CD4+
Pat#17 MAPKi
Pat#2 MAPKi+IT
Pat#4 MAPKi
Pat#9 MAPKi
Pat#21 MAPKi+IT
Pat#22 MAPKi
Pat#30 MAPKi
Pat#35 MAPKi
Pat#36 MAPKi+IT
Pat#116 IT
Pat#130 MAPKi
Pat#146 IT
G
p-MLC2
Masson's Trichrome
CD206+
FOXP3+/CD4+
n.d
-0.6
0.6
Log2 fold change Post/Pre

Figure S4, related to Figure 4. High Myosin II levels identify therapy-resistant melanomas in human samples. (**A**) Heatmap of fold change in expression of ROCK-Myosin II pathway genes in paired MAPKi-early during treatment (EDT) vs baseline patient samples from (Kwong et al., 2015; Song et al., 2017). (**B**) % of patients with upregulation (>1.3fold) of indicated genes in EDT samples vs baseline (left graph) (Kakavand et al., 2017; Kwong et al., 2015; Long et al., 2014; Rizos et al., 2014; Song et al., 2017); and in Resistant samples vs baseline (right graph) (Hugo et al., 2015; Kakavand et al., 2017; Kwong et al., 2015; Long et al., 2014; Rizos et al., 2014; Sun et al., 2014; Wagle et al., 2014). (**C**) % of patients with upregulation (>1.3fold) of indicated genes in samples on anti-PD-1 vs baseline from (Riaz et al., 2017). (**D**) Validation of p-MLC2 antibody in paraffin-embedded cell pellets of A375/PLX/R cells transfected with siRNA against MLC2. Non-targeting siRNA was used as control. Left, p-MLC2 and MLC2 immunoblots. Right, photomicrographs of p-MLC2 staining in cell pellet sections. Scale bar, 100 µm. (**E**) Photomicrographs of p-MLC2, Masson's Trichrome, CD206 and FOXP3 staining in representative paired samples from patients before and after therapy. Insets show IHC staining with melanoma markers HMB45/Melan-A or S100. Scale bars, 100 µm. (**F**) Fold change in levels of p-MLC2, Masson's, $CD206^{+}$ and ratio $FOXP3^{+}/CD4^{+}$ in 12 post- vs pre-treatment samples (including those in S4E and Figures 4H-K). (**G**) Heatmap of fold change in levels of indicated markers in post vs pre-treatment samples from S4F. n.d., not determined.

Table S5, related to Figure 4. List of gene signatures from ssGSEA.

Gene signature	Process
GOTZMANN_EPITHELIAL_TO_MESENCHYMAL_TRANSITION_UP	EMT/metastasis
ANASTASSIOU_CANCER_MESENCHYMAL_TRANSITION_SIGNATURE	EMT/metastasis
HALLMARK_EPITHELIAL_MESENCHYMAL_TRANSITION	EMT/metastasis
WU_CELL_MIGRATION	EMT/metastasis
LU_TUMOR_ANGIOGENESIS_UP	angiogenesis
LU_TUMOR_ENDOTHELIAL_MARKERS_UP	angiogenesis
LU_TUMOR_VASCULATURE_UP	angiogenesis
CLASPER_LYMPHATIC_VESSELS_DURING_METASTASIS_UP	angiogenesis
MANALO_HYPOXIA_UP	hypoxia
QI_HYPOXIA	hypoxia
ROY_WOUND_BLOOD_VESSEL_UP	wound healing
KEGG_TGF_BETA_SIGNALING_PATHWAY	TGFβ
COULOUARN_TEMPORAL_TGFB1_SIGNATURE_UP	TGFβ
AZARE_NEOPLASTIC_TRANSFORMATION_BY_STAT3_UP	STAT3
HALLMARK_IL6_JAK_STAT3_SIGNALING	STAT3
DAUER_STAT3_TARGETS_UP	STAT3
HALLMARK_INFLAMMATORY_RESPONSE	NF-κB
SCHOEN_NFKB_SIGNALING	NF-κB
HINATA_NFKB_TARGETS_FIBROBLAST_UP	NF-κB
HALLMARK_TNFA_SIGNALING_VIA_NFKB	NF-κB
CORDENONSI_YAP_CONSERVED_SIGNATURE	YAP

Table S6, related to Figure 4. Summary of clinical data of patient samples used for stainings.

Patient#	Mutations	Treatment	Pre-Treatment Block Tissue	Post-Treatment Block tissue
#17	$BRAF^{V600E}$ $NRAS^{Q61R}$	dabrafenib+trametinib	left axillary dissection	left axillary nodal mass
#4	$BRAF^{V600E}$	vemurafenib	nodule left breast	excision of cutaneous lesion
#2	$BRAF^{V600E}$	vemurafenib, ipilimumab	right chest	skin lesion to chest wall
#35	$BRAF^{V600E}$ $NRAS^{Q61R}$	vemurafenib	dorsum, right hand	right axillary lymph node
#9	$BRAF^{V600E}$	vemurafenib	left calf	left groin lymph node dissection
#21	$BRAF^{V600E}$	vemurafenib,+3 day pembrolizumab	right lower leg	right lower leg
#22	$BRAF^{V600E}$	vemurafenib	right dorsum of foot	right upper thigh tumour
#30	$BRAF^{V600E}$	vemurafenib dabtram	left ear lobe	left postauricular region
#36	$BRAF^{V600E}$	vemurafenib, ipilimumab	left neck	right deltoid melanoma deposit
#116	$NRAS^{Q61R}$	ipilimumab	right inguinal node dissection	proximal small bowel resection.
#130	$BRAF^{V600E}$	vemurafenib, dabrafenib+trametinib	excised from right flank	right frontal craniotomy
#146	BRAF Wildtype	ipilimumab	right Ileoinguinal node dissection	right external oblique. Right internal oblique mass

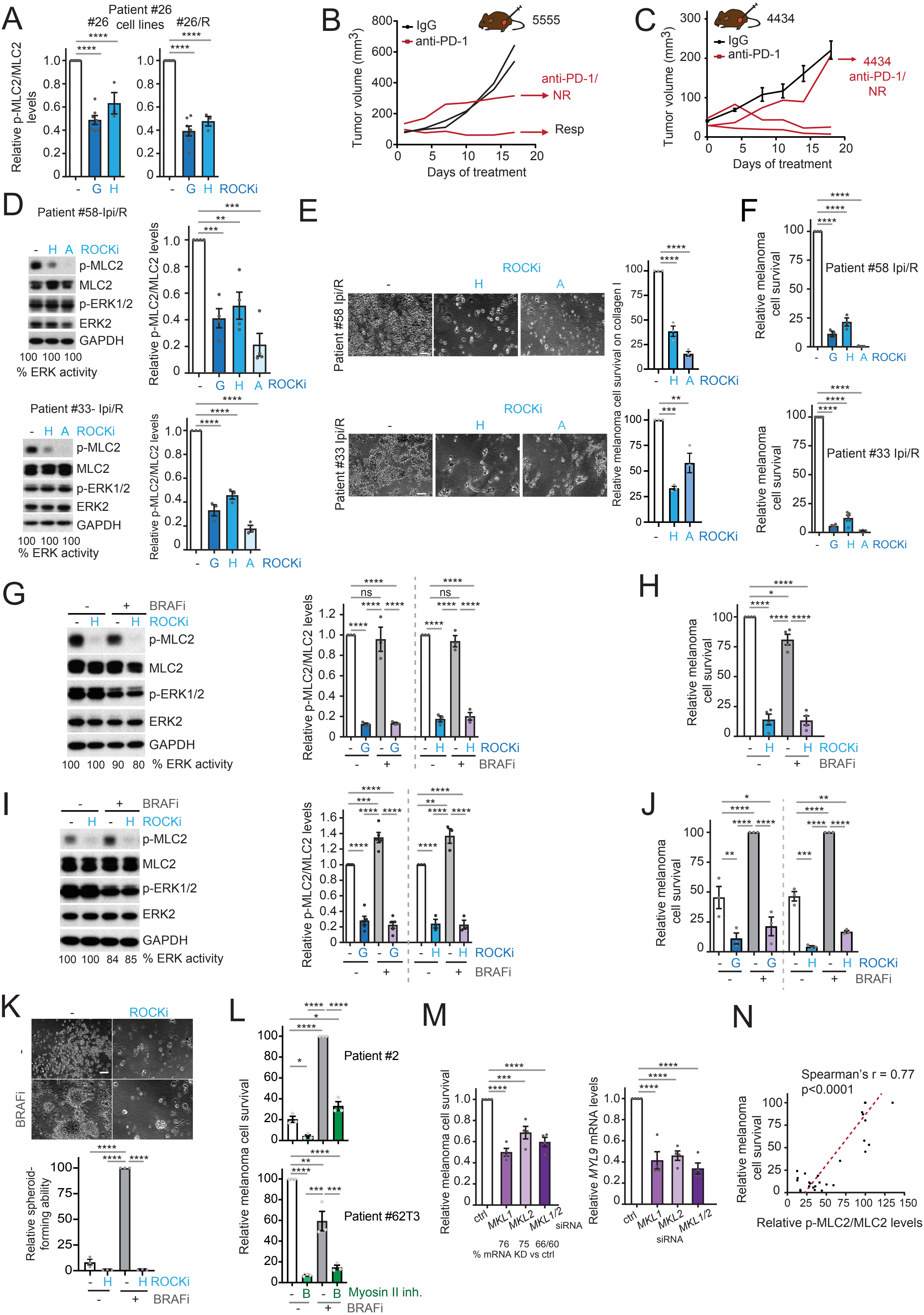
A
Patient #26 cell lines
#26
#26/R
Relative p-MLC2/MLC2 levels
- G H ROCKi
B
5555
IgG
anti-PD-1
Tumor volume (mm3)
anti-PD-1/ NR
Resp
Days of treatment
C
4434
IgG
anti-PD-1
4434 anti-PD-1/ NR
Tumor volume (mm3)
Days of treatment
D
Patient #58-Ipi/R
- H A ROCKi
p-MLC2
MLC2
p-ERK1/2
ERK2
GAPDH
100 100 100
% ERK activity
Relative p-MLC2/MLC2 levels
- G H A ROCKi
Patient #33- Ipi/R
- H A ROCKi
p-MLC2
MLC2
p-ERK1/2
ERK2
GAPDH
100 100 100
% ERK activity
E
ROCKi
- H A
Patient #58 Ipi/R
Patient #33 Ipi/R
Relative melanoma cell survival on collagen I
- H A ROCKi
F
Patient #58 Ipi/R
Patient #33 Ipi/R
Relative melanoma cell survival
- G H A ROCKi
G
- + BRAFi
- H - H ROCKi
p-MLC2
MLC2
p-ERK1/2
ERK2
GAPDH
100 100 90 80 % ERK activity
Relative p-MLC2/MLC2 levels
ns
- G - G - H - H ROCKi
- + - + BRAFi
H
Relative melanoma cell survival
- H - H ROCKi
- + BRAFi
I
- + BRAFi
- H - H ROCKi
p-MLC2
MLC2
p-ERK1/2
ERK2
GAPDH
100 100 84 85 % ERK activity
Relative p-MLC2/MLC2 levels
- G - G - H - H ROCKi
- + - + BRAFi
J
Relative melanoma cell survival
- G - G - H - H ROCKi
- + - + BRAFi
K
- ROCKi
-
BRAFi
Relative spheroid-forming ability
- H - H ROCKi
- + BRAFi
L
Patient #2
Patient #62T3
Relative melanoma cell survival
- B - B Myosin II inh.
- + BRAFi
M
Relative melanoma cell survival
ctrl MKL1 MKL2 MKL1/2 siRNA
76 75 66/60
% mRNA KD vs ctrl
Relative MYL9 mRNA levels
ctrl MKL1 MKL2 MKL1/2
siRNA
N
Spearman's r = 0.77
p<0.0001
Relative melanoma cell survival
Relative p-MLC2/MLC2 levels

Figure S5, related to Figure 5. ROCK-driven Myosin II activity in immunotherapy-resistant melanoma. (**A**) p-MLC2 levels after 24 hr treatments (n=7 GSK269962A (G); n=3 H1152 (H)). Immunoblots are in Figure 5A. (**B**) Growth of Braf^{V600E} mouse 5555 allografts after anti-PD-1 or IgG isotype treatment. Indicated cell lines were established from anti-PD-1/non-responder (NR) and anti-PD-1/responder (Resp) 5555 tumors. (**C**) Growth of Braf^{V600E} mouse 4434 allografts after treatment. Indicated cell lines were established from anti-PD-1/NR tumors. (**D**) p-MLC2 immunoblots and quantification of Patient #58 cells (n=4) and Patient #33 (n=3) cells after 24 hr ROCKi treatment (GSK269962A (G); H1152 (H); AT13148 (A)). (**E**) Images and quantification of cell survival on collagen I for 9-14 days (n=3). Scale bars, 100 µm. (**F**) Survival after 9-14 day treatment (n=5 #33; n=3 #58). (**G**) p-MLC2 immunoblots and quantification of Patient #62T3 cells after 24 hr ROCKi treatment (ROCKi H1152 and GSK269962A, BRAFi PLX4720) (n=3). (**H**) Survival of Patient #62T3 cells after 10 day treatment (ROCKi H1152, BRAFi PLX4720) (n=4). (**I**) p-MLC2 immunoblots and quantification of Patient #2 cells after 24 hr treatment (ROCKi H1152 and GSK269962A; BRAFi PLX4720) (n=5 GSK269962A; n=3 H1152). (**J**) Survival of Patient #2 cells after 6 day treatment (ROCKi GSK269962A and H1152; BRAFi PLX4720) (n=3). (**K**) Spheroid-forming ability of Patient #2 cells on collagen I after 16 day treatment (ROCKi H1152, BRAFi PLX4720) (n=3). Scale bar, 100 µm. (**L**) Survival after 10 day treatment (Myosin II inhibitor Blebbistatin, BRAFi PLX4720) (n=3). Data for cells under PLX4720 (+/- blebbistatin) is also shown in main Figure 5K. (**M**) Survival (left) and MLC2 (*MYL9*) expression (right) in Patient #2 cells after MRTF (*MKL*) depletion (n=4). Expression and % of knockdown by qRT-PCR is shown. (**N**) Scatter plot of average relative p-MLC2/MLC2 levels and average relative survival in resistant cells. Red line indicates Deming linear regression analysis. Correlation (Spearman's r) between p-MLC2 and survival is also shown.

Graphs show mean ± SEM and individual data points (circle). p values by ANOVA with Dunnett's (A, D-F, M) or Tukey's correction (G-L) and Spearman's r (N).

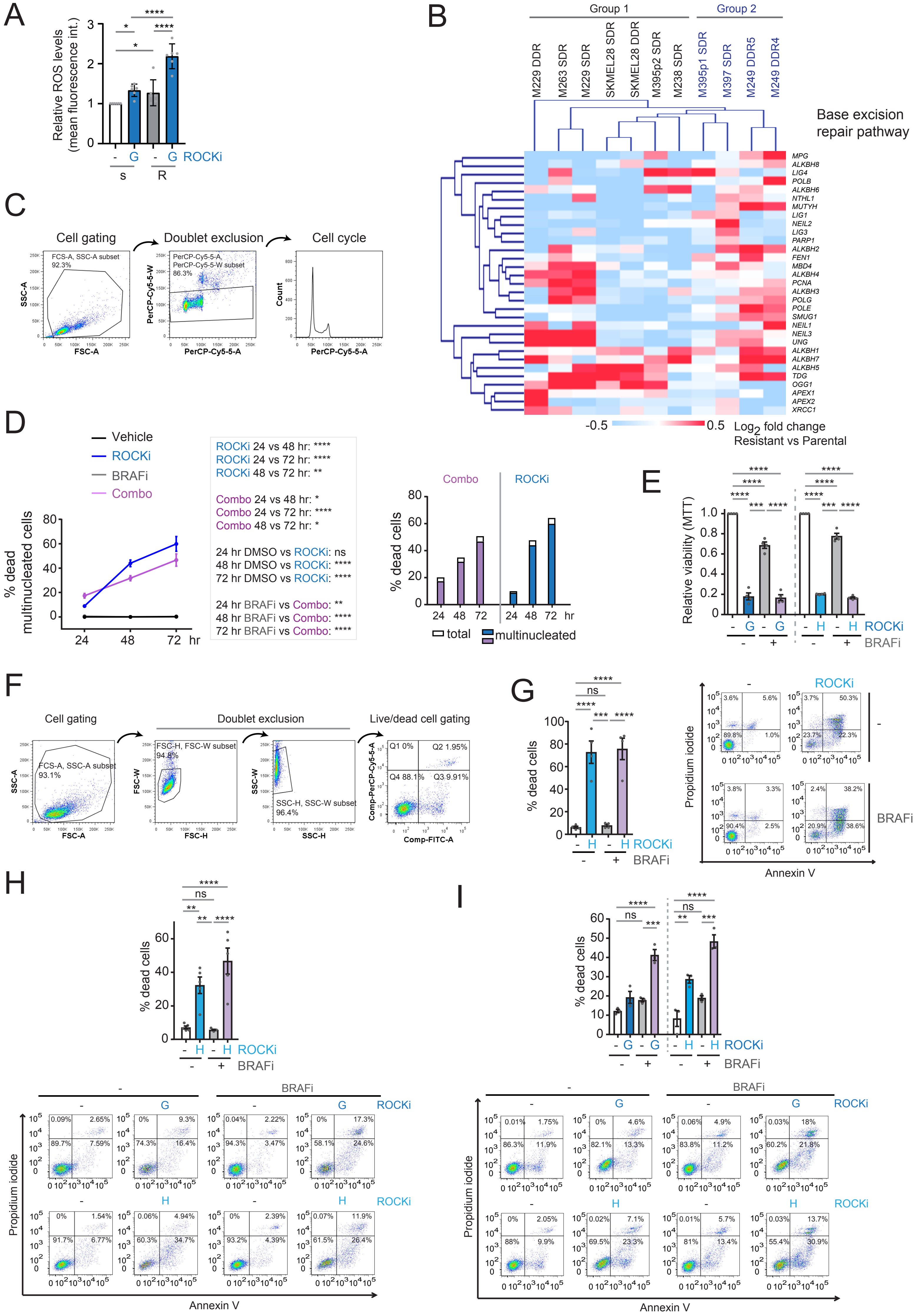

A
Relative ROS levels (mean fluorescence int.)
ROCKi
B
Group 1
Group 2
M229 DDR
M263 SDR
M229 SDR
SKMEL28 SDR
SKMEL28 DDR
M395p2 SDR
M238 SDR
M395p1 SDR
M397 SDR
M249 DDR5
M249 DDR4
Base excision repair pathway
MPG
ALKBH8
LIG4
POLB
ALKBH6
NTHL1
MUTYH
LIG1
NEIL2
LIG3
PARP1
ALKBH2
FEN1
MBD4
ALKBH4
PCNA
ALKBH3
POLG
POLE
SMUG1
NEIL1
NEIL3
UNG
ALKBH1
ALKBH7
ALKBH5
TDG
OGG1
APEX1
APEX2
XRCC1
Log2 fold change Resistant vs Parental
C
Cell gating
Doublet exclusion
Cell cycle
D
Vehicle
ROCKi
BRAFi
Combo
% dead multinucleated cells
ROCKi 24 vs 48 hr: ****
ROCKi 24 vs 72 hr: ****
ROCKi 48 vs 72 hr: **
Combo 24 vs 48 hr: *
Combo 24 vs 72 hr: ****
Combo 48 vs 72 hr: *
24 hr DMSO vs ROCKi: ns
48 hr DMSO vs ROCKi: ****
72 hr DMSO vs ROCKi: ****
24 hr BRAFi vs Combo: **
48 hr BRAFi vs Combo: ****
72 hr BRAFi vs Combo: ****
% dead cells
total
multinucleated
E
Relative viability (MTT)
BRAFi
F
Cell gating
Doublet exclusion
Live/dead cell gating
G
% dead cells
Propidium iodide
Annexin V
H
I

Figure S6, related to Figure 6. ROCK-Myosin II inhibition induces lethal reactive oxygen species (ROS), DNA damage and cell cycle arrest. (**A**) ROS levels in WM983A (s) and WM983A/PLX/R (R) cells after 48 hr ROCKi GSK269962A treatment (n= 6). (**B**) Heatmap of fold change in expression of genes in the base excision repair pathway in resistant vs parental cell lines from (Song et al., 2017). (**C**) Gating strategy for cell cycle experiments. (**D**) Left, % of dead multinucleated cells after 72 hr treatment (ROCKi GSK269962A, BRAFi PLX4720) of A375/PLX/R cells and imaged with time-lapse video-microscopy (n=6 movies/treatment pooled from 2 independent experiments). Right, average % of total dead cells and dead multinucleated cells. (**E**) Viability of A375/PLX/R cells by MTT assay after 72 hr treatment (ROCKi GSK269962A and H1152; BRAFi PLX4720) (n=4). (**F**) Gating strategy for annexin V/propidium iodide (PI) experiments. (**G-I**) % of dead cells measured by annexin V/PI staining after 72 hr treatment (ROCKi GSK269962A and H1152; BRAFi PLX4720) of: A375/PLXR (n=4) (**G**); Patient #2 (n=5) (**H**); Patient #35 (n=3) (**I**). Representative FACS plots are shown. Graphs show mean ± SEM and individual data points (circle). p values were estimated by one-way ANOVA with Tukey's correction (E, G-I), Benjamini, Krieger and Yekutieli correction (A) and two-way ANOVA with Tukey's correction (D), *$p<0.05$, **$p<0.01$, ***$p<0.001$, ****$p<0.0001$, ns not significant.

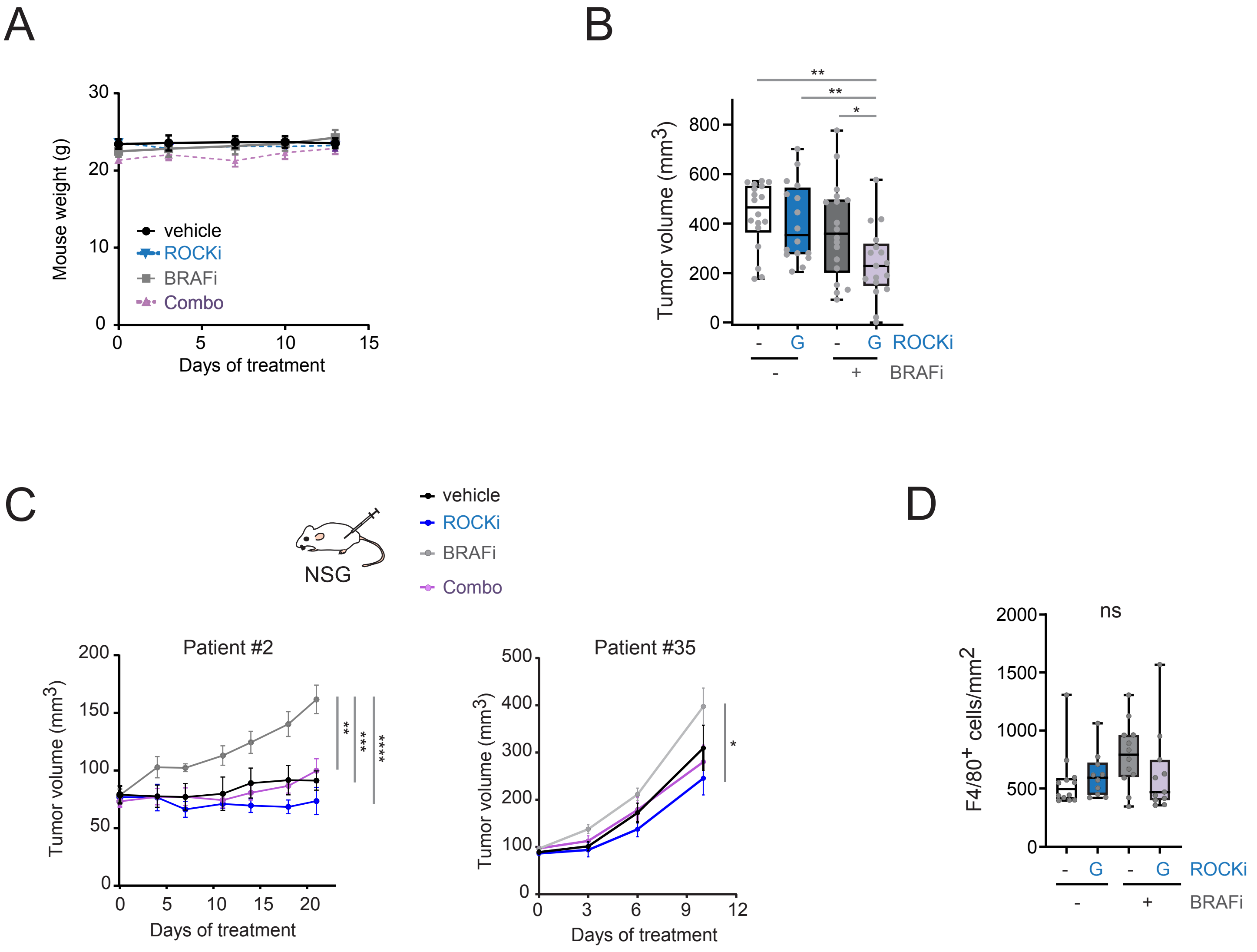
A
B
C
D
Mouse weight (g)
Days of treatment
vehicle
ROCKi
BRAFi
Combo
Tumor volume (mm3)
ROCKi
BRAFi
NSG
Patient #2
Patient #35
ns
F4/80+ cells/mm2

Figure S7, related to Figure 7. Combining ROCK inhibitors with BRAF inhibitors *in vivo*. (**A**) Weight of nude mice xenografted with A375/PLX/R after treatment. (**B**) Tumor volume at endpoint of A375/PLX/R xenografts. Pooled data from 3 independent experiments are shown. (**C**) Growth of (left) Patient #2 and (right) Patient #62T3 xenografts in NSG mice after treatments. (**D**) Quantification of F4/80$^+$ cells in A375/PLX/R xenografts.
A-D: ROCKi GSK269962A, BRAFi PLX4720.
A, C show mean ± SEM. B, D show box plots with median (center line); interquartile range (box); min-max (whiskers); and individual mice (circles). p values by ANOVA with Benjamini, Krieger and Yekutieli (B, D), Dunnett's (C, right) or Tukey's (C, left) correction.

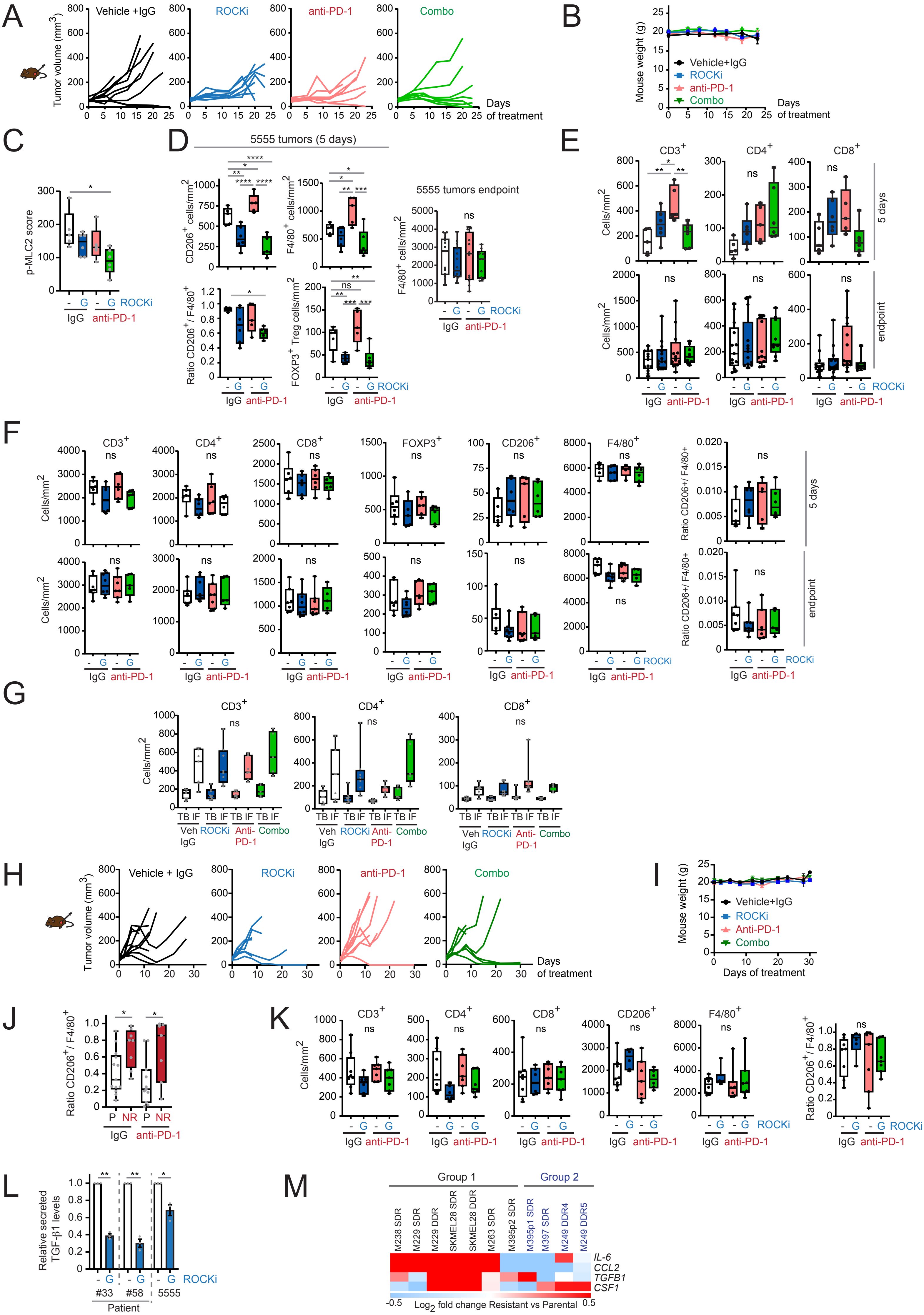

A
Vehicle +IgG
ROCKi
anti-PD-1
Combo
Tumor volume (mm3)
Days of treatment
B
Mouse weight (g)
Vehicle+IgG
ROCKi
anti-PD-1
Combo
Days of treatment
C
p-MLC2 score
- G - G ROCKi
IgG anti-PD-1
D
5555 tumors (5 days)
CD206+ cells/mm2
F4/80+ cells/mm2
Ratio CD206+/ F4/80+
FOXP3+ Treg cells/mm2
5555 tumors endpoint
F4/80+ cells/mm2
ns
- G - G ROCKi
IgG anti-PD-1
E
CD3+
CD4+
CD8+
Cells/mm2
5 days
endpoint
ns
- G - G ROCKi
IgG anti-PD-1
F
CD3+
CD4+
CD8+
FOXP3+
CD206+
F4/80+
Ratio CD206+/ F4/80+
Cells/mm2
5 days
endpoint
ns
- G - G ROCKi
IgG anti-PD-1
G
CD3+
CD4+
CD8+
Cells/mm2
TB IF TB IF TB IF TB IF
Veh IgG ROCKi Anti-PD-1 Combo
ns
H
Vehicle + IgG
ROCKi
anti-PD-1
Combo
Tumor volume (mm3)
Days of treatment
I
Mouse weight (g)
Vehicle+IgG
ROCKi
Anti-PD-1
Combo
Days of treatment
J
Ratio CD206+/ F4/80+
P NR P NR
IgG anti-PD-1
K
CD3+
CD4+
CD8+
CD206+
F4/80+
Cells/mm2
Ratio CD206+/ F4/80+
ns
- G - G ROCKi
IgG anti-PD-1
L
Relative secreted TGF-β1 levels
- G - G - G ROCKi
#33 #58 5555
Patient
M
Group 1
Group 2
M238 SDR
M229 SDR
M229 DDR
SKMEL28 SDR
SKMEL28 DDR
M263 SDR
M395p2 SDR
M395p1 SDR
M397 SDR
M249 DDR4
M249 DDR5
IL-6
CCL2
TGFB1
CSF1
-0.5
Log2 fold change Resistant vs Parental
0.5

Figure S8, related to Figure 8. ROCK-Myosin II inhibition improves efficacy of immune checkpoint inhibitors. (**A**) Growth of $Braf^{V600E}$ mouse 5555 allografts in C57BL/6J after treatment. (**B**) Mouse weights from A. (**C**) Quantification of p-MLC2 in 5555 tumors from A at 5 days of treatment. (**D**) Quantification of $CD206^{+}$, $FOXP3^{+}$ and $F4/80^{+}$ cells in 5555 tumors from A at 5 days of treatment (left); and $F4/80^{+}$ at endpoint (right). (**E**) Quantification of $CD3^{+}$, $CD4^{+}$ and $CD8^{+}$ cells in 5555 tumors from A. (**F**) Quantification of $CD3^{+}$, $CD4^{+}$, $CD8^{+}$, $FOXP3^{+}$, $CD206^{+}$ and $F4/80^{+}$ cells in spleens from 5555-bearing mice from A. (**G**) Regional analysis of immune populations in tumor body (TB) and invasive front (IF) of 5555 tumors from A at endpoint. (**H**) Growth of 5555-anti-PD-1/NR allografts after treatments. (**I**) Mouse weights from H. (**J**) Ratio $CD206^{+}/F4/80^{+}$ in parental 5555 (P) and 5555-anti-PD-1/NR (NR) tumors under IgG or anti-PD-1 treatment. (**K**) Quantification of $CD3^{+}$, $CD4^{+}$, $CD8^{+}$, $CD206^{+}$, $F4/80^{+}$ cells and ratio $CD206^{+}/F4/80^{+}$ in 5555-anti-PD-1/NR tumors from H. (**L**) TGF-β1 protein levels by ELISA after 48 hr treatment. (**M**) Heatmap of fold change in expression of indicated immunosuppressive cytokines in MAPKi-resistant vs parental cell lines from (Song et al., 2017).

A-I, K-L: ROCKi GSK269962A.

B, I, L show mean ± SEM. Box plots show median (center line); interquartile range (box); min-max (whiskers); and individual mice (circles). 5555 endpoint tumors in D-E include pooled data from 2 independent experiments.

P-values by ANOVA with Tukey's (G) or Benjamini, Krieger and Yekutieli correction (C-F, J-K), t-test with Welch's correction (L).

For G, statistical analysis of IF between groups: not significant. Statistical analysis $CD3^{+}$ TB vs IF: veh ($p=0.0063$), ROCKi ($p=0.0016$), anti-PD-1 ($p=0.01$), combo ($p=0.0009$). Statistical analysis $CD4^{+}$ TB vs IF: combo ($p=0.04$), not significant for the other groups. Statistical analysis $CD8^{+}$ TB vs IF: anti-PD-1 ($p=0.0037$), not significant for the other groups. *$p<0.05$, **$p<0.01$, ***$p<0.001$, ****$p<0.0001$, ns not significant.

Table S7, related to STAR Methods. List of RNAi sequences.

Target	RNAi sequences
Non-targeting Control siRNA	ON-TARGET (OT) plus non-targeting siRNA #1:<br>5'-UGGUUUACAUGUCGACUAA-3'
MKL1	OT SmartPool:<br>OT#5: 5'-GAUCGGAGCUGGUCAGGAU-3'<br>OT#6: 5'-GACAGAGGACUAUCUCAAA-3'<br>OT#7: 5'-GAACUAUCCCAAAGUAGCA-3'<br>OT#8: 5'-AAACUGAGCUGAUUGAGCG-3'
MKL2	OT SmartPool:<br>OT#5: 5'-GGACUAACGUCACAGAUUU -3'<br>OT#6: 5'- CAGCAGCCCUUUAUCAAUA -3'<br>OT#7: 5'-UCAGGGCGAUUUCUCAUUU-3'<br>OT#8: 5'-GAACCUAUGGGCAGUUUAU-3'
MYL9	OT#5: 5'-CCAAGGAUAAAGACGACUA-3'
MYH9	OT SmartPool:<br>OT#5: 5'-GUAUCAAUGUGACCGAUUU-3'<br>OT#6: 5'-CAAAGGAGCCCUGGCGUUA-3'<br>OT#7: 5'-GGAGGAACGCCGAGCAGUA-3'<br>OT#8: 5'-CGAAGCGGGUGAAAGCAAA-3'
MYL12B	OT SmartPool:<br>OT#9: 5'-CCACUUAGCACUUGUAUAA-3'<br>OT#10: 5'-GGGUGUAAAUUGUAUUGAA-3'<br>OT#11: 5'-CCUCAUAGAACCUGUUGCA-3'<br>OT#12: 5'-UGUAUUUAUUCCAGACCUU-3'
ROCK1	OT#8: 5'-CCAGGAAGGUAUAUGCUAU-3'
ROCK2	OT#8: 5'-GAAACUAAUAGGACACUAA-3'
